# Challenges of modelling approaches for network meta-analysis of time-to-event outcomes in the presence of non-proportional hazards to aid decision making: Application to a melanoma network

**DOI:** 10.1177/09622802211070253

**Published:** 2022-01-19

**Authors:** Suzanne C Freeman, Nicola J Cooper, Alex J Sutton, Michael J Crowther, James R Carpenter, Neil Hawkins

**Affiliations:** 1 Department of Health Sciences, 4488 University of Leicester, Leicester, UK; 2 Department of Medical Epidemiology & Biostatistics, 211741 Karolinska Institutet, Stockholm, Sweden; 3 4919 MRC Clinical Trials Unit at UCL, London, UK; 4 4906 London School of Hygiene & Tropical Medicine, London, UK; 5 Health Economics & Health Technology Assessment, 3526 University of Glasgow, Glasgow, UK

**Keywords:** Network meta-analysis, time-to-event outcomes, non-proportional hazards, decision making, Bayesian

## Abstract

**Background:**

Synthesis of clinical effectiveness from multiple trials is a well-established component of decision-making. Time-to-event outcomes are often synthesised using the Cox proportional hazards model assuming a constant hazard ratio over time. However, with an increasing proportion of trials reporting treatment effects where hazard ratios vary over time and with differing lengths of follow-up across trials, alternative synthesis methods are needed.

**Objectives:**

To compare and contrast five modelling approaches for synthesis of time-to-event outcomes and provide guidance on key considerations for choosing between the modelling approaches.

**Methods:**

The Cox proportional hazards model and five other methods of estimating treatment effects from time-to-event outcomes, which relax the proportional hazards assumption, were applied to a network of melanoma trials reporting overall survival: restricted mean survival time, generalised gamma, piecewise exponential, fractional polynomial and Royston-Parmar models.

**Results:**

All models fitted the melanoma network acceptably well. However, there were important differences in extrapolations of the survival curve and interpretability of the modelling constraints demonstrating the potential for different conclusions from different modelling approaches.

**Conclusion:**

The restricted mean survival time, generalised gamma, piecewise exponential, fractional polynomial and Royston-Parmar models can accommodate non-proportional hazards and differing lengths of trial follow-up within a network meta-analysis of time-to-event outcomes. We recommend that model choice is informed using available and relevant prior knowledge, model transparency, graphically comparing survival curves alongside observed data to aid consideration of the reliability of the survival estimates, and consideration of how the treatment effect estimates can be incorporated within a decision model.

## 1 Background

Evidence synthesis is a well-established component of health technology assessment (HTA), applied to quantitatively combine the data from multiple trials in order to obtain an overall pooled estimate of clinical effectiveness. This in turn may be used to inform an associated economic evaluation. Such economic evaluations form the basis of National Institute for Health and Care Excellence (NICE) guidance in the United Kingdom.^1^ For comparisons between two healthcare interventions, it is common practice to apply pairwise meta-analysis (MA) methods to obtain pooled effectiveness estimates. However, where more than two interventions are of interest, network MA (NMA)^2^ (also known as *multiple treatment comparisons*^3^ and *mixed treatment comparisons*^4,5^) is required. This type of analysis extends pairwise MA to allow the simultaneous estimation of comparative effectiveness of multiple interventions using an evidence base of trials that individually may not compare all intervention options, but form a connected network of comparisons. NMA can reduce uncertainty around key cost-effectiveness measures compared with pairwise MA^6^ and also allows interventions to be ranked to establish the most effective intervention(s).

NMA can be performed under both the frequentist and Bayesian frameworks but have traditionally been performed under the Bayesian framework using WinBUGS.^7–9^ Under either framework, models can be fitted in a one- or two-stage process using individual participant data (IPD) or aggregated data. IPD is generally considered the gold standard for MA (and NMA) but is particularly advantageous for time-to-event (TTE) outcomes as it allows modelling of time-dependent effects.^10–18^ In a two-stage process an estimate of the treatment effect and its precision are calculated for each trial, if IPD is available, or extracted from trial publications if aggregate data is used. A fixed or random effect model is then used to synthesise the treatment effect estimates across trials.^14,19–23,16,17^ In a one-stage process, this all happens within a single statistical model.^14,19–23,16,17^ When fitting the same model with the same assumptions it has shown that under the frequentist framework the one and two-stage processes are mathematically equivalent.^19,21,22^ Under the Bayesian framework, different prior distributions will be required for one- and two-stage models which may result in a small variation between the one- and two-stage models. However, whichever framework is used, the advantage of the one-stage process is that a wider variety of models can be fitted.^19,20^ The synthesis of TTE outcomes regarding treatment effects is typically based on a comparison of hazard ratios (HR) derived from the Cox proportional hazards (PH) model.^24^ The Cox PH model is semi-parametric, making no assumption about the baseline hazard rate but assuming that the hazard ratio is proportional over time.^24^ Although the HR may be a useful statistic in the context of statistical inference within an individual trial, where it can be taken as representing an average of the treatment effect across the trial period,^25,26^ a single HR may not be sufficient for a wider evidence synthesis.^26^ This may occur when the form of the TTE curves vary markedly between treatment arms violating the PH assumption.^27–29,26^ For example, there is some evidence that a fraction of patients experienced markedly prolonged TTE when treated with immuno-oncologic therapy compared to conventional chemotherapy.^30,31,26,32–34^ In this case, the HR would be small initially but increase over time. If the HR does vary materially during the trial period, the overall estimates of the HR may be confounded by differences in trial duration and decisions based on them misleading.^35,26,36^ In addition, extrapolating beyond the trial period, the predicted TTE curves underpinning cost-effectiveness models, and thus decision making, will not be reliable.^27^ Therefore alternative methods for NMA of TTE outcomes are needed.

As an alternative to synthesising constant HRs across trials, Ouwens et al.^37^ first proposed synthesising multiple parameters from parametric survival curves. They modelled the hazard function for each trial using the two-parameter Weibull distribution and extended the model to the NMA setting showing that the transitivity assumption holds when synthesising the difference in both the shape and scale parameters.^37^ Although they considered the Weibull distribution the same principle can be applied to other distributions such as the Gompertz, log-logistic and log-normal distributions.^37^ Fractional polynomials are continuous functions which provide a flexible alternative to regular polynomial functions.^38^

Fractional polynomials were proposed as a method for overcoming the limited shapes available with low order polynomials and avoiding problems with poor fit at extreme values with higher order polynomials.^38^ The power terms for fractional polynomials are restricted to the set −2,−1,−0.5,0,0.5,1,2,3 which was selected to ensure that conventional polynomials are a subset of fractional polynomials.^38^ Typically fractional polynomials are chosen to have either one power (known as a first-order model) or two powers (known as a second-order model).^38^ In a fractional polynomial NMA model, the log hazard rate for each trial is modelled using a fractional polynomial allowing the hazard rate to be related to time via a complex linear function determined by the choice of power(s).^27,39^ The fractional polynomial NMA model results in a multi-dimensional treatment effect removing the PH restriction and the transitivity assumption holds when the difference in the fractional polynomial parameters is synthesised.^27,39^ Fractional polynomials can result in a wide variety of shapes for the hazard function including constant, increasing, decreasing or bathtub shaped hazards.^27,39^ In contrast to the piecewise exponential model, a fractional polynomial model applies constraints between time periods to ensure a smooth estimate of the baseline hazard function.^40^ Multi-dimensional models such as the parametric models proposed by Ouwens et al.^37^ and the fractional polynomial models proposed by Jansen^27^ can be extended further to include study-level covariates and treatment-covariate interactions acting across the multi-dimensional treatment effects to adjust for confounding bias resulting from systematic differences in treatment modifiers across comparisons.^39^

Another approach for synthesising TTE outcomes in the presence of non-PH are piecewise exponential models. Piecewise exponential models assume a constant hazard rate within each time period but can allow the hazard rate to vary between a set of discrete time periods.^41^ Piecewise exponential models offer a flexible approach to modelling survival data but they can lack biological plausibility due to the assumption of an instantaneous change in the hazard rate between time intervals. Latimer considered piecewise exponential models to be ‘an under-used modelling approach in HTA’ but also acknowledged that they may not be the best approach for extrapolating survival curves beyond the observed data.^42^ Crowther et al.^43^ showed that piecewise exponential models can be fitted using Poisson generalised linear survival models in a one-stage MA using IPD. These models can be implemented with either fixed or random treatment effects and with the baseline hazard stratified by trial. The Poisson approach would obtain an identical estimate of the treatment effect to that from a Cox model if the follow-up time was split at each unique event time.^43^

Standard parametric models can restrict the shape of hazard functions and may not adequately capture the shape of hazard functions seen in applied studies.^44^ Flexible parametric models use restricted cubic splines to model complex hazard functions.^44^ Restricted cubic splines are functions of time which can capture complex shapes and enable more realistic modelling of hazard functions.^45^ A restricted cubic spline is a series of polynomial functions. At the joining points, known as knots, the polynomial functions are forced to join with continuous first and second derivatives resulting in a smooth function which is linear beyond the boundary knots.^45,46^ The complexity and flexibility of the restricted cubic spline is governed by the number and location of knots.^46,45,47,48^ The Royston-Parmar model is a flexible parametric model which uses a restricted cubic spline to model the baseline log cumulative hazard rate for each trial.^46,48^ The model can be specified as a PH model or a proportional odds model.^45^ In comparison to the Cox model, which requires each individual’s data to be repeated for each risk set they belong to, the Royston-Parmar model provides a flexible and computationally practical alternative which makes full use of the IPD available. The Royston-Parmar has been implemented in the NMA setting including extensions to allow for non-PH, covariates and treatment-covariate interactions.^48^ The PH assumption can be relaxed through the inclusion of treatment-ln(time) interactions.^48^

Another alternative to PH models for modelling TTE outcomes are accelerated failure time (AFT) models.^49,50^ In an AFT model, the treatment acts as a multiplier on the time at which a given survival percentile is reached. Several of the standard parametric models are AFT models including the Weibull, log-logistic, log-normal and generalised gamma distributions. In some cases, using a parametric approach can restrict the shape of the baseline survival curve. However, the generalised gamma distribution can accommodate increasing, decreasing, bathtub and arc-shaped hazards^51^ and nests within it the exponential, Weibull, gamma and log-normal models and can approximate the log-logistic distribution.^52^ Therefore, it provides a flexible alternative to the Cox PH model. An advantage of this approach is that there is no need to specify in advance which distribution you expect your data to follow and it allows each trial to follow a different distribution (if appropriate). Within this framework, an accelerated failure time parameterisation of the treatment can be explored. To date, this approach has been used in pairwise meta-analysis^53,54^ but its application to network meta-analysis has been limited.

Restricted mean survival time (RMST) has been proposed as an alternative outcome measure to the HR in trials reporting TTE outcomes when there is evidence of non-PH.^25^ The RMST is the mean survival time up to a pre-specified time point t* corresponding to the area under the survival curve from 0 to t* .^25^ When the outcome is overall survival the RMST can be interpreted as the average life expectancy of a patient over the next t* years.^25,55^ There are several methods for estimating the survival function including using the Kaplan-Meier estimate of the survival function.^25,55–57^ In the MA setting, synthesising the between-arm difference in the RMST is a way of avoiding the PH assumption thus allowing the treatment effect to vary over time.^55,57^ The use of RMST in NMA is still in its infancy. However, a recent paper comparing RMST with HRs for NMA of nasopharyngeal carcinomas found that, in some cases, trials exhibiting evidence of non-PH impacted the direction of the treatment effect in the NMA.^58^

Despite the increasing awareness around the presence of non-PH two recent reviews of HTA guidelines and HTA reports found that outcome measures allowing for non-PH are rarely reported. A review of methodological guidelines published since 2014 by 10 HTA agencies and 23 oncology HTA reports approved by the US Food and Drug Administration and the European Medicines Agency since 2014 found that testing for non-PH is not widely incorporated into HTA except by NICE and RMST is used infrequently but most commonly by agencies that focus on cost-effectiveness.^26^ A review of NICE technology appraisals, NICE guidelines and National Institute for Health Research HTA reports published between April 2018 and March 2019 identified 26 articles reporting at least one time-to-event outcome. Only four articles reported outcome measures allowing for non-PH (fractional polynomial parameters or time varying HRs).^59^

The aim of this paper is to compare and contrast the RMST, generalised gamma, piecewise exponential, fractional polynomial and Royston-Parmar models for NMA of TTE outcomes where non-PH and differing lengths of trial follow-up are present through application to a melanoma network to provide guidance on the key decisions for selecting between these methods. We start by introducing a network of melanoma trials before describing the five approaches outlined above for conducting NMA with TTE outcomes. We then consider the key criteria for choosing between different modelling approaches for NMA of TTE outcomes and present the results of applying the five methods for NMA of TTE outcomes to the melanoma network. We finish with a discussion.

## 2 Example: Melanoma network

Our example comes from a recent systematic review (SR) and NMA of therapies for previously untreated advanced BRAF-mutated melanoma.^60^ We chose this example as it represents a clinical area in which non-PH is commonly encountered and the structure of the network, many treatment options with limited direct head-to-head evidence, represents a commonly encountered situation in HTA appraisals. The SR identified 23 eligible articles reporting on thirteen phase II and phase III randomised controlled trials (RCTs). Eligible RCTs enrolled treatment-naive adult patients with unresectable lymph node metastasis and included at least one intervention which was a targeted (BRAF or MEK) or immune checkpoint (CTLA-4 or PD-1) inhibitor. Full details on the search strategy and inclusion and exclusion criteria are published elsewhere.^61,60^ For each trial, we identified the most recently published article including a Kaplan-Meier curve of overall survival.^62–74^ We used WebPlotDigitizer^75^ to extract data points from the Kaplan-Meier curve for each trial arm. The Guyot algorithm^76^ was used to re-create IPD for each trial arm. The hazard ratios and the shape of the Kaplan-Meier survival plots from our re-constructed IPD were compared back to the trial publications to ensure a level of accuracy in the re-construction process. This process is sufficient for demonstrating the methodology in this paper. However, it is widely accepted that re-constructed IPD should not be used for clinical inference. Where modelling approaches required data in an aggregated format we aggregated the re-created IPD rather than extracting aggregated data from the trial publications. This ensured all models were fitted to the same data allowing a fair comparison between models.

**Figure 1.**
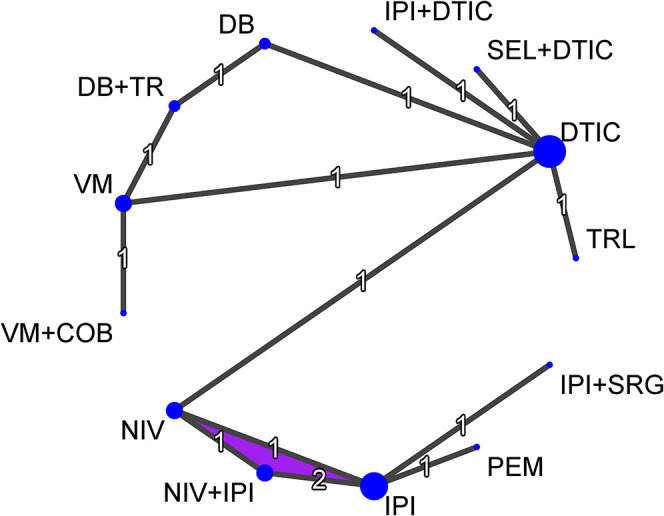
Melanoma network diagram. Node size is proportional to the number of studies including each treatment and line thickness is proportional to the number of studies involved in each direct comparison. The numbers on each line represent the number of studies involved in each direct comparison. The purple region indicates a multi-arm trial. COB = Cobimetinib, DB = Dabrafenib, DTIC = Dacarbazine, IPI = Ipilimumab, NIV = Nivolumab, PEM = Pembrolizumab, SEL = Selumetinib, SRG = Sargramostin, TR = Trametinib, TRL = Tremelimumab, VM = Vemurafenib.

In this paper, the melanoma network consists of 3913 overall survival events from 6378 patients. The network includes 13 RCTs and 13 treatment options: dacarbazine, tremelimumab, ipilimumab, dabrafenib, vemurafenib, nivolumab, pembrolizumab, ipilimumab plus dacarbazine, dabrafenib plus trametinib, vemurafenib plus cobimetinib, nivolumab plus ipilimumab, selumetinib plus dacarbazine and ipilimumab plus sargramostin. The network structure is presented in Figure 1. Based on the network structure, in all our models, we consider dacarbazine to be the reference treatment across the network. Trial arm size ranged from 45 to 556 patients. Median follow-up time across trial arms ranged from 12.3 to 63.7 months. Key characteristics of the extracted IPD for each trial are presented in Online Appendix A. Kaplan-Meier plots of survival over time from each trial are presented in Online Appendix B. A snapshot of the IPD for this network is provided in Online Table C1 (Online Appendix C). The full IPD for this network is available at: https://github.com/SCFreeman/Melanoma_NMA.

Based on the network structure in Figure 1, where all of the treatment comparisons for which direct evidence exists, except one, are informed by a single trial we fitted fixed treatment effect NMA models only.

Each trial was assessed individually for evidence of PH. The Nelson-Aalen estimate of the log cumulative hazard was plotted against log time for all trials as a visual aid and a chi-squared test based on the Schoenfeld residuals was conducted. Based on intersecting treatment lines on the plots of log cumulative hazard versus log time, ten trials showed evidence of non-PH (Online Appendix D). Three trials had statistically significant *p*-values (*p*<0.05) based on the chi-squared test of the Schoenfeld residuals. As the models we consider in this paper can account for non-PH we did not conduct sensitivity analyses excluding these trials.

## 3 Methods

In this section we start by reviewing the commonly used Cox PH model^24^ before considering five alternative approaches to modelling TTE data for synthesising treatment effects: restricted mean survival time,^25,55^ the generalised gamma model,^77^ the piecewise exponential model,^41,43^ fractional polynomial models^27^ and the Royston-Parmar flexible parametric model.^45,48^ All R and WinBUGS code for implementing these models is available at: https://github.com/SCFreeman/Melanoma_NMA.

Each trial within a network meta-analysis has a baseline treatment which we denote b . In contrast-based models, within each trial each treatment q is compared to the baseline treatment b . When fitting a NMA model we also have to choose a reference treatment which we denote q=1 . Treatment effects from NMA models are reported compared to the reference treatment, dacarbazine.

### 3.1 Cox PH model

The Cox PH model was fitted using a two-stage approach. In the first stage a Cox PH model was fitted individually to each trial j to obtain an estimate of the log HR for the treatment effect and its corresponding standard error. The Cox PH model is a semi-parametric model in which the hazard rate is assumed to be proportional over time and for a trial j takes the form

$$h_{\;j,bq}(t)=h_{0j,bq}(t)\exp(\alpha_{\;j,bq}x_{ij})$$

where $h_{\;j,bq}(t)$ is the hazard function for treatment q compared to the baseline treatment b in trial j , $h_{0j,bq}(t)$ is the baseline hazard function for trial j , $x_{ij}$ is the treatment indicator variable for patient i from trial j taking the value 0 if patient i receives the baseline treatment b and the value 1 if patient i receives treatment q , and $\alpha_{\;j,bq}$ the treatment effect, in this case the HR for a patient receiving treatment q compared to the baseline treatment b in trial j . This stage was implemented using the *coxph* function from the survival package^78^ in R version 3.6.1.^79^ In the second stage, we synthesised the treatment effect estimate (i.e. the log HR), ${\hat{\alpha }}_{\;j,bq}$ , and an estimate of its variability, $Var({\hat{\alpha }}_{\;j,bq})$ , for the baseline treatment b compared to treatment q in trial j , within a standard fixed effect NMA model. The fixed effect model assumes that ${\hat{\alpha }}_{\;j,bq}$ are all estimates of the same underlying treatment effect, $\alpha_{bq}$ :

$${\hat{\alpha }}_{\;j,bq}∼N(\alpha_{bq},Var({\hat{\alpha }}_{\;j,bq}))$$

The treatment effect parameters ${\hat{\alpha }}_{\;j,bq}$ were fitted with non-informative normal prior distributions with mean 0 and precision 0.0001.

### 3.2 Restricted mean survival time

RMST, $\psi^{*}$ , is defined as the area under the survival curve S(t) up to the time point $t^{*}$ . We synthesised RMST across trials using a two-stage process. In the first stage, we used the Kaplan-Meier estimate of survival time, $\hat{S}(t)$ , to calculate the RMST for each treatment q in trial j from 0 to 18 months:

$$\psi_{\;jq}^{*}=\int_{0}^{18}\hat{S}(t)dt$$

The choice of $t^{*}$ must be equal to or less than the minimum value of the largest observed survival time across all trials. For the melanoma network this restricted our choice to $t^{*}$ to 18 months. This first stage was conducted using the *rmst2* command from the survRM2 package in R.^80^ In the second stage, the estimates of RMST and the standard errors from each trial arm were synthesised using a standard fixed effect NMA model to estimate the difference in RMST for each treatment q compared to the reference treatment, denoted by q=1 . The fixed effect model assumes that ${\hat{\psi }}_{\;jq}^{*}$ are all estimates of the same underlying treatment effect, $\psi_{q}$ :

$$\begin{array}{rl} {\hat{\psi }}_{\;jq}^{*} & ∼N(\psi_{\;jq},Var({\hat{\psi }}_{\;jq}^{*}))\\ {\hat{\psi }}_{\;jq}^{*} & =\left\{ \begin{array}{cc} \mu_{j} & \text{if }q=1\\ \mu_{j}+\psi_{q} & \text{if }q>1 \end{array}\right. \end{array}$$

where $\mu_{j}$ is the trial-specific baseline effect and $\psi_{q}$ represents the treatment effect for treatment q compared to the network reference treatment q=1 . $\psi_{q}$ was fitted with a non-informative normal prior distribution with mean 0 and precision 0.0001.

### 3.3 Generalised gamma model

The generalised gamma model was fitted using a two-stage process. In the first stage, each trial was analysed separately using the generalised gamma model to obtain estimates of the log hazard ratio for the treatment effect and the corresponding standard error. This stage was implemented using the *flexsurv* package^81^ in R version 3.6.0^79^ which fits the three-parameter parameterisation originating from Prentice.^77^ If t∼Gamma(γ,1) then the probability density function for the three-parameter generalised gamma model is

$$f(t|\alpha ,\eta ,Q)=\left\{ \begin{array}{cc} \frac{\gamma^{\gamma }}{\eta t\gamma^{-2}\Gamma (\gamma )}\text{exp}(z\gamma^{-2}-u) & \text{if }\;Q\neq 0\\ \frac{1}{\eta t(2\pi {)}^{-2}}\text{exp}(\frac{-z^{2}}{2}) & \text{if }\;Q=0 \end{array}\right.$$

where $\gamma =|Q{|}^{-2}$ , u=γexp(|Q|z) and $z=\text{sign}(Q)\frac{\text{log}(t)-\alpha }{\eta }$ . Here, t is survival time, α is the location parameter, η>0 is the scale parameter and Q is the shape parameter. We fitted models in which the treatment effect was dependent on the location parameter only. The model is implemented by parameterising $\text{log}(t_{ij})=x_{ij}\alpha$ where $x_{ij}$ is the treatment indicator variable for patient i from trial j taking the value 0 if patient i receives the baseline treatment b and the value 1 if patient i receives treatment q and α is the regression coefficient representing the treatment effect for treatment q compared to the baseline treatment b .

In the second stage, we synthesised the treatment effect estimate, ${\hat{\alpha }}_{\;j,bq}$ , and an estimate of its variability, $Var({\hat{\alpha }}_{\;j,bq})$ , for the baseline treatment b compared to treatment q in trial j , within a standard fixed effect NMA model. The fixed effect model assumes that ${\hat{\alpha }}_{\;j,bq}$ are all estimates of the same underlying treatment effect, $\alpha_{bq}$ :

$${\hat{\alpha }}_{\;j,bq}∼N(\alpha_{bq},Var({\hat{\alpha }}_{\;j,bq}))$$

The location parameters were given non-informative normal prior distributions with mean 0 and precision 0.0001.

### 3.4 Piecewise exponential model

We used the Poisson approach of Crowther et al.^43^ to fit piecewise exponential models. This approach involves splitting the overall time horizon into intervals and fitting an exponential model in each interval. This allows for the sharing of information on the hazard ratio across time intervals.^41^To do this the IPD were aggregated over time interval, treatment and trial so that for each time interval, for each treatment, for each trial, we had the number of patients at risk, the number of events and the sum of the time at risk for all patients at risk during the time interval. Time intervals can be of equal or differing lengths but must be common across all trials in the network. As described in Online Appendix C, we chose to split the data into three intervals: 0–6 months, 6–12 months and >12 months. We applied the Poisson approach in the NMA setting with fixed treatment effects and baseline hazard stratified by trial. To obtain the correct form of the likelihood for a piecewise exponential model, let $e_{ijk}$ be an event indicator representing a Poisson process for each patient i in each trial j during each time interval k with $\lambda_{ijk}$ representing the event rate for each patient in each trial during each time interval.^43^ To allow for non-PH in the treatment effects we dichotomise follow-up time at time $t_{w}$ and introduce a variable $w_{k}$ which takes the value 0 if $t<t_{w}$ and 1 if $t\geq t_{w}$ . The parameter $\rho_{q}$ represents the change in log hazard ratio when $t\geq t_{w}$ compared to when $t<t_{w}$ for treatment q . In a network of q+1 treatments the fixed effect model is

$$\begin{array}{rl} e_{ijk} & ∼\text{Poisson}(\lambda_{ijk})\\ \text{ln}(\lambda_{ijk}) & =\alpha_{1}{\text{trt1}}_{\;jk}+\ldots +\alpha_{q}{\text{trtq}}_{\;jk}+\beta_{\;jk}\\ & \;+\rho_{1}{\text{trt1}}_{\;jk}w_{k}+\ldots +\rho_{q}{\text{trtq}}_{\;jk}w_{k}+\text{ln}(y_{ijk}) \end{array}$$

where ${\text{trt1}}_{\;jk},\ldots ,{\text{trtq}}_{\;jk}$ are treatment contrast variables (described in more detail in Online Appendix C), $\alpha_{1},\ldots ,\alpha_{q}$ the treatment effects for treatments 1,…,q compared to the network reference treatment, $\beta_{\;jk}$ the baseline hazard for trial j during time interval k and $y_{ijk}$ is the observed survival time for all patients in trial j and time interval k , included as a log offset.

This model can be extended further to include more than one cut point. With the melanoma data split into three time intervals (0–6 months, 6–12 months and >12 months) the natural place for a cut point would be 6 or 12 months. We considered one cut point placed at 6 months, one cut point placed at 12 months and two cut points placed at 6 and 12 months. A non-informative normal prior distribution was used for β with mean 0 and precision 0.0001. $\rho_{q}$ was fitted with a normal prior distribution with the mean 0 and precision 0.01.

### 3.5 Fractional polynomial models

To fit fractional polynomial models, we used the same time intervals as for the piecewise exponential models, aggregating the IPD into three intervals: 0–6 months, 6–12 months and >12 months (see Online Appendix C for full details). The fractional polynomial framework offers the potential for fitting eight first-order models, each taking one of the powers from the set: −2,−1,−0.5,0,0.5,1,2,3 and 36 second-order models, taking any combination of two powers from the same set. We fitted a fixed effect NMA using the first and second order fractional polynomial NMA models proposed by Jansen.^27,82^ Let j index trial and q treatment arm then the second-order fixed treatment effect fractional polynomial NMA model at time point t is

$$\begin{array}{rl} \ln(h_{\;jqt}) & =\left\{ \begin{array}{cc} \alpha_{0,jq}+\alpha_{1,jq}t^{\;p_{1}}+\alpha_{2,jq}t^{\;p_{2}} & \;p_{1}\neq p_{2}\\ \alpha_{0,jq}+\alpha_{1,jq}t^{\;p}+\alpha_{2,jq}t^{\;p}\ln\;(t) & \;p=p_{1}=p_{2} \end{array}\right.\\ \left(\begin{array}{c} \alpha_{0,jq}\\ \alpha_{1,jq}\\ \alpha_{2,jq} \end{array}\right) & =\left(\begin{array}{c} \beta_{0,j}\\ \beta_{1,j}\\ \beta_{2,j} \end{array}\right)+\left(\begin{array}{c} d_{0,jq}-d_{0,j1}\\ d_{1,jq}-d_{1,j1}\\ d_{2,jq}-d_{2,j1} \end{array}\right) \end{array}$$

where $h_{\;jqt}$ is the hazard for treatment arm q in trial j at time point t and the powers $p_{1}$ and $p_{2}$ , in this case, are chosen from the set: −2,−1,−0.5,0,0.5,1,2,3 with $t^{0}=\ln\;(t)$ . β are parameters which represent alpha for the trial-specific baseline treatment and d are fixed effects for the trial-specific differences in $\alpha_{0}$ , $\alpha_{1}$ and $\alpha_{2}$ . The first-order fixed treatment effect model is obtained by omitting the $\alpha_{2}$ terms. Here, $\alpha_{0}$ represents a scale parameter and $\alpha_{1}$ a shape parameter of the log hazard function. The inclusion of a second shape parameter ( $\alpha_{2}$ ) makes changes in the direction of the hazard function a possibility.^82^ Therefore, the fractional polynomial approach can accommodate a wide range of baseline hazards. Consistency of treatment effects in this model is through the α terms.^27,82^ We fitted each of the first-order fixed effect models and considered second-order fixed effect models incorporating the power identified as the best fitting first-order fixed effect model. β and d were fitted with non-informative multivariate normal prior distributions with mean, for the first-order models, $\left(\begin{array}{c} 0\\ 0 \end{array}\right)$ and precision $\left(\begin{array}{cc} 0.0001 & 0\\ 0 & 0.0001 \end{array}\right)$ .

### 3.6 Royston-Parmar flexible parametric model

For each trial j , the log cumulative hazard, $H_{j}$ , is modelled individually with its own restricted cubic spline, see Online Appendix C for details on the location of knots. Non-PH can be considered by including interactions between treatment and ln(time). For patient i in trial j in a network of q+1 treatments the fixed treatment effect NMA model allowing for non-PH takes the form

$$\begin{array}{rl} \ln\;\{H_{j}(t|{\mathrm{trtq}}_{i})\} & =s_{j}(\ln\;(t_{i}))+\alpha_{1}{\text{trt1}}_{i}+\ldots +\alpha_{q}{\text{trtq}}_{i}\\ & \;+\alpha_{\left(q+1\right)}{\text{trt1}}_{i}\left(\ln\;(t_{i})\right)+\ldots +\alpha_{\left(2q\right)}{\text{trtq}}_{i}\left(\ln\;(t_{i})\right) \end{array}$$

where ${\text{trt1}}_{i},\ldots ,{\text{trtq}}_{i}$ are treatment contrast variables, $\alpha_{1},\ldots ,\alpha_{\left(2q\right)}$ the treatment effects for treatments 1,…,q compared to the network reference treatment and $s_{j}(\ln\;(t_{i}))$ the restricted cubic spline for trial j . Some care is needed in defining the treatment contrast variables to ensure they are in the right direction and the consistency equations hold, see Online Appendix C for details. Parameters representing the spline functions for the baseline log cumulative hazard function and the treatment effect parameters α were fitted with non-informative normal prior distributions with mean 0 and precision 0.0001.

### 3.7 Fitting models in WinBUGS

All models were fitted in WinBUGS version 1.4.3^7^. All models were run with at least 10,000 burn in, 10,000 iterations and with three sets of initial values. Where necessary to ensure convergence larger number of burn in and iterations were used. Convergence was checked through visual inspection of density plots and history plots. The deviance information criteria (DIC) statistic^83,84^ was reported as a statistical measure of model fit. DIC is a relative measure of model fit and can therefore only be used to compare models fitted to the same dataset within a model family (e.g. fractional polynomial model with p=0 compared to fractional polynomial model with p=0.5 ). We consider reductions in DIC of three or more to indicate a better fitting model.

In this illustrative example, all models were run with non-informative prior distributions. However, where prior knowledge is available it can be incorporated within the prior distributions^85,86^.

### 3.8 Model comparison

The aim of this paper is to compare and contrast different modelling approaches for NMA of TTE outcomes where non-PH and differing lengths of trial follow-up are present through application to the melanoma network. Therefore, we do not make formal comparisons of the performance of these models. However, to assist in illustrating the different methods we assess the consistency of the survival estimates across the different modelling approaches in a number of ways. Firstly, we compare the appearance of the survival curves by considering whether treatments have the same pattern of survival across the different models and where any differences may lie. Secondly, we calculated the probability of each treatment obtaining each rank from 1 to 13. In the two-stage Cox PH model and RMST model, we ranked the treatments based on the treatment parameter estimates from the second stage. In the generalised gamma models, we ranked the treatments based on the location parameters from the second stage. In the piecewise exponential, fractional polynomial and Royston-Parmar models, we ranked the treatments based on survival at 60 months. Finally, to quantify the estimated gain in survival for each treatment under the different modelling approaches, we calculated the improvement in the area under the survival curve at 60 months compared to the network reference treatment of dacarbazine.

### 3.9 Estimating survival

Assessing clinical effectiveness is the first stage of the HTA process. Estimates of clinical effectiveness are often used to inform economic decision models. In a decision model, relative treatment effects, estimated from the NMA, are combined with a baseline survival curve, which represents the absolute natural history for the reference treatment, to obtain estimates of absolute survival over time for the treatments under investigation.^87,88^ Therefore, it is important that the reference survival curve represents the target clinical population.

The reference survival curve should be as specific to the target clinical population as possible. A popular approach is to synthesise the reference treatment arms from all trials including the reference treatment. However, this approach has several strong assumptions and it is important to consider whether all the trials used to inform the relative effects can be considered equally representative of the target clinical population.^87^ By synthesising multiple trials we must either assume that the target clinical population corresponds to one of our trials but we are not sure which one, and in this case, we should use the predictive distribution from a random effects analysis, or we must assume that the future clinical population is a random mixture of the patients from all the trials (despite the fact there are systematic differences between the patients randomised in each of the trials), and in this case we should use the mean of the random effect and its uncertainty. However, it may be more appropriate for the reference survival curve to come from one trial in which the population is representative of the target clinical population.^87,88^ Alternatively, if no trial is felt to be representative of the target clinical population then we may incorporate data from an external source into the economic decision model.^87,88^ For a full discussion on the options available see Welton et al.^88^ and for details on fitting baseline natural history models see Dias et al.^87^ To assess heterogeneity between the dacarbazine arms in the network we plotted the Kaplan-Meier survival estimate from each trial reporting a dacarbazine arm in Appendix E. With the exception of BREAK3^62^, the remaining five trials^63,64,72–74^ were homogeneous in their pattern of overall survival. Therefore, we chose a single representative trial as our reference survival curve. We chose the dacarbazine arm of the CheckMate 066^64^ trial as our reference survival curve because this was the most recently published overall survival data.

## 4 Criteria for selecting models

We have introduced five alternative options to the Cox model for conducting an NMA of TTE outcomes. However, selecting which model to use, particularly when the results of the NMA effect decision making, is not straight forward. In this section we discuss factors beyond statistical measures of model fit which should be considered when selecting the ‘best’ model.

When considering which model to fit, ideally we want to choose a model which fits our data well and provides reliable extrapolations. When considering the fit to data it is important to consider the parameterisation of the model. This is not just which modelling approach to choose but if, for example, following the fractional polynomial or piecewise exponential approaches then we also need to consider how many models are tested before selecting the ‘best’ fit as this effectively adds a number of what we term ‘hidden parameters’. Another important issue is the transparency of the modelling approaches. We consider ‘transparency’ by stating for each model: the basis of the extrapolation of relative treatment effects, extrapolation of baseline risk, the underlying consistency assumption and the fit to individual trials. Here, we define consistency as the agreement between the direct and indirect evidence within the network. We believe it is transparency which facilitates the application of prior knowledge.

Another factor which can be beneficial is easily interpretable parameters. The advantage to having interpretable parameters is that we can check face validity, source validity and external validity. However, even if the parameters themselves are not easy to interpret we are often able to use them to make predictions which may be more important than the parameters themselves. Furthermore, if the aim is to include a measure of clinical effectiveness in a decision model then we should also consider whether this is possible. In Table 1, we comment specifically on how these factors effect the five modelling approaches we considered above.

**Table 1.** Criteria for selecting models.

	Restricted mean survival time	Generalised gamma	Piecewise exponential	Fractional polynomial	Royston-Parmar
Underlying consistency assumption*	Treatment effects expressed as a difference in restricted mean survival remain constant as absolute survival varies.	Treatment effects on location only: treatment effects expressed as acceleration factor remain constant as absolute survival varies. Treatment effects on shape or scale: no simple description of consistency assumption.	Treatment effects within time periods expressed as hazard ratios are constant as absolute survival varies.	There is not a simple description of consistency assumption	Treatment effects expressed as hazard ratios are constant as absolute survival varies.
Number of Parameters used to describe treatment effects (determines risk of over-fitting; *nTx* = number of treatments)	nTx−1	Treatment effects on location only: 3 + (*nTx* − 1). Treatment effects on shape or scale: 3 + 2.( nTx−1 ). Treatment effects on shape and scale: 3 + 3.( nTx−1 ).	Number of time intervals multiplied by number of treatments. Can be reduced by sharing information across time points.	First-order model: *nTx*. Second-order model: 2.*nTx*.	2.( nTx−1 )
Structural choices (effectively increases number of parameters)	Choice of time point at which to evaluate survival.	Choice of whether to place treatment effect on scale or shape parameters.	Requires choice of time intervals and placement of cut points.	Requires choice of powers.	Requires users to define the number and location of knots.
Extrapolation of relative treatment effect	Treatment effects are not extrapolated.	Treatment effect assumed constant on accelerated failure time scale.	Treatment effect assumed constant on hazard scale from final time interval.	Complex function of parameter estimates.	Treatment effect assumed constant on hazard scale beyond boundary knots.
Extrapolation of reference treatment survival for decision-model	Extrapolation beyond observed period requires a choice of parametric model.	Complex function of estimated parameters.	Hazard is assumed constant from final interval.	Complex function of parameter estimates.	Hazard constant beyond boundary knots.
Comparison of fit to individual trials	Estimated treatment effects in terms of RMST can be compared to individual trial results.	Treatment effects on location only: Estimated treatment effects in terms of acceleration factors can be compared to individual trial results. Treatment effects on shape or scale: not readily comparable to individual trial results.	Not readily comparable to individual trial results.	Not readily comparable to individual trial results.	Estimated treatment effects in terms of hazard ratios can be compared to individual trial results.
Interpretability & ability to apply external knowledge (including tapering of treatment effects)	Treatment effect parameters readily interpretable, can be compared to external evidence.	Treatment effect parameters readily interpretable, can be compared to external evidence.	Time interval selection can be based on prior belief. Treatment effect parameters readily interpretable, can be compared to external evidence.	Treatment effect parameters not readily interpretable, cannot be easily compared to external evidence.	Treatment effect parameters readily interpretable, can be compared to external evidence.

*Consistency is defined as the agreement between direct and indirect evidences.

## 5 Results

In this section, we report the results of fitting the Cox PH, RMST, generalised gamma, piecewise exponential, fractional polynomial and Royston-Parmar models to the melanoma network focusing on the observed fit of the models. We do not make formal comparisons of the performance of these models but to assist in illustrating the different methods we assess the consistency of the survival estimates across the different modelling approaches, as described above. Based on the network structure in Figure 1, where all of the treatment comparisons for which direct evidence exists, except one, are informed by a single trial we fitted fixed treatment effect models only. Parameter estimates for all models can be found in Online Tables F1 to F6 (Online Appendix F).

### 5.1 Cox PH model

**Figure 2.**
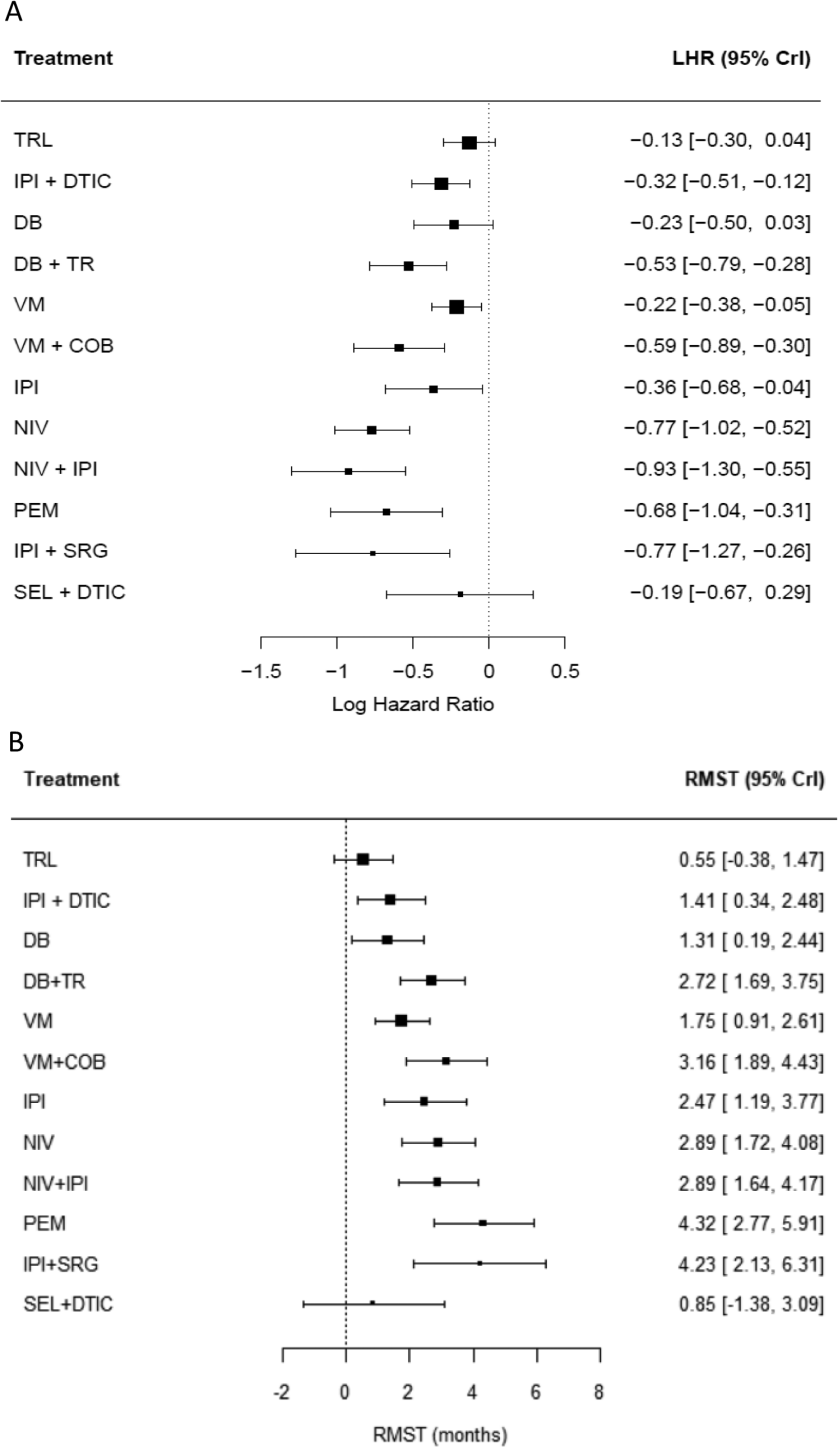
Forest plot of (A) log hazard ratios; and (B) restricted mean survival time at 18 months. All treatments are compared to dacarbazine. COB = Cobimetinib, DB = Dabrafenib, DTIC = Dacarbazine, IPI = Ipilimumab, NIV = Nivolumab, PEM = Pembrolizumab, SEL = Selumetinib, SRG = Sargramostin, TR = Trametinib, TRL = Tremelimumab, VM = Vemurafenib.

**Figure 3.**
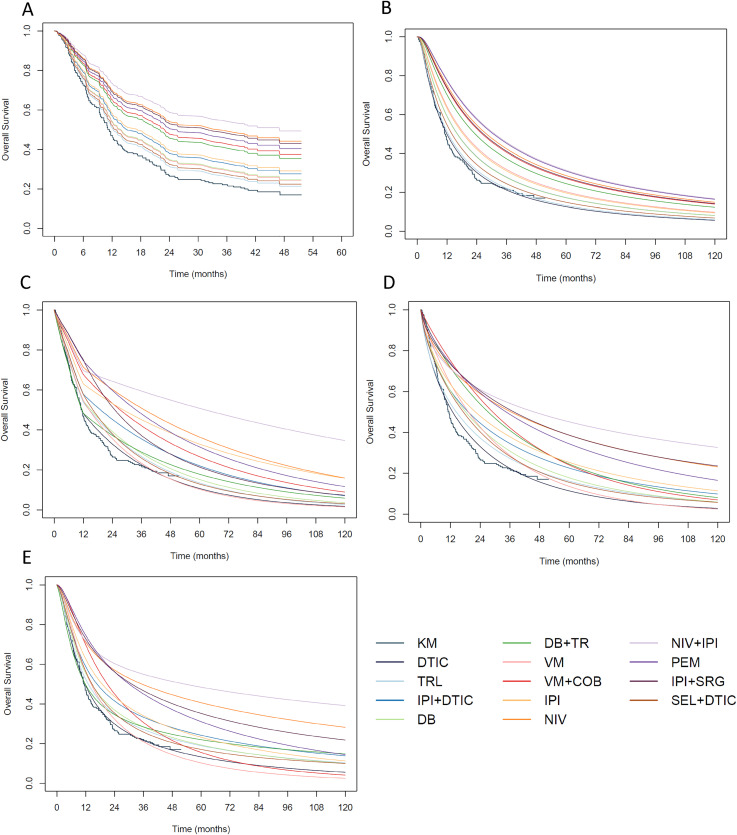
Survival curves from (A) the fixed effect hazard ratio NMA model; (B) the fixed effect generalised gamma model; (C) the fixed effect piecewise Poisson model with cut point at 12 months; (D) the first-order fixed effect fractional polynomial model with p=0; and (E) the fixed effect Royston-Parmar model with treatment-ln(time) interactions. COB = Cobimetinib, DB = Dabrafenib, DTIC = Dacarbazine, IPI = Ipilimumab, NIV = Nivolumab, PEM = Pembrolizumab, SEL = Selumetinib, SRG = Sargramostin, TR = Trametinib, TRL = Tremelimumab, VM = Vemurafenib.

The hazard ratio and 95% confidence intervals from a Cox PH model fitted to each trial are reported in Online Appendix A. The log hazard ratios and 95% credible intervals for each treatment compared to dacarbazine from the fixed effect NMA model are presented in Figure 2(a) and the corresponding survival curves in Figure 3(a). Based on Figure 2(a), the treatment with the greatest improvement in survival is nivolumab plus ipilimumab (LHR =−0.93 , 95% CrI: −1.30,−0.55 ). The treatment rankings from this model are displayed in Online Figure G1 (Online Appendix G). Based on the treatment rankings nivolumab plus ipilimumab has little chance of being the most effective treatment. However, this is driven by the large uncertainty surrounding the effectiveness of selumetinib plus dacarbazine and ipilimumab plus sargramostin.

### 5.2 Restricted mean survival time

The difference in RMST at 18 months and 95% confidence intervals for each trial are reported in Online Appendix A. The improvement in RMST and 95% credible intervals for each treatment compared to dacarbazine from the fixed effect NMA model are presented in Figure 2(b). Based on the point estimate, 18 months RMST was the greatest for pembrolizumab (RMST=4.32, 95% CrI: 2.77, 5.91). The treatment rankings from this model are displayed in Online Figure G2 (Online Appendix G). Similarly to Cox PH NMA, the treatment rankings are driven by the large uncertainty surrounding the effectiveness of selumetinib plus dacarbazine and ipilimumab plus sargramostin.

### 5.3 Generalised gamma

The generalised gamma model was fitted with treatment modelled as a location parameter. The survival curves from this model are presented in Figure 3(b). Here, we can see that the generalised gamma model provides a reasonable fit to the observed data from the dacarbazine arm. The generalised gamma model predicts nivolumab plus ipilimumab and pembrolizumab as the most effective treatments with comparable survival curves over a 10 year period. The treatment rankings at 5 years are displayed in Online Figure G3 (Online Appendix G).

### 5.4 Piecewise exponential

Initially, we fitted the piecewise model including single cut points at 6 months and 12 months. In this model, the hazard rate varies across all time intervals and the cut point allows the treatment effect before 6 (or 12) months to differ to the treatment effect after 6 (or 12) months. The survival curves from the model with the cut point at 6 months are presented in Online Figure H1 (Online Appendix H) and from the model with the cut point at 12 months in Figure 3(c). In both plots, we see differences between the treatment arms emerging over time. In Online Figure H1, nivolumab plus ipilimumab appears to be the most effective treatment from approximately 12 months onwards. Whereas in Figure 3(c), nivolumab plus ipilimumab appears to be the most effective treatment from approximately 18 months onwards. For ipilimumab plus sargramostin there is a difference in the survival curve between Online Figure H1 and Figure 3(c). Moving the cut point from 6 to 12 months reduces the survival estimates beyond 12 months.

To allow the treatment effect to vary further, we also fitted a model with two cut points at 6 and 12 months. In this model, the hazard rate and the treatment effect vary across the three time intervals. Based on the DIC the model with a single cut point at 12 months (DIC = 610.9) is a better fitting model than the model with a single cut point at 6 months (DIC = 612.1) and the model with two cut points (DIC = 619.2). The survival curves from the model with cut points at 6 and 12 months is presented in Online Figure H2 (Online Appendix H). Compared to Figure 3(c), with two cut points we see differences both in shorter-term survival, with greater variation between treatments, and in longer-term survival, with marked differences for vemurafenib plus cobimetinib and dabrafenib plus trametinib.

### 5.5 Fractional polynomial

We fitted eight first-order fixed effect models, each taking a power from the set: −2,−1,−0.5,0,0.5,1,2,3 . With a burn in of 30,000 iterations and sample of 70,000 iterations we achieved convergence for the models with the powers −2,−1,−0.5,0,0.5 . After fitting each of these models, we visually compared the survival curves to the observed data (Figure 3(d) and Online Figures I1 to I4, Online Appendix I). Based on this and the DIC (Online Table I1, Online Appendix I), we identified the first-order model with power p=0 as the best fitting model, although we acknowledge that both the survival curves and DIC from the model with power p=0.5 were very similar. The survival curves from the model with p=0 are presented in Figure 3(d). The fractional polynomial model provides a reasonable fit to the observed data from the dacarbazine arm. As with the generalised gamma and piecewise exponential models, nivolumab plus ipilimumab appears to be the most effective treatment over time although in the fractional polynomial model this emerges slightly later, from approximately 24 months onwards (Figure 3(d)). At 5 years, nivolumab plus ipilimumab has 64% probability of being the most effective treatment in the network (Online Figure G5, Online Appendix G).

Based on the low DIC for the first-order models with p=0 and p=0.5 , we attempted to fit the following fixed effect second-order models: $p_{1}=0$ & $p_{2}=0.5$ , $p_{1}=0$ & $p_{2}=0$ , $p_{1}=0.5$ & $p_{2}=0.5$ . Despite a large burn in (400,000) and number of iterations (400,000) we were unable to achieve convergence for some of the parameters in all of the second-order fixed effect models. Refining the starting values and reducing the variance for the prior distributions did not result in convergence.

### 5.6 Royston-Parmar

An advantage of the Royston-Parmar (and generalised gamma) model over the piecewise exponential or fractional polynomial models is that, once the baseline log cumulative hazard has been chosen for each trial, we do not have to fit a large number of NMA models. The survival estimates from the non-PH model are presented in Figure 3(e). The shape of the survival curves is similar to the generalised gamma, piecewise exponential and fractional polynomial models allowing non-PH with nivolumab plus ipilimumab emerging as the most effective treatment after approximately 18 months. The probability of nivolumab plus ipilimumab as the most effective treatment at 5 years is 79% (Online Figure G6, Online Appendix G).

### 5.7 Area under the survival curve at 60 months

**Figure 4.**
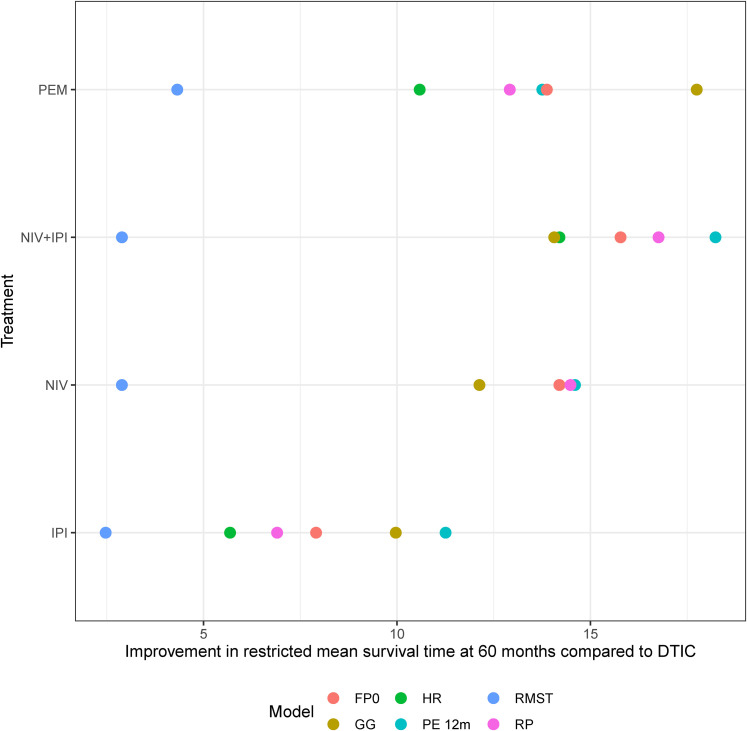
Improvement in restricted mean survival time at 60 months compared to dacarbazine from the generalised gamma, piecewise exponential, fractional polynomial and Royston-Parmar models. Improvement in restricted mean survival time at 18 months from the RMST model and at 51.5 months from the hazard ratio model. FP0 = fractional polynomial with *p*=0, GG = generalised gamma model with treatment modelled as a location parameter, HR = Cox proportional hazards model, PE 12 m = piecewise exponential model with cut point at 12 months, RMST = restricted mean survival time, RP = Royston-Parmar non-proportional hazards model. IPI = Ipilimumab, NIV = Nivolumab, PEM = Pembrolizumab.

To aid comparison of the different modelling approaches applied to the melanoma network, in Figure 4, we present the improvement in the area under the survival curve at 60 months for some key treatments compared to dacarbazine from each model and in Online Figure J1 (Online Appendix J) we present every treatment compared to dacarbazine from each model. For RMST, we present the improvement at 18 months as extrapolation beyond 18 months requires a parametric assumption and for the hazard ratio we present the improvement at 51.5 months as extrapolation beyond this would require a parametric assumption. Alongside Figure 3(a) to (e), we can see that different modelling approaches can result in different estimates of clinical effectiveness. In the melanoma network where we have non-PH and differing lengths of trial follow-up it is clear that the results from PH and non-PH models vary. However, we can also see that, in the melanoma network, all the models allowing for non-PH give similar results to each other and we consistently found nivolumab plus ipilimumab to be the most effective treatment from approximately 18 months onwards.

### 5.8 Model selection for the melanoma network

For the melanoma network, we selected the Royston-Parmar model as the most appropriate choice. We excluded the Cox PH model based on evidence of non-PH within some of the trials in the melanoma network and excluded the RMST model on the basis that we wished to extrapolate survival up to 10 years. To aid the process of selecting the most appropriate model for the melanoma network we plotted the survival curves for each treatment for each model alongside the Kaplan-Meier estimates of observed survival from the trials including the treatment of interest (Online Figures K1 to K13, Online Appendix K). Considering, Online Figure K1 which presents survival curves for nivolumab plus ipilimumab, the treatment selected as the most effective treatment beyond 2 years, the generalised gamma model showed a poor fit to the observed data and we excluded this model from further consideration. The piecewise exponential model resulted in an ‘odd’ shape to the estimated survival curves due to the instantaneous change in the hazard rate between time intervals and we excluded this model from further consideration. We then chose the Royston-Parmar model as it offered an improved fit to the observed data between 30 and 72 months compared to the fractional polynomial model. Across the remaining treatments, there is some variation in which model fits the observed data best and in some cases none of the models are a particularly good fit to the data (e.g. vemurafenib, Online Figure K12). However, we felt that the model which provided the best fit most often was the Royston-Parmar model.

## 6 Discussion

In this paper, we have discussed five alternative approaches to the Cox PH model for synthesising TTE outcomes in a NMA and provided guidance on key things to consider when choosing between the modelling approaches. We have illustrated the five modelling approaches and the key considerations for selecting between them using a melanoma network consisting of 13 trials.

Restricted mean survival time has been proposed as an alternative effect measure to the hazard ratio^25^ and is increasingly being used within the MA setting. Of the five methods we considered RMST is an outlier. It is the only method which cannot be easily extrapolated and it does not produce survival estimates so we were unable to produce survival curves to compare with the other models. Furthermore, the method used to estimate the difference in RMST has been shown to influence the results of cost-effectiveness analyses^57^. A key step in using RMST is the choice of time point for calculating RMST. Without extrapolation, this choice is restricted by the shortest follow-up time reported across the trials in the network. In the melanoma network, despite more than half of the trials reporting survival beyond 36 months we were restricted to calculating RMST at 18 months. To extrapolate RMST beyond 18 months would have required the assumption of a parametric survival function for survival beyond 18 months. In both a recent NMA comparing RMST with the hazard ratio for an IPD NMA of nasopharyngeal carcinomas^58^ and a simulation study comparing four methods for estimating RMST^56^ an exponential distribution was assumed to complete the tail of the Kaplan-Meier survival curve following the approach of Brown et al.^89^ Recent work by Gallacher et al.^90,91^ has shown that extrapolating RMST using a single parametric model can be unreliable and that it may be better to use a model averaging approach.

The generalised gamma model provides a flexible alternative to the Cox PH model for analysing TTE outcomes and is an accelerated failure time model. It is one of the few parametric distributions which allow a bathtub-shaped hazard.^52^ The advantage of using the generalised gamma approach over other parametric AFT models is that it can accommodate a wide variety of hazard functions and includes the Weibull, gamma and log-normal distributions as special cases and approximates the log-logistic distribution.^52^ This means that you do not need to specify in advance which type of hazard function you expect your data to have and allows for a variety of different shapes of hazard functions across the trials included in the NMA.^53^ As acknowledged by Cox, ‘choosing between parametric distributions can be difficult, yet the decision can have a considerable effect on the resulting inference’.^52^ We fitted the generalised gamma model using a similar approach to Cope et al.^92^ who propose a two-stage approach for synthesis of TTE data using multivariate NMA of survival function parameters. In the first stage, they estimate study-specific scale and shape parameters for each arm of each trial based on IPD and in the second stage, they use the multivariate NMA model proposed by Achana et al.^93^ to synthesise the parameters for each arm of each trial. They consider a range of distributions including exponential, Weibull and log-normal but not generalised gamma. In contrast, we fitted a generalised gamma model to each trial and synthesised at the trial level rather than the arm level. A limitation of our approach is that we only considered the generalised gamma model with the treatment effect on the location parameter restricting the shape of the hazard function. The full-flexibility of the generalised gamma model could be harnessed if we applied the treatment effect to the scale and/or shape parameters as well. However, further research is needed to demonstrate the transitivity assumption across multiple parameters, as has been previously done for two-parameter parametric models^37^ and fractional polynomial models.^27^ Alternative approaches to the generalised gamma distribution include the log-normal and log-logistic distributions which are two of the most common AFT models. Other approaches, of which the generalised gamma distribution is a member, include the generalised F distribution^94,95^ and beta generalised gamma distribution.^96^ A study comparing the five-parameter beta generalised gamma distribution to the three-parameter generalised gamma distribution concluded that the ‘beta generalised gamma distribution is not likely to be more useful for analytical purposes than the simpler generalised gamma distribution’.^97^ Two distributions which are also capable of modelling bathtub-shaped hazard functions are the Kumaraswamy generalised gamma distribution^98^ and the lognormal-power distribution^99^ whilst the exponentiated Weibull distribution has been shown to be strikingly similar to the generalised gamma distribution.^100^ A further extension of the generalised gamma distribution is the four parameter Marshall-Olkin generalised gamma distribution.^101^ However, we are not aware of these methods being used in the MA or NMA setting and further research is needed to assess whether these approaches would be suitable for evidence synthesis and to demonstrate the transitivity assumption across multiple parameters.

A key assumption of the piecewise exponential model is that the treatment effects are proportional within a time interval (but can vary across time intervals). Comparing models with differing time intervals is not straight forward as the choice of time intervals cannot be guided by model fit statistics as the data to which the models are fit changes if the time intervals are changed. Furthermore, the choice of where to place cut points and how many cut points could result in many models being fitted before the best model can be selected. To overcome the problem of where to place cut points and how many to have, Wiksten et al.^102^ propose a two-step process for fitting piecewise exponential (and fractional polynomial) models. In the first step, they use an ANOVA-like parameterisation to express the models as generalised linear models with time-varying covariates and fit the desired models in a frequentist framework. They compare the fit of the models in terms of the AIC and propose selecting the models with the lowest AIC to fit in the Bayesian setting in the second step. Implementation of this two-stage approach may speed up the model selection process. However, further research is needed to establish how often the best fitting model from the frequentist framework based on the AIC matches with the best fitting Bayesian model based on the DIC. Furthermore, it may be that instead of basing model selection on measures such as AIC and DIC, an alternative approach is needed. In this paper, we only considered a Bayesian framework however piecewise exponential models can also be fitted in the frequentist framework using Gauss-Hermite quadrature for maximum likelihood.^103^ We found that the assumption of an instantaneous change in the hazard rate between time intervals can lead to an ‘odd’ shape of the survival curve suggesting a lack of biological plausibility and, in agreement with Latimer,^42^ we found that piecewise exponential models were not the best approach for extrapolating survival curves beyond the observed data.

The fractional polynomial approach can accommodate a wide range of baseline hazards making it one of the most flexible approaches we considered. However, the wide choice of models means that analysis can be time consuming as it is not always immediately obvious which combination of powers will prove to be the best model. The two-stage approach of Wiksten et al.^102^ can be applied to fractional polynomial models and may help speed up the model selection process. In the first stage, the ANOVA-like parametrisation can be used to fit all eight first-order and 36 second-order fractional polynomial models in a frequentist framework before selecting the model with the lowest AIC to fit in the Bayesian framework^102^. Fractional polynomial models tend to be highly parametrised and we found them sensitive to starting values. However, we believe the problems we encountered were due to the structure of our network – many treatments and few head-to-head trials. Further research is needed to establish whether different modelling approaches are more suited to particular network structures than others and to determine the minimum data requirements for each type of model. Despite these potential problems, the greatest advantage of fractional polynomial models is the large amount of flexibility they offer in accounting for non-PH in NMA of TTE outcomes and they are a popular choice.

In a recent simulation study, fractional polynomial models^104^ were compared with the mixed treatment comparison approach of Dakin et al.^105^ and the integrated two-component prediction approach of Ding et al.^106^ in the setting of an NMA of longitudinal data with a binary outcome.^107^ Fractional polynomial models were found to be the most flexible approach and were able to accommodate different time patterns. Similarly to ourselves, Tallarita et al.^107^ also noted that fractional polynomial models require a large number of models to be fitted in order to select the optimal power terms for the polynomials. Further work by Heinecke et al.^108^ proposed an NMA method based on B-splines to allow simultaneous assessment of outcomes across different time points accounting for correlation across time and compared its performance to the fractional polynomial approach. Although the authors do not consider TTE outcomes they state that the model can be applied to any outcome for which an appropriate link function can be specified.^108^ Another approach for synthesising TTE outcomes reported at multiple time points which only requires study-level data is a multivariate MA model which uses exact binomial within-study distributions and enforces constraints that both the study specific and overall mortality rates must not decrease over time.^109^ A further approach for synthesising outcomes reported at multiple time points that has been proposed for continuous outcomes and not yet applied to TTE outcomes is a model-based NMA framework which models the treatment effect with a piecewise linear function.^110^

Through the use of restricted cubic splines the Royston-Parmar model provides a flexible parametric alternative to the Cox model. A restricted cubic spline is used to model the baseline log cumulative hazard for each trial. An advantage of this approach over the fractional polynomial models is that the restricted cubic splines are forced to be linear at each end which reduces the possibility of unexpected end effects which may also reduce the number of iterations needed to achieve convergence.^48^ Long-term extrapolation using spline models incorporating external data with trial data has been shown to be more reliable that long-term extrapolation using parametric models based on trial data only.^111^

In this paper, we have illustrated the different modelling approaches through application to a melanoma network. Whilst the melanoma network has a number of strengths, it also has several limitations. Since 2013, in the UK, the National Institute for Health and Care Excellence (NICE) has recommended NMA as their preferred method for evidence synthesis to assess the clinical effectiveness from all relevant studies reporting clinically relevant outcomes^112^. Therefore, the melanoma network in which a variety of treatment options are considered including newer immuno-oncologic therapies, such as BRAF, MEK and PD-1 inhibitors, as well as traditional chemotherapy regimens reflects a commonly encountered situation in which there are many treatment options available but a limited amount of direct head-to-head evidence. In the melanoma network, only one comparison in the network is informed by more than one trial. Therefore, the NMA may provide only a slight improvement over pairwise meta-analyses and individual trial estimates. The lack of treatment loops in the network prevented the assessment of consistency between the direct and indirect evidence. Furthermore, the use of reconstructed IPD meant that we did not have access to covariate data and were unable to adjust our analyses to take important covariates into account.

The structure of the melanoma network meant it was only appropriate to fit fixed treatment effect models. However, the modelling approaches we discussed can all be applied as random treatment effect models. In the case of the Royston-Parmar and piecewise exponential models, an obvious choice for modelling the between-study heterogeneity is to use an inverse Wishart prior distribution. The inverse Wishart prior distribution is commonly used to model the between-study heterogeneity in NMA as it is the conjugate prior distribution for multivariate normal models.^113,114^ However, it has been shown that in a multivariate meta-analysis setting the Wishart prior may not always be the most appropriate choice of prior distribution.^113,114^ The inverse Wishart distribution may become influential in the estimation of the between-study variance-covariance matrix leading to overestimation of the heterogeneity parameter, particularly when the heterogeneity is close to zero.^113^ One advantage of conducting NMA in the Bayesian framework is that prior distributions can be informed by empirical evidence which could result in more realistic prior distributions. Turner et al.^115^ propose three alternatives to the inverse Wishart prior distribution when external evidence is available. A formal simulation study is required to compare the performance of these alternatives to the inverse Wishart prior distribution.

We considered parametric, non-parametric and accelerated failure time models as alternatives to the popular semi-parametric Cox model for analysing TTE outcomes under a Bayesian framework. We considered hazard ratios for time intervals, multi-dimensional treatment effects, accelerated failure time and restricted mean survival time. Some of these approaches have been explored in recent NICE appraisals (e.g. TA520, TA522, TA525, TA557)^116–119^ as well as the scientific literature more generally^120^. We also compared mean survival up to 60 months.^121^. However, we did not consider alternative measures of effect size, such as the percentile ratio. The percentile ratio is the ratio of survival distributions at a specified percentile^53,54^ but to the authors knowledge, to date, this has not been used in the NMA setting.

In this paper, we have focused on modelling the hazard function. However, we did not consider modelling the hazard function in a semi-parametric logistic regression model using B-splines to model the baseline time effect.^122^ An alternative approach to modelling the hazard function which we did not consider is to synthesise survival curves. This approach has been considered by a number of authors as a method for meta-analysing survival proportions reported across multiple time points in which the survival curve is modelled for each arm in each study and the treatment effect calculated based on the survival curve for each arm.^123^ Dear^124^ proposed a fixed effect iterative approach using generalised least squares and Arends et al.^125^ extended this approach to allow for random effects. Another approach extends the Poisson correlated gamma-frailty model to synthesise survival proportions reported at multiple times in different studies allowing for heterogeneity between studies.^126^ Whilst a further approach proposes fixed and random effects methods for multivariate meta-analysis of effect sizes reported at multiple time points.^123^ However, to derive the correlation between time points the same number of patients at baseline and subsequent time points must be assumed. Therefore, this approach is not optimal for outcomes in which censoring matters.^123^

The PH assumption can be assessed in a number of ways. Some of the most commonly used methods are: visual inspection of the log cumulative hazard plot, visual inspection of the scaled Schoenfeld residuals and the Grambsch-Therneau test of the Schoenfeld residuals.^127,51,128,129^ However, one thing that remains unclear for an NMA is how many trials exhibiting evidence of non-PH are required for the PH assumption to be violated. A review of HTA guidelines found considerable variation in approaches to non-PH both across and within HTA agencies.^26^ However, despite guidelines demonstrating awareness of the importance of the PH assessment only three (of 10) recommend testing of the PH assumption.^26^ At the individual trial level, Uno et al.^35^ suggest that when studies have large number of events PH models would likely be rejected even with minor departures from true proportionality. Two reviews^130,58^ comparing difference in RMST with HR found that at the trial level, conclusions based on the difference in RMST corresponded to conclusions based on the HR. One review identified that the magnitude of the treatment effect given by the HR was systematically greater than the difference in RMST^130^ and in the other, that the choice of difference in RMST and HR affected the direction of the treatment effect at the NMA level.^58^ Furthermore, if one trial deviates from the PH assumption then there will be bias but the extent of the bias will depend on characteristics such as network size. In some cases, non-PH may be handled more naturally using models that assume proportionality on a different scale, e.g., proportional odds.^17^ Furthermore, when there is a biological reason why PH would not be appropriate (e.g. NMA including chemotherapy and immunotherapy) then models allowing for non-PH should be used as a matter of course.

The melanoma network highlights the importance of the decision making criteria. Different modelling approaches may select different treatments as the most effective and in the presence of non-PH the most effective treatment may change over time. The choice of model can have a significant impact on uncertainty around the extrapolated survival function and cost-effectiveness, even when results are similar.^131^ Incorporating expert opinion has been shown to improve precision in extrapolated survival curves.^132^ In the melanoma network, nivolumab plus ipilimumab was consistently reported as the most effective treatment from 24 months onwards by the generalised gamma, piecewise exponential, fractional polynomial and Royston-Parmar models. However, the time point at which it became the most effective treatment varied across the modelling approaches and the treatment most effective prior to 24 months also varied by modelling approach. To further compare the modelling approaches we also calculated the probability of each treatment obtaining each rank from 1 to 13 to allow us to compare which treatments were being identified as the most effective across the different modelling approaches. However, due to a large amount of uncertainty in the ranking probabilities these should be interpreted with caution. We did not formally compare the modelling approaches in this paper as to do so will require a simulation study.

Whichever modelling approach is chosen, when the model results inform the decision-making process a key consideration should be how easy the treatment effect parameters are to incorporate within a decision model. For example, the Royston-Parmar and piecewise exponential models can report log hazard ratios for each time interval and from the generalised gamma model we obtain accelerated failure times. The parameters from the fractional polynomial models are much harder to interpret intuitively. A popular choice of decision model for NICE technology appraisals reporting TTE outcomes is the partitioned survival analysis model.^59^ A partitioned survival analysis model is constructed by calculating the proportion of patients in different health states (i.e. healthy, progressed, death) based on overall survival and progression-free survival curves at discrete time points. This approach allows the modelling of overall and progression-free survival to be based on observed events which can accurately reflect the disease progression and long-term survival profile of patients.^133^ Except RMST, the other four modelling approaches considered in this paper can be easily incorporated within a partitioned survival analysis model.

Ultimately, deciding on the right approach for NMA of TTE outcomes is not straight forward. We have shown that the RMST, generalised gamma, piecewise exponential, fractional polynomial and Royston-Parmar models can accommodate non-PH and differing lengths of trial follow-up within an NMA of TTE outcomes. However, for every NMA the choice of which model to select will be informed by different things. An holistic approach considering a wide range of factors including prior belief and model transparency, and not just model fit, can improve decision making. We recommend that the key considerations used to inform this decision are:

- using available and relevant prior knowledge to inform the choice of model and/or prior distributions;
- model transparency;
- graphically comparing survival curves alongside observed data to aid consideration of the reliability of the survival estimates;
- consideration of how the treatment effect estimates can be incorporated within a decision model.

## Supplemental Material

sj-docx-1-smm-10.1177_09622802211070253 - Supplemental material for Challenges of modelling approaches for network meta-analysis of time-to-event outcomes in the presence of non-proportional hazards to aid decision making: Application to a melanoma network Supplemental material, sj-docx-1-smm-10.1177_09622802211070253 for Challenges of modelling approaches for network meta-analysis of time-to-event outcomes in the presence of non-proportional hazards to aid decision making: Application to a melanoma network by Suzanne C Freeman, Nicola J Cooper, Alex J Sutton, Michael J Crowther, James R Carpenter and Neil Hawkins in Statistical Methods in Medical Research
