# Supplementary material for: Challenges of modelling approaches for network meta-analysis of time-to-event outcomes in the presence of non-proportional hazards to aid decision making: Application to a melanoma network
Source: Stat Methods Med Res. 2022 Jan 19;31(5):839–61. doi: 10.1177/09622802211070253 (PMC9014691; doi:10.1177/09622802211070253)
Supplement: sj-docx-1-smm-10.1177_09622802211070253 - Supplemental material for Challenges of modelling approaches for network meta-analysis of time-to-event outcomes in the presence of non-proportional hazards to aid decision making: Application to a melanoma network [file sj-docx-1-smm-10.1177_09622802211070253.docx]

Challenges of modelling approaches for network meta-analysis of time-to-event outcomes in the presence of non-proportional hazards to aid decision making: application to a melanoma network

Supplementary material

SC Freeman, NJ Cooper, AJ Sutton, MJ Crowther, JR Carpenter, N Hawkins

# Appendix A: Melanoma trial characteristics

| **Trial** | **Treatment arm** | **No. of patients** | **Median follow-up (interquartile range) (months)** | **Hazard Ratio (95% confidence interval)*** | **RMST at 18 months (95% confidence interval)**** |
| --- | --- | --- | --- | --- | --- |
| BREAK-3 ^1^ | Dacarbazine | 63 | 61.7 (47.5 - 63.4) |  |  |
|  | Dabrafenib | 187 | 61.8 (58.3 – 63.4) | 0.82 (0.58, 1.16) | 0.74 ( -0.84, 2.32) |
| BRIM-3 ^2^ | Dacarbazine | 338 | 49.0 (45.6 – 52.1) |  |  |
|  | Vemurafenib | 337 | 50.1 (46.7 - 53.7) | 0.80 (0.67, 0.95) | 1.95 (1.02, 2.88) |
| CheckMate 066 ^3^ | Dacarbazine | 208 | 44.7 (40.8 – 47.6) |  |  |
|  | Nivolumab | 210 | 45.7 (41.7 – 47.5) | 0.46 (0.36, 0.59) | 2.92 (1.76, 4.09) |
| CheckMate 067 ^4^ | Ipilimumab | 315 | 49.7 (48.7 – 50.4) |  |  |
|  | Nivolumab | 316 | 63.7 (61.6 – 65.1) | 0.65 (0.53, 0.80) | 0.41 (-0.11, 0.92) |
|  | Nivolumab + Ipilimumab | 314 | 63.6 (61.6 – 65.2) | 0.55 (0.44, 0.68) | -0.45 (-1.21, 0.31) |
| CheckMate 069 ^5^ | Ipilimumab | 47 | 25.7 (24.7 – 26.7) |  |  |
|  | Nivolumab + Ipilimumab | 95 | 25.7 (24.9 – 26.6) | 0.75 (0.44, 1.28) | 0.69 (-1.47, 2.84) |
| COBRIM ^6^ | Vemurafenib | 248 | 23.2 (19.9 – 26.0) |  |  |
|  | Vemurafenib + Cobimetinib | 247 | 23.2 (21.3 – 25.6) | 0.69 (0.54, 0.88) | 1.41 (0.46, 2.35) |
| COMBI-d ^7^ | Dabrafenib | 211 | 38.9 (37.0 – 40.9) |  |  |
|  | Dabrafenib + Trametinib | 211 | 39.3 (36.9 – 41.4) | 0.76 (0.59, 0.97) | 1.17 (0.15, 2.18) |
| COMBI-v ^8^ | Dabrafenib + Trametinib | 352 | 13.1 (11.1 – 15.3) |  |  |
|  | Vemurafenib | 352 | 12.3 (10.6 – 14.6) | 1.40 (1.08, 1.83) | -1.12 (-1.93, -0.30) |
| Hodi 2014 ^9^ | Ipilimumab | 122 | 13.9 (12.2 – 15.9) |  |  |
|  | Ipilimumab + Sargramostin | 123 | 13.8 (11.8 – 15.5) | 0.67 (0.45, 0.99) | 1.75 (0.13, 3.38) |
| Keynote 006 ^10^ | Ipilimumab | 278 | 57.8 (55.7 – 59.8) |  |  |
|  | Pembrolizumab | 556 | 58.0 (56.1 – 59.9) | 0.73 (0.61, 0.88) | 1.85 ( 0.96, 2.74) |
| Ribas 2013 ^11^ | Dacarbazine | 327 | 37.0 (33.1 – 41.5) |  |  |
|  | Tremelimumab | 328 | 37.5 (33.4 – 43.6) | 0.88 (0.74, 1.04) | 0.55 (-0.38, 1.47) |
| Robert 2011 ^12^ | Dacarbazine | 252 | 63.3 (61.5 – 66.9) |  |  |
|  | Ipilimumab + Dacarbazine | 250 | 63.1 (61.2 – 66.2) | 0.73 (0.60, 0.88) | 1.41 (0.32, 2.49) |
| Robert 2013 ^13^ | Dacarbazine | 46 | 22.9 (21.1 – 23.6) |  |  |
|  | Selumetinib + Dacarbazine | 45 | 21.3 (20.7 – 21.9) | 0.83 (0.51, 1.34) | 0.86 (-1.37, 3.08) |

RMST = restricted mean survival time

* Hazard ratio and 95% confidence interval from Cox proportional hazards model fitted to IPD extracted from the publication referenced in the trial column

** Restricted mean survival time and 95% confidence interval from Cox proportional hazards model fitted to IPD extracted from the publication referenced in the trial column

**References**

1. Hauschild A, Ascierto PA, Schadendorf D, et al. Long-term outcomes in patients with BRAF V600-mutant metastatic melanoma receiving dabrafenib monotherapy: Analysis from phase 2 and 3 clinical trials. *Eur J Cancer.* 2020;125:114-120.

2. Chapman PB, Robert C, Larkin J, et al. Vemurafenib in patients with BRAFV600 mutation-positive metastatic melanoma: final overall survival results of the randomized BRIM-3 study. *Ann Oncol.* 2017;28(10):2581-2587.

3. Ascierto PA, Long GV, Robert C, et al. Survival Outcomes in Patients With Previously Untreated BRAF Wild-Type Advanced Melanoma Treated With Nivolumab Therapy: Three-Year Follow-up of a Randomized Phase 3 Trial. *JAMA Oncol.* 2019;5(2):187-194.

4. Larkin J, Chiarion-Sileni V, Gonzalez R, et al. Five-Year Survival with Combined Nivolumab and Ipilimumab in Advanced Melanoma. *N Engl J Med.* 2019;381(16):1535-1546.

5. Hodi FS, Chesney J, Pavlick AC, et al. Combined nivolumab and ipilimumab versus ipilimumab alone in patients with advanced melanoma: 2-year overall survival outcomes in a multicentre, randomised, controlled, phase 2 trial. *The Lancet Oncology.* 2016;17(11):1558-1568.

6. Ascierto PA, McArthur GA, Dréno B, et al. Cobimetinib combined with vemurafenib in advanced BRAFV600-mutant melanoma (coBRIM): updated efficacy results from a randomised, double-blind, phase 3 trial. *The Lancet Oncology.* 2016;17(9):1248-1260.

7. Long GV, Flaherty KT, Stroyakovskiy D, et al. Dabrafenib plus trametinib versus dabrafenib monotherapy in patients with metastatic BRAF V600E/K-mutant melanoma: long-term survival and safety analysis of a phase 3 study. *Ann Oncol.* 2017;28(7):1631-1639.

8. Robert C, Karaszewska B, Schachter J, et al. Improved overall survival in melanoma with combined dabrafenib and trametinib. *N Engl J Med.* 2015;372(1):30-39.

9. Hodi FS, Lee S, McDermott DF, et al. Ipilimumab plus sargramostim vs ipilimumab alone for treatment of metastatic melanoma: a randomized clinical trial. *JAMA.* 2014;312(17):1744-1753.

10. Robert C, Ribas A, Schachter J, et al. Pembrolizumab versus ipilimumab in advanced melanoma (KEYNOTE-006): post-hoc 5-year results from an open-label, multicentre, randomised, controlled, phase 3 study. *The Lancet Oncology.* 2019;20(9):1239-1251.

11. Ribas A, Kefford R, Marshall MA, et al. Phase III randomized clinical trial comparing tremelimumab with standard-of-care chemotherapy in patients with advanced melanoma. *J Clin Oncol.* 2013;31(5):616-622.

12. Robert C, Thomas L, Bondarenko I, et al. Ipilimumab plus Dacarbazine for Previously Untreated Metastatic Melanoma. *N Engl J Med.* 2011;364:2517-2526.

13. Robert C, Dummer R, Gutzmer R, et al. Selumetinib plus dacarbazine versus placebo plus dacarbazine as first-line treatment for BRAF-mutant metastatic melanoma: a phase 2 double-blind randomised study. *The Lancet Oncology.* 2013;14(8):733-740.

# Appendix B: Kaplan-Meier plots of survival time

Note: Larger images of the individual trial plots can be viewed from the next page onwards


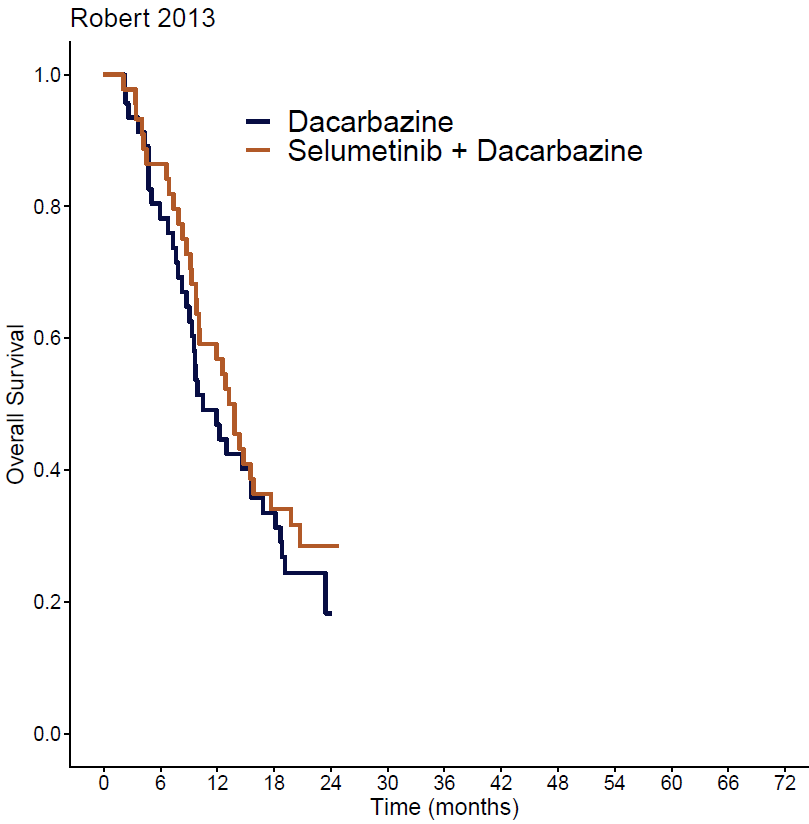

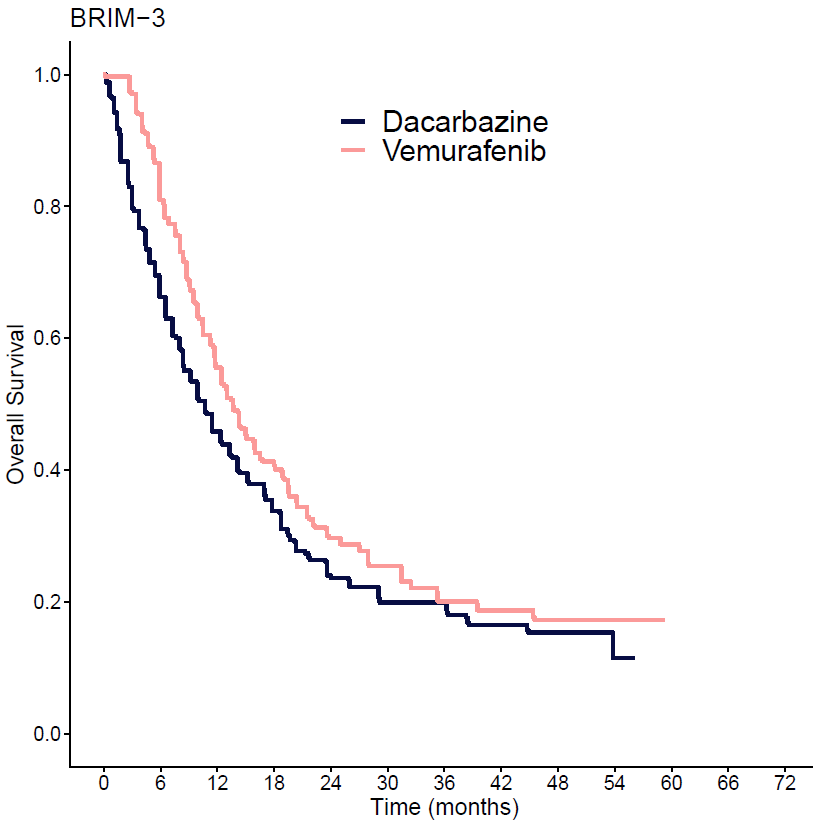

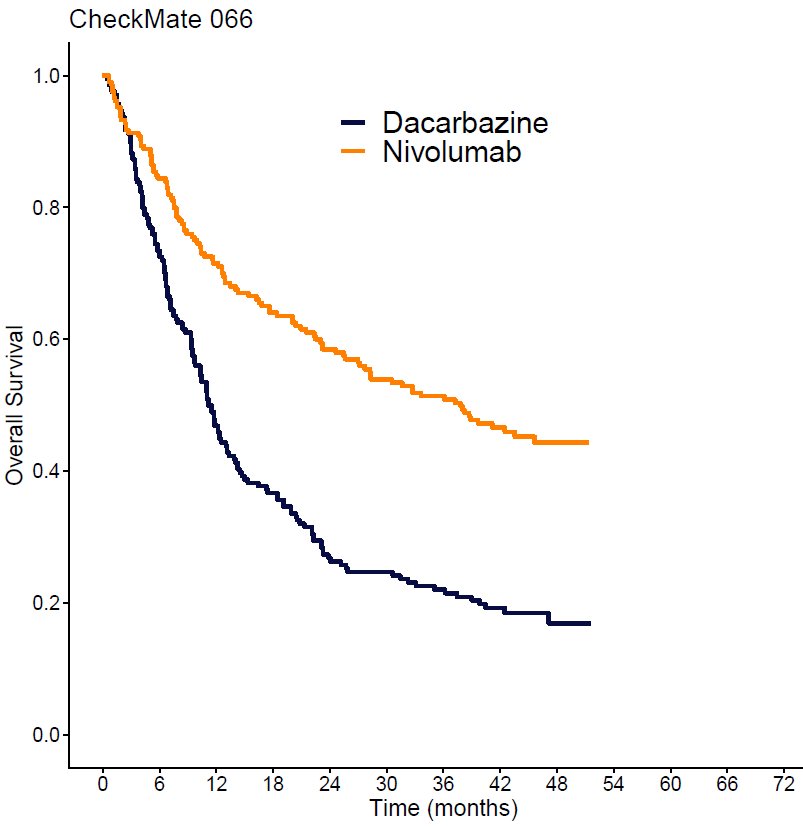

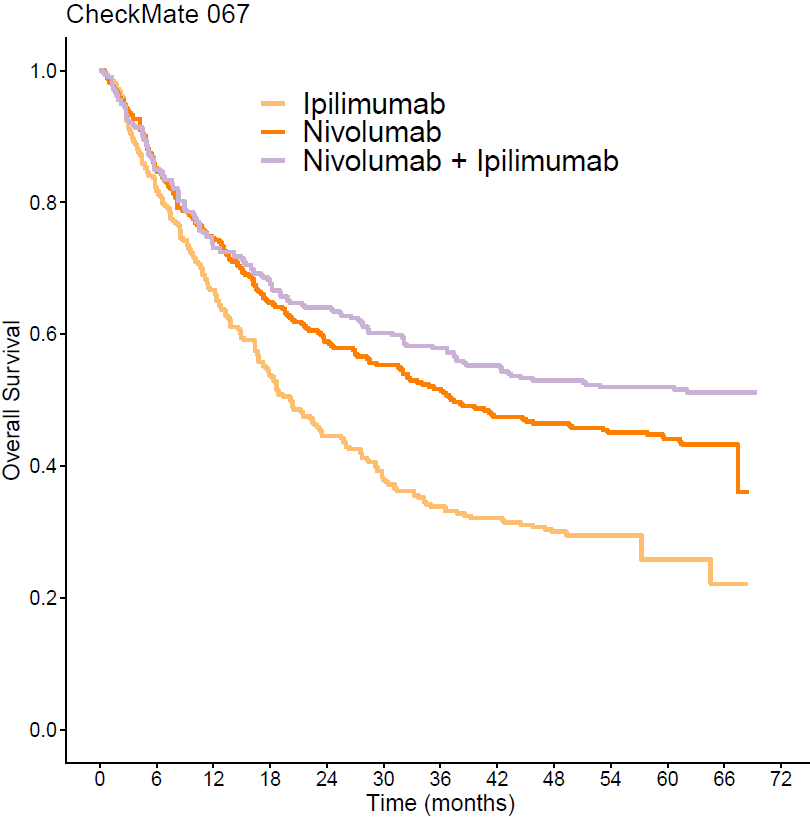

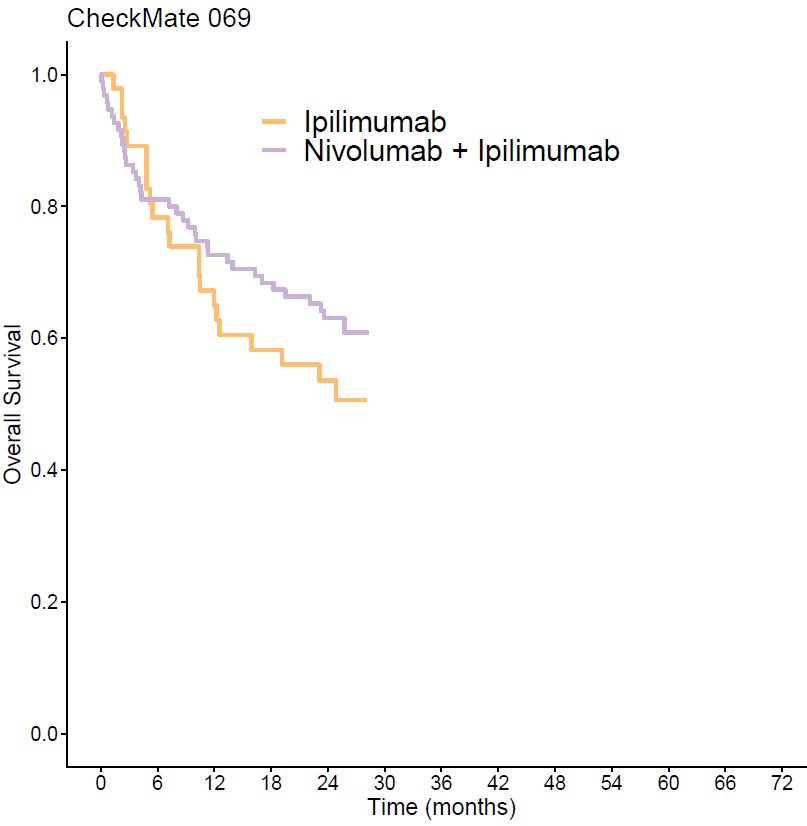

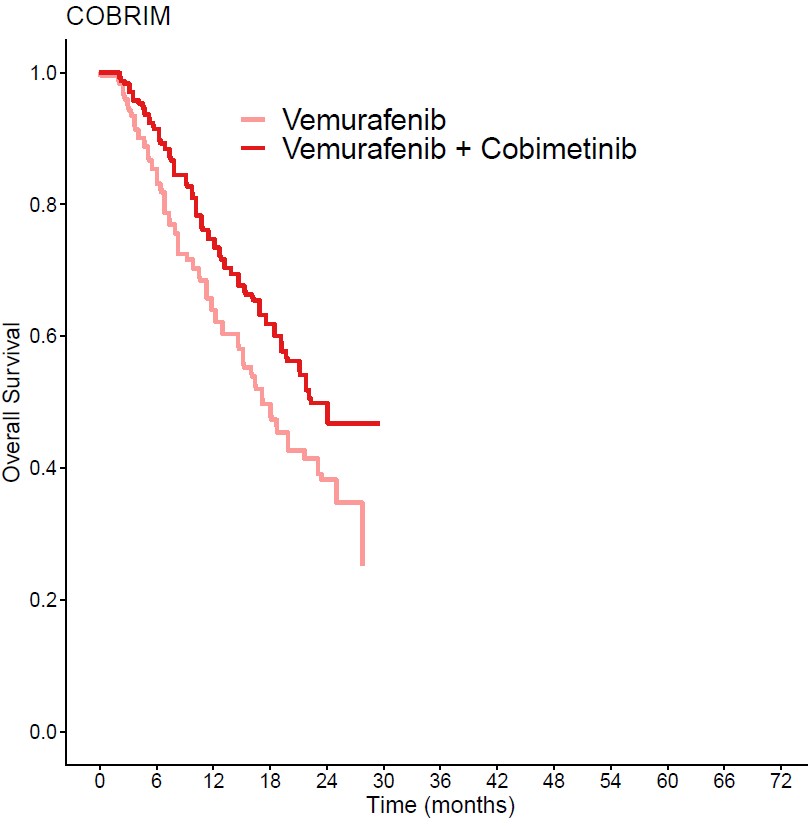

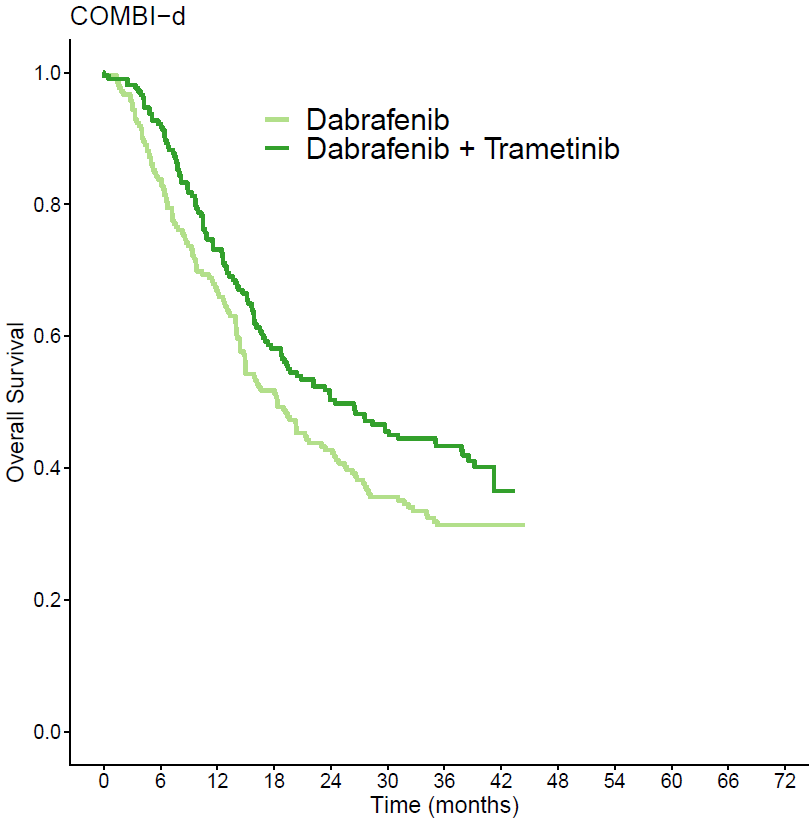

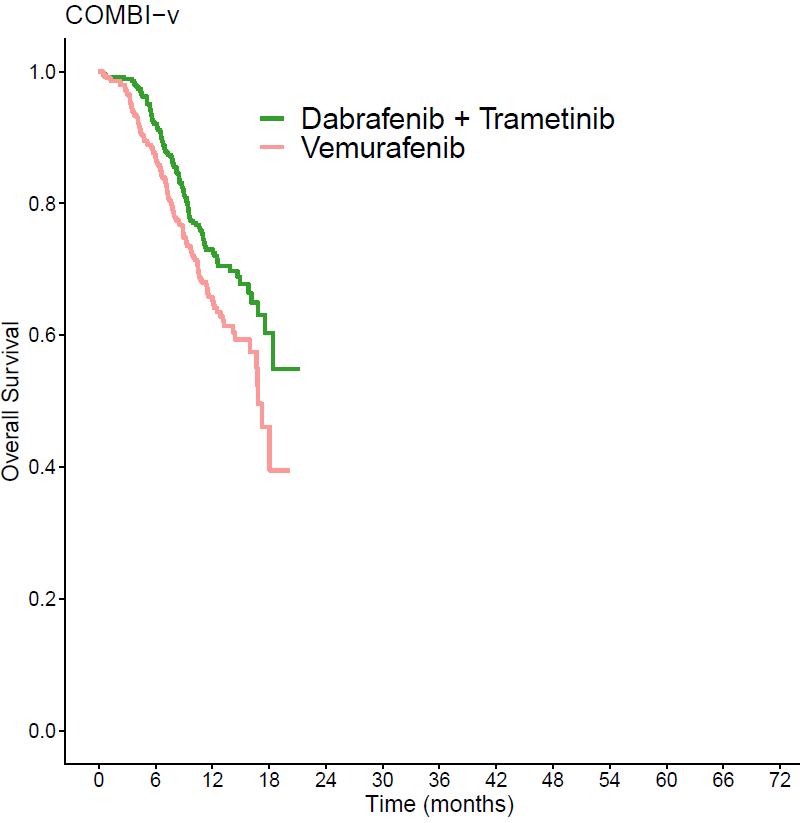

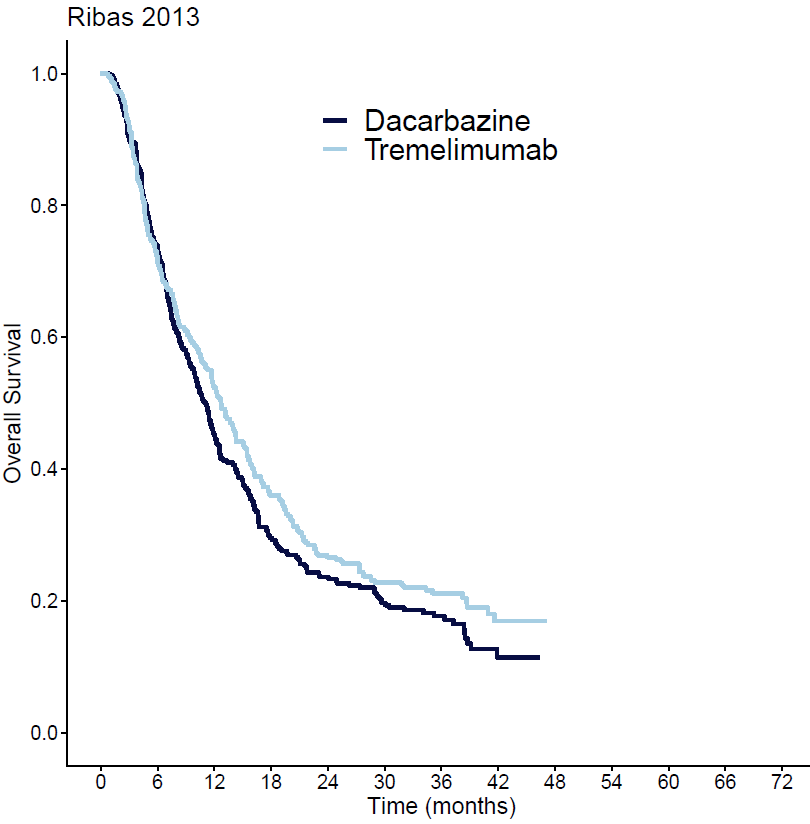

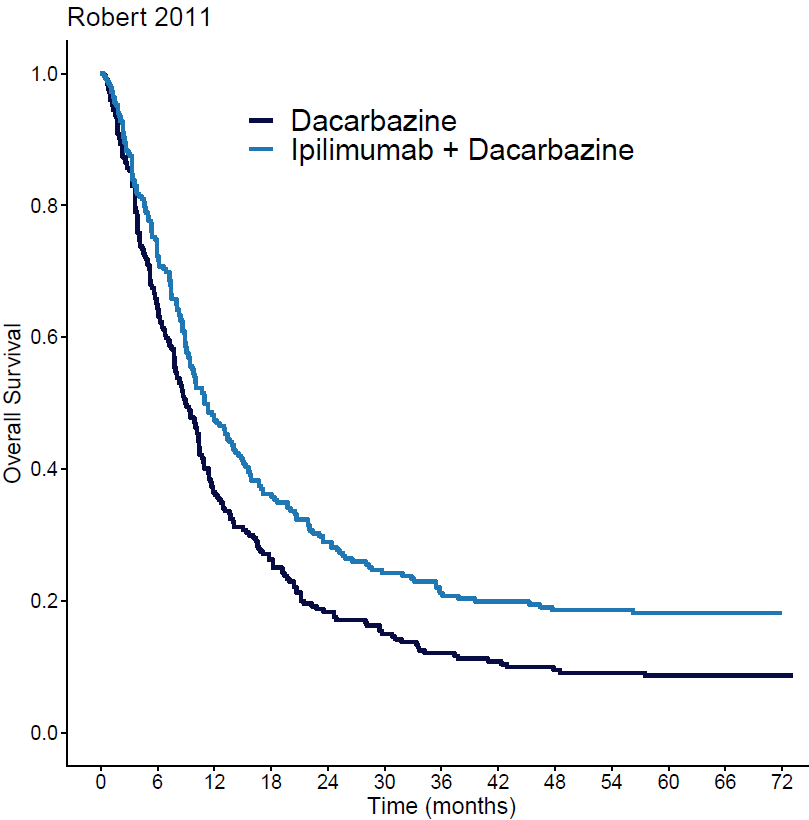

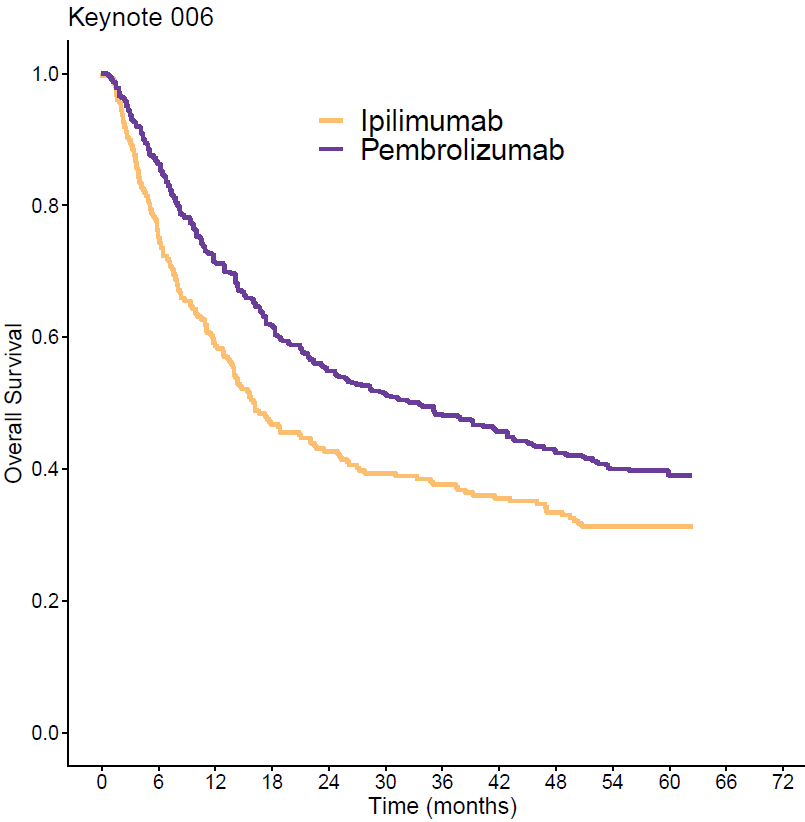

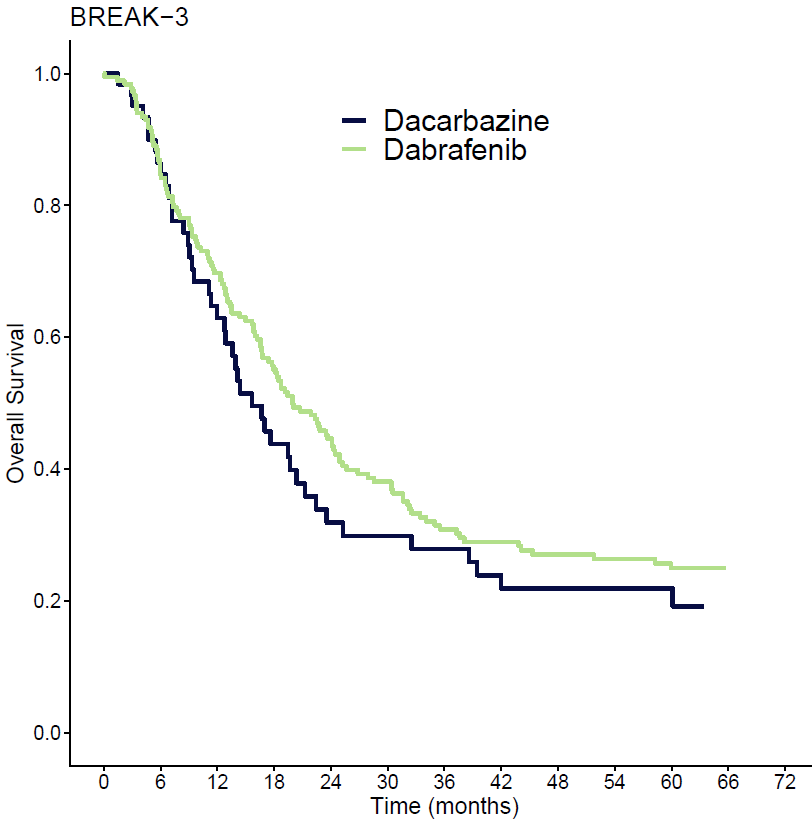

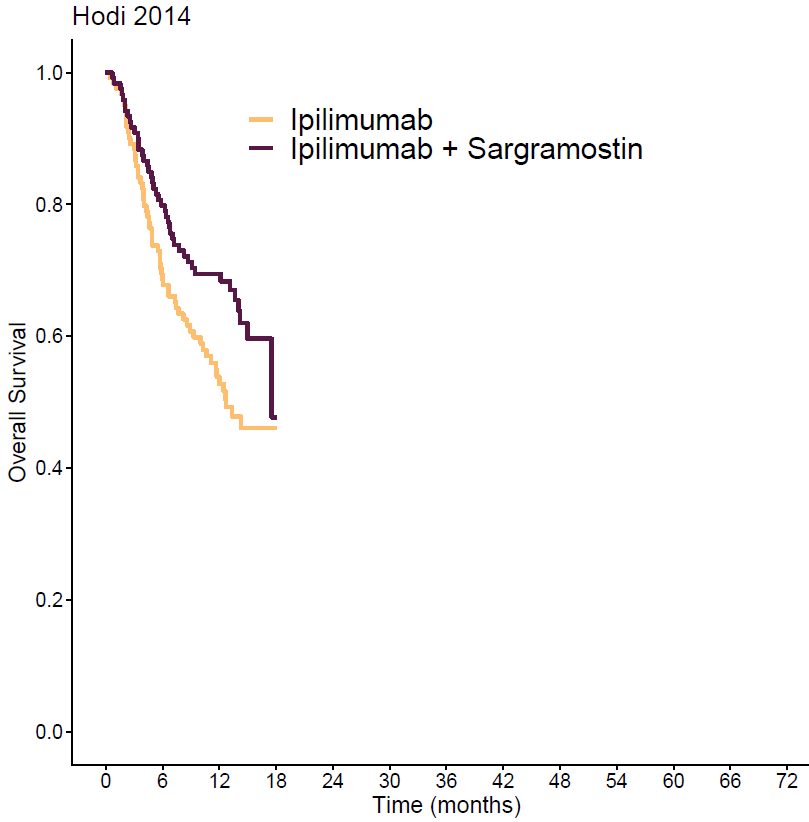


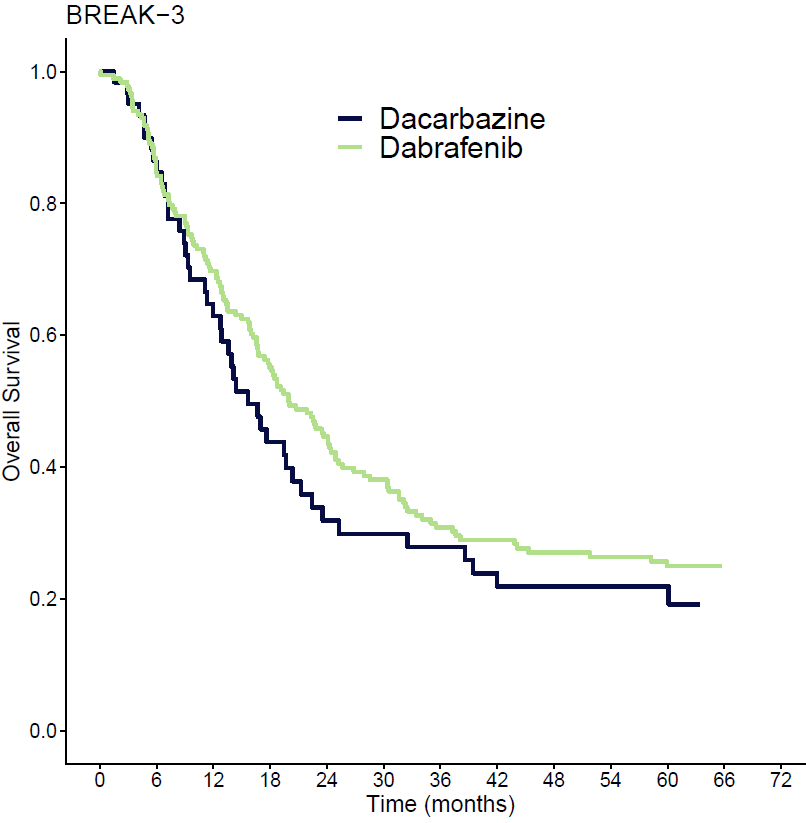


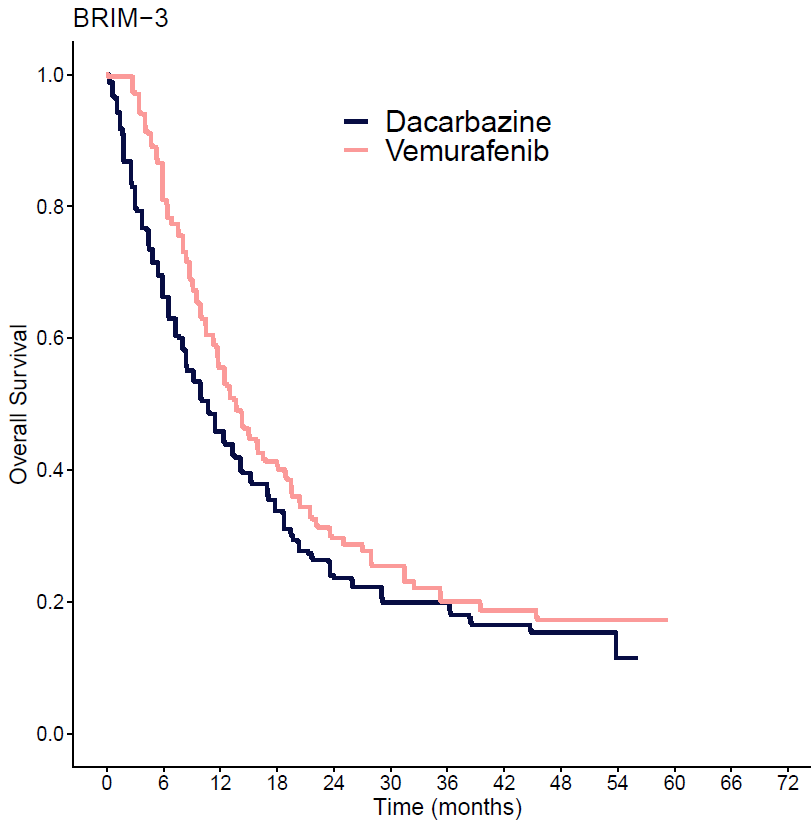


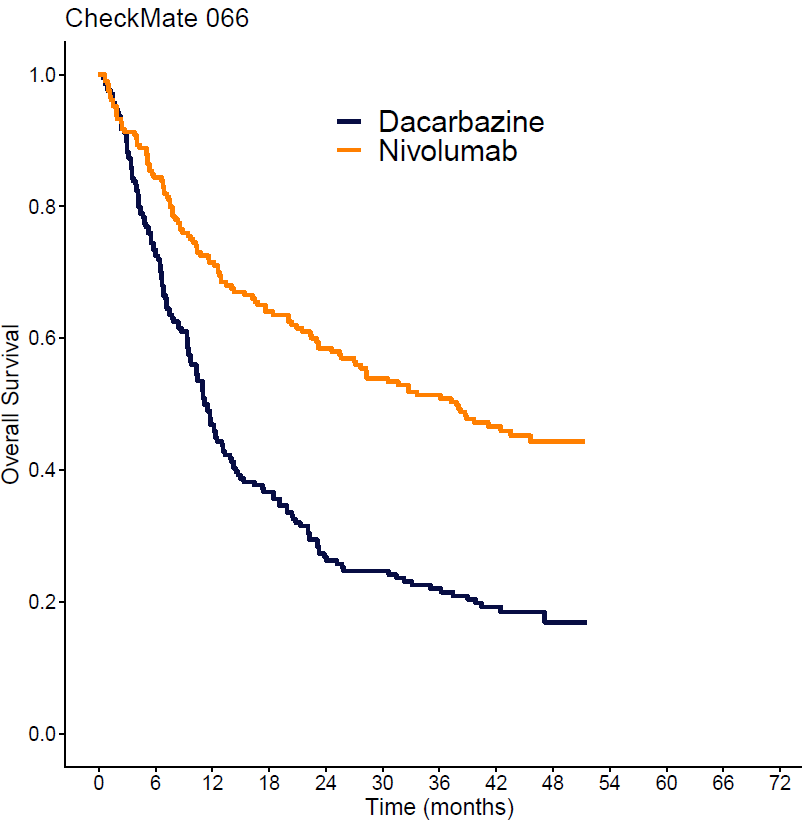


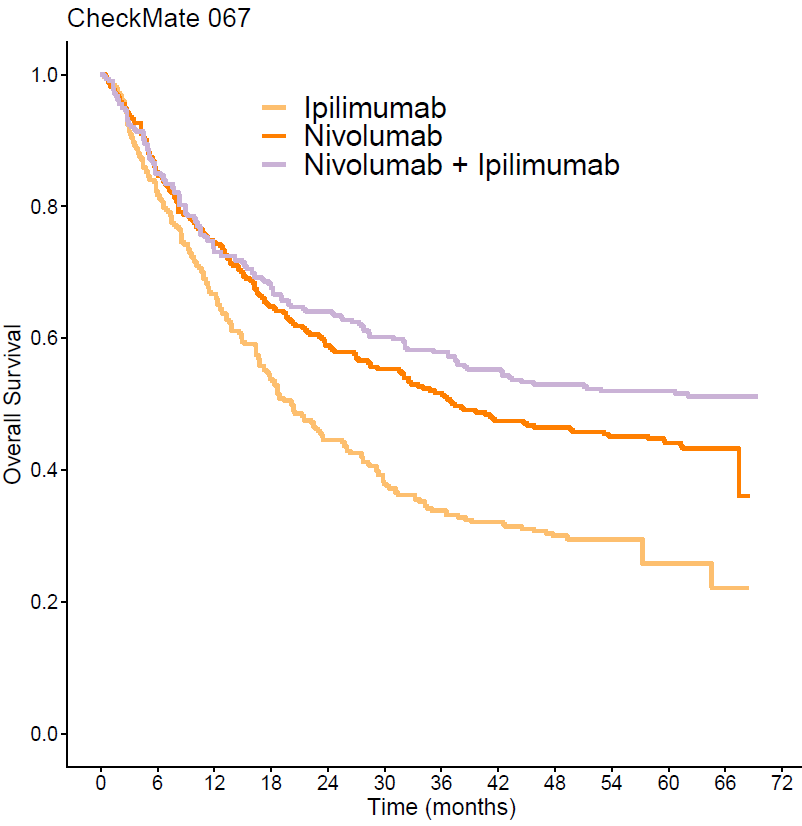


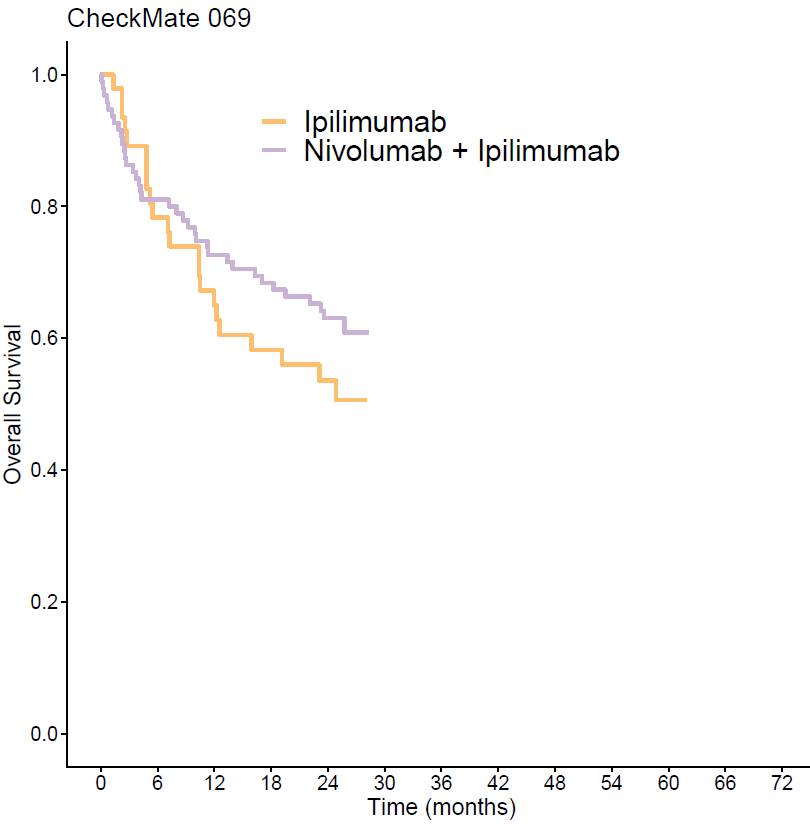


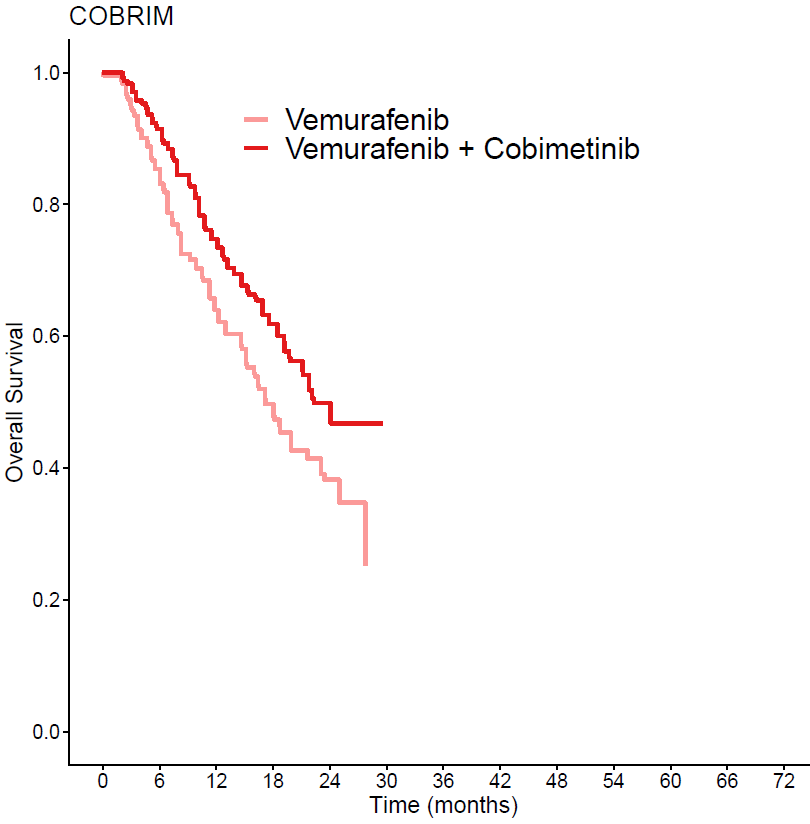


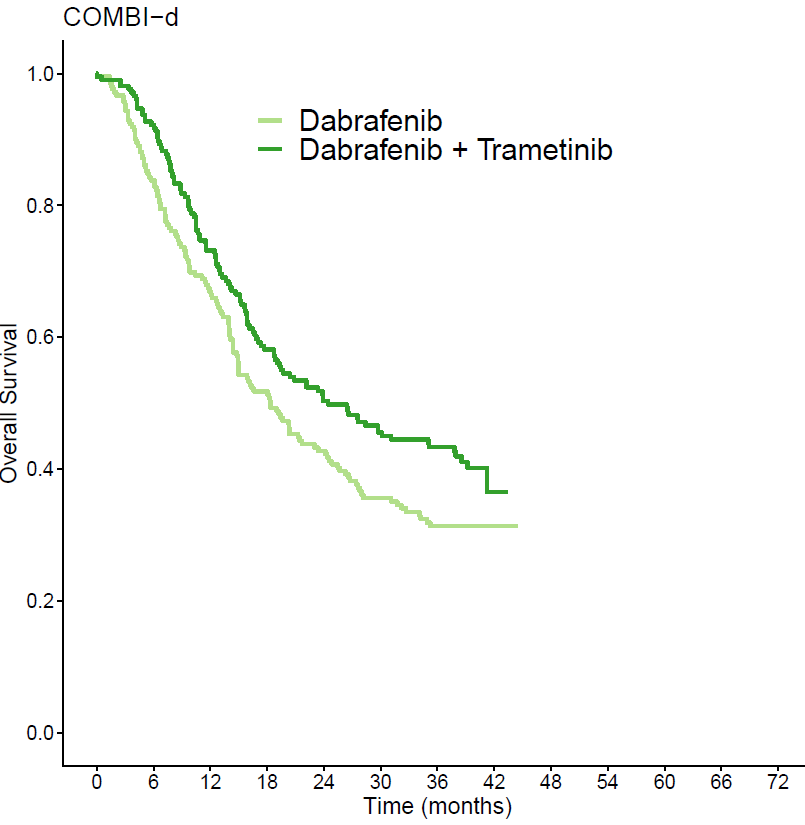


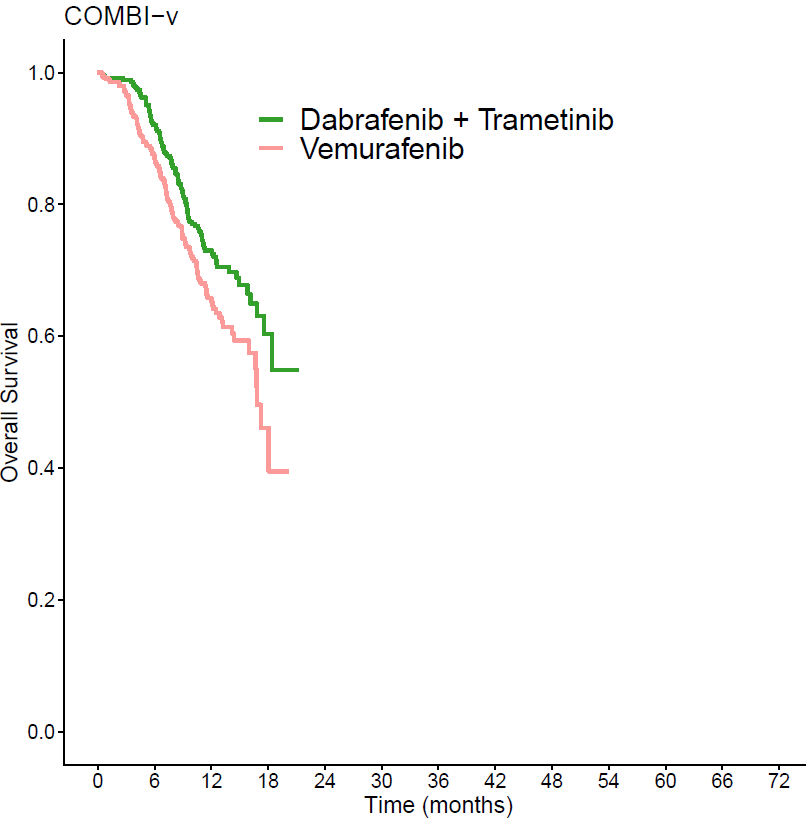


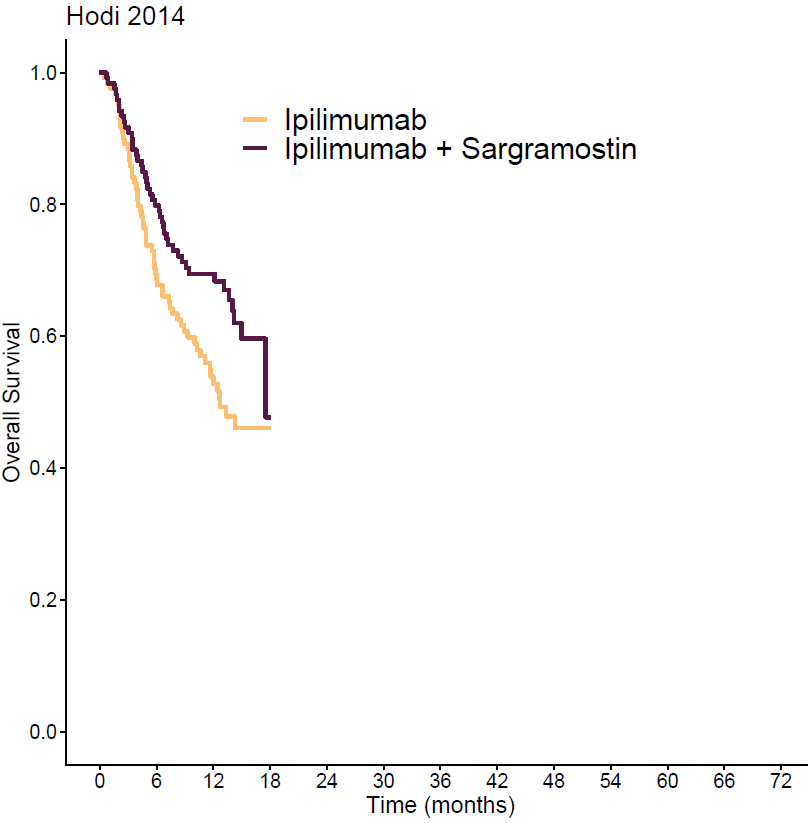


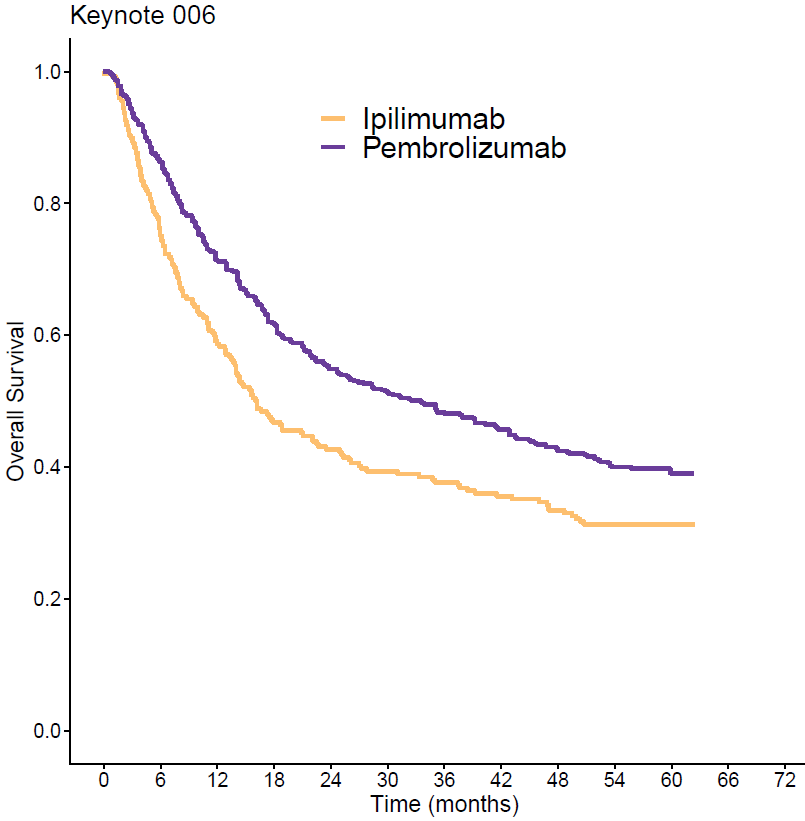


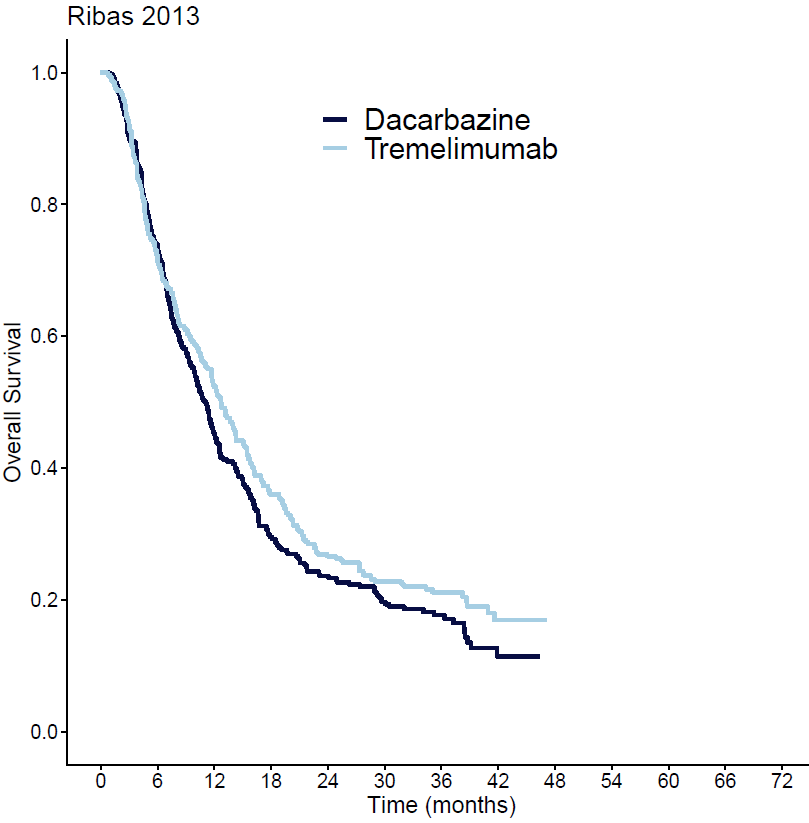


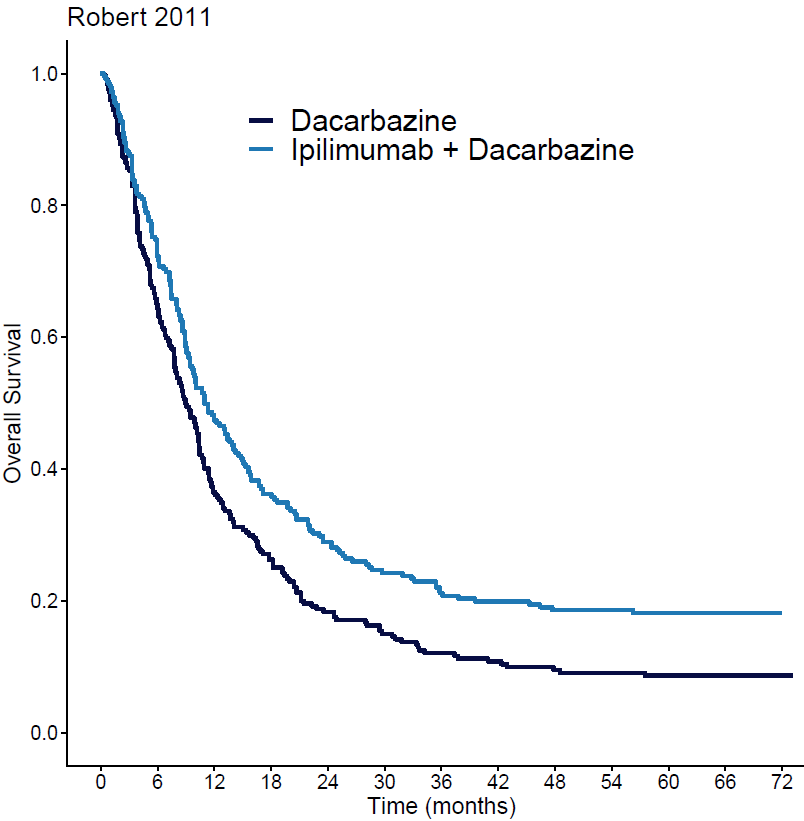


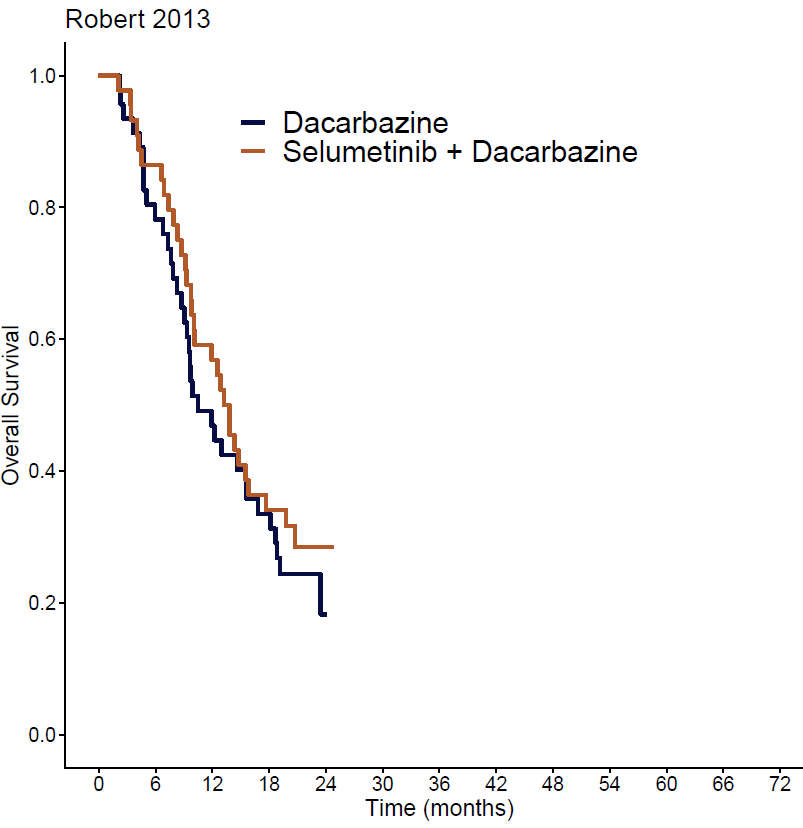


# Appendix C: Model implementation

Individual participant data dataset

The IPD dataset consists of seven columns. A snapshot of the data is included in Table C1. In Table C1:

- Patient ID is a sequential number within each arm of each trial
- Time is the survival time (or censoring time) for each patient
- Event is an indicator variable taking the value 1 if a patient died and 0 if they were censored
- Arm is a numerical variable taking the value 1, 2 or 3 to distinguish between treatment arms within a trial
- Study Code is a numerical identifier to distinguish between trials
- Study contains the name of the study and is often used for labelling trials within plots
- Tx Code is a numerical variable indicating which of the thirteen treatments a patient receives

Table C1. IPD Dataset

| **Patient ID** | **Time** | **Event** | **Arm** | **Study Code** | **Study** | **Tx Code** |
| --- | --- | --- | --- | --- | --- | --- |
| 1 | 1.518 | 1 | 1 | 1 | BREAK-3 | 1 |
| 2 | 2.889 | 1 | 1 | 1 | BREAK-3 | 1 |
| 3 | 3.035 | 1 | 1 | 1 | BREAK-3 | 1 |
| … | … | … | … | … | … | … |
| 44 | 0.881 | 0 | 1 | 1 | BREAK-3 | 1 |
| 45 | 1.689 | 0 | 1 | 1 | BREAK-3 | 1 |
| 46 | 2.595 | 0 | 1 | 1 | BREAK-3 | 1 |
| 1 | 0.089 | 1 | 2 | 1 | BREAK-3 | 4 |
| 2 | 1.449 | 1 | 2 | 1 | BREAK-3 | 4 |
| 3 | 2.186 | 1 | 2 | 1 | BREAK-3 | 4 |
| … | … | … | … | … | … | … |
| 132 | 0.971 | 0 | 2 | 1 | BREAK-3 | 4 |
| 133 | 1.575 | 0 | 2 | 1 | BREAK-3 | 4 |
| 134 | 2.491 | 0 | 2 | 1 | BREAK-3 | 4 |
| … | … | … | … | … | … | … |
| 43 | 22.736 | 0 | 2 | 13 | BREAK-3 | 13 |
| 44 | 23.443 | 0 | 2 | 13 | BREAK-3 | 13 |
| 45 | 24.792 | 0 | 2 | 13 | BREAK-3 | 13 |

Aggregate data dataset

For the fractional polynomial and piecewise exponential models the IPD is aggregated over time interval, treatment and trial so that the data takes the form of Table C2. In Table C2:

- spgrp is a numerical variable indicating the time interval, treatment contains the treatment label
- trialid contains the trial ID number
- y is the total time at risk
- nevents is the number of patients experiencing an event during the time interval
- natrisk is the total number of patients at risk during the time interval
- y.max is the maximum time for the interval
- start is the time at which the interval starts
- time is the time at which the interval ends

Table C2. Aggregated dataset for fractional polynomial and piecewise exponential models

| **spgrp** | **treatment** | **trial** | **y** | **nevents** | **natrisk** | **y.max** | **start** | **time** |
| --- | --- | --- | --- | --- | --- | --- | --- | --- |
| 1 | DB | 1 | 1056.18 | 28 | 187 | 6 | 0 | 6 |
| 1 | DB | 7 | 1177.82 | 34 | 211 | 6 | 0 | 6 |
| 1 | DB+TR | 7 | 1205.11 | 16 | 211 | 6 | 0 | 6 |
| … | … | … | … | … | … | … | … | … |
| 3 | VM | 6 | 1260.18 | 57 | 142 | 15.75 | 12 | 27.75 |
| 3 | VM | 8 | 319.27 | 15 | 122 | 8.13 | 12 | 20.13 |
| 3 | VM+COB | 6 | 1633.38 | 55 | 169 | 17.61 | 12 | 29.61 |

Choice of time intervals for aggregate data

Comparing models with differing time intervals is not straight forward, as the choice of time intervals cannot be guided by model fit statistics as the data to which the models are fit changes if we change the time intervals. It could be argued that time intervals should be:

1. Parsimonious and only included where needed due to (expected) changes in hazards (e.g. drug starts and stops working);
2. Chosen so that the number of events and patients at risk is roughly balanced. This would likely result in shorter time intervals at the start and longer time intervals towards the end;
3. Chosen to ensure that there are sufficient numbers of events per interval for the parameters to be estimated, which would justify fewer but longer time intervals. Against this requirement for sufficient data is the need to make realistic assumptions as it may not be justified that log hazard ratios and baseline hazards are fixed over longer time intervals. Therefore, the shape of the underlying hazard function plays an important role in determining the time intervals (Crowther2012).

In each trial, we identified the maximum follow-up time and across trials, we identified the trial with the shortest of these times (we call this the shortest maximum follow-up time). We based our choice of time intervals on the Kaplan-Meier plots of survival time but balanced this against the shortest maximum follow-up time to ensure we had at least five events in each arm of each trial for each interval. From the Kaplan-Meier plots of survival time for the dacarbazine trial arms, median survival time ranged from 9 to 15 months and the maximum follow-up times across trial arms ranged from 18 to 73 months. To ensure at least five events in each arm of each trial for each interval we used three intervals: 0-6 months, 6-12 months and >12 months.

Knot location for the Royston-Parmar model

Prior to fitting the Royston-Parmar model we had to define the restricted cubic spline functions for modelling the baseline log cumulative hazard for each trial. The quickest way to do this is to fit every trial with a restricted cubic spline in which the knots are placed at the same percentiles of the uncensored survival times. An alternative is to consider each trial individually. It has been shown that parameter estimates are generally robust to knot locations (Rutherford2015). Therefore, we fitted each trial with a restricted cubic spline with knots placed at the 33rd and 67th percentiles of uncensored survival times. A restricted cubic spline with two knots requires the calculation of three basis functions which are then transformed using Gram-Schmidt orthogonalisation. Full details on the calculation of basis functions can be found in (Freeman2017). R code for calculating and orthogonalising the basis functions is included in the files on GitHub.

Treatment contrast variables

The piecewise exponential and Royston-Parmar models model the treatment contrasts rather than arm-based data. Therefore, they require treatment contrast variables to identify which treatments are involved in the comparison. In a network of $q+1$ treatments, only $q$ treatment contrasts are defined. Treatment contrast variables can take the value 0, 1 or -1. For example, in the melanoma network, if we let $q$ represent the comparison between dacarbazine and dabrafenib then we can define ${trtq}_{i}=0$ if patient $i$ receives dacarbazine and ${trtq}_{i}=1$ if patient $i$ receives dabrafenib. In the presence of treatment loops, some care is required in defining the treatment contrast variables to ensure they are in the right direction. For example, in the melanoma network we have a treatment loop between ipilimumab, nivolumab and nivolumab plus ipilimumab. If we let ${trt1}_{i}$ represent the comparison between nivolumab and ipilimumab and ${trt2}_{i}$represent the comparison between nivolumab and nivolumab plus ipilimumab then, through the consistency equations we can obtain the treatment effect for ipilimumab versus nivolumab plus ipilimumab (i.e. ${trt2}_{i}- {trt1}_{i}$). In trials comparing nivolumab and ipilimumab we set ${trt1}_{i}=0$ if patient $i$ receives nivolumab (the baseline treatment for this comparison) and ${trt1}_{i}=1$ if patient $i$ receives ipilimumab (the comparator treatment for this comparison). In trials comparing nivolumab and nivolumab plus ipilimumab we set ${trt2}_{i}=0$ if patient $i$ receives nivolumab (the baseline treatment for this comparison) and ${trt2}_{i}=1$ if patient $i$ receives ipilimumab (the comparator treatment for this comparison). Then in trials comparing ipilimumab and nivolumab plus ipilimumab we set ${trt1}_{i}=0$ and ${trt2}_{i}=0$ if patient $i$ receives ipilimumab (the baseline treatment for this comparison) and ${trt1}_{i}=-1$ and ${trt2}_{i}=1$ if patient $i$ receives nivolumab plus ipilimumab (the comparator treatment for this comparison). In doing so we ensure that the network is properly defined (Freeman2017).

**References**

Crowther MJ, Riley RD, Staessen JA, Wang J, Gueyffier F, Lambert PC. Individual patient data meta-analysis of survival data using Poisson regression models. *BMC Medical Research Methodology*, 2012: 12; 34

Freeman SC, Carpenter JR. Bayesian one-step IPD network meta-analysis of time-to-event data using Royston-Parmar models. *Research Synthesis Methods*, 2017: 8; 451-464

Rutherford MJ, Crowther MJ, Lambert PC. The use of restricted cubic splines to approximate complex hazard functions in the analysis of time‐to‐event data: A simulation study. *Journal of Statistical Computation and Simulation*, 2015; 85: 777-793

# Appendix D: Log-Log plots for assessing the proportional hazards assumption

Schoenfeld test p = 0.88

Schoenfeld test p <0.001

Schoenfeld test p = 0.65

Schoenfeld test p = 0.006

Schoenfeld test p = 0.19

Schoenfeld test p = 0.40

Schoenfeld test p = 0.31

Schoenfeld test p = 0.50

Schoenfeld test p = 0.75

Schoenfeld test p = 0.011

Schoenfeld test p = 0.48

Schoenfeld test p = 0.71

Schoenfeld test p = 0.75

# Appendix E: Kaplan-Meier plot of dacarbazine trial arms


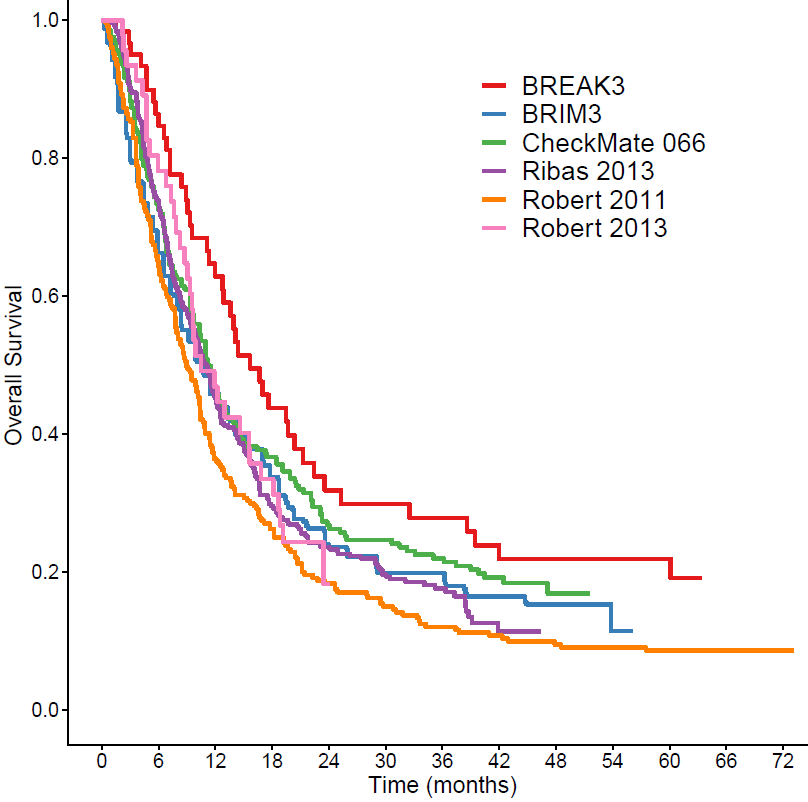


# Appendix F: Tables of parameter estimates

Table F1: Log hazard ratios from the fixed effect NMA model

| **Treatment** | **Log Hazard Ratio (95% Credible Interval)** |
| --- | --- |
| Tremelimumab | -0.13 (-0.30, 0.04) |
| Ipilimumab + Dacarbazine | -0.32 (-0.51, -0.12) |
| Dabrafenib | -0.23 (-0.50, 0.03) |
| Dabrafenib + Trametinib | -0.53 (-0.79, -0.28) |
| Vemurafenib | -0.22 (-0.38, -0.05) |
| Vemurafenib + Cobimetinib | -0.59 (-0.89, -0.30) |
| Ipilimumab | -0.36 (-0.68, -0.04) |
| Nivolumab | -0.77 (-1.02, -0.52) |
| Nivolumab + Ipilimumab | -0.93 (-1.30, -0.55) |
| Pembrolizumab | -0.68 (-1.04, -0.31) |
| Ipilimumab + Sargramostin | -0.77 (-1.27, -0.26) |
| Selumetinib + Dacarbazine | -0.19 (-0.67, 0.29) |

Table F2: Restricted mean survival time at 18 months from the fixed effect NMA model

| **Treatment** | **RMST (95% Credible Interval)** |
| --- | --- |
| Tremelimumab | 0.55 (-0.38, 1.47) |
| Ipilimumab + Dacarbazine | 1.41 (0.34, 2.48) |
| Dabrafenib | 1.31 (0.19, 2.44) |
| Dabrafenib + Trametinib | 2.72 (1.69, 3.75) |
| Vemurafenib | 1.75 (0.91, 2.61) |
| Vemurafenib + Cobimetinib | 3.16 (1.89, 4.43) |
| Ipilimumab | 2.47 (1.19, 3.77) |
| Nivolumab | 2.89 (1.72, 4.08) |
| Nivolumab + Ipilimumab | 2.89 (1.64, 4.17) |
| Pembrolizumab | 4.32 (2.77, 5.91) |
| Ipilimumab + Sargramostin | 4.23 (2.13, 6.31) |
| Selumetinib + Dacarbazine | 0.85 (-1.38, 3.09) |

Table F3: Parameter estimates from the fixed effect generalised gamma model with treatment as a location parameter

| **Parameter** | **Median (95% Credible Interval)** |
| --- | --- |
| Alpha: Tremelimumab | 0.05 (-0.12, 0.21) |
| Alpha: Ipilimumab + Dacarbazine | 0.30 (0.07, 0.53) |
| Alpha: Dabrafenib | 0.30 (0.03, 0.56) |
| Alpha: Dabrafenib + Trametinib | 0.67 (0.43, 0.90) |
| Alpha: Vemurafenib | 0.46 (0.27, 0.66) |
| Alpha: Vemurafenib + Cobimetinib | 0.78 (0.50, 1.06) |
| Alpha: Ipilimumab | 0.43 (0, 0.87) |
| Alpha: Nivolumab | 0.85 (0.53, 1.16) |
| Alpha: Nivolumab + Ipilimumab | 0.96 (0.53, 1.40) |
| Alpha: Pembrolizumab | 0.94 (0.43, 1.45) |
| Alpha: Ipilimumab + Sargramostin | 0.80 (0.21, 1.39) |
| Alpha: Selumetinib + Dacarbazine | 0.16 (-0.20, 0.51) |

Table F4: Parameter estimates from the fixed effect piecewise exponential model with cut point at 12 months

| **Parameter** | **Median (95% Credible Interval)** |
| --- | --- |
| Alpha: Tremelimumab | -0.17 (-0.38, 0.05) |
| Alpha: Ipilimumab + Dacarbazine | -0.29 (-0.52, -0.06) |
| Alpha: Dabrafenib | -0.21 (-0.70, 0.33) |
| Alpha: Dabrafenib + Trametinib | 0.19 (-0.07, 0.44) |
| Alpha: Vemurafenib | -0.19 (-0.39, 0.01) |
| Alpha: Vemurafenib + Cobimetinib | -0.45 (-0.79, -0.12) |
| Alpha: Ipilimumab | -0.31 (-0.60, -0.02) |
| Alpha: Nivolumab | -0.77(-1.10, -0.46) |
| Alpha: Nivolumab + Ipilimumab | -0.25 (-0.51, 0.01) |
| Alpha: Pembrolizumab | -0.47 (-0.71, -0.21) |
| Alpha: Ipilimumab + Sargramostin | -0.50 (-0.92, -0.08) |
| Alpha: Selumetinib + Dacarbazine | -0.31 (-0.92, 0.31) |
| Phi: Tremelimumab | 0.08 (-0.27, 0.44) |
| Phi: Ipilimumab + Dacarbazine | -0.16 (-0.57, 0.25) |
| Phi: Dabrafenib | 0.04 (-0.66, 0.74) |
| Phi: Dabrafenib + Trametinib | -0.36 (-0.76, 0.05) |
| Phi: Vemurafenib | 0.30 (-0.02, 0.63) |
| Phi: Vemurafenib + Cobimetinib | 0.16 (-0.34, 0.66) |
| Phi: Ipilimumab | -0.38 (-0.78, 0.02) |
| Phi: Nivolumab | -0.04 (-0.54, 0.45) |
| Phi: Nivolumab + Ipilimumab | -0.87 (-1.27, -0.48) |
| Phi: Pembrolizumab | 0.37 (0, 0.75) |
| Phi: Ipilimumab + Sargramostin | 0.85 (-0.37, 2.12) |
| Phi: Selumetinib + Dacarbazine | 0.34 (-0.70, 1.40) |

Table F5: Parameter estimates from the fixed effect fractional polynomial model with p=0

| **Parameter** | **Median (95% Credible Interval)** |
| --- | --- |
| d[2,1] Tremelimumab | 0.04 (-0.37, 0.45) |
| d[2,2] Tremelimumab | -0.08 (-0.24, 0.08) |
| d[3,1] Ipilimumab + Dacarbazine | -0.15 (-0.55, 0.24) |
| d[3,2] Ipilimumab + Dacarbazine | -0.08 (-0.23, 0.07) |
| d[4,1] Dabrafenib | -0.34 (-0.97, 0.40) |
| d[4,2] Dabrafenib | 0.04 (-0.23, 0.27) |
| d[5,1] Dabrafenib + Trametinib | -0.99 (-1.68, -0.29) |
| d[5,2] Dabrafenib + Trametinib | 0.18 (-0.10, 0.46) |
| d[6,1] Vemurafenib | -0.73 (-1.12, -0.34) |
| d[6,2] Vemurafenib | 0.22 (0.07, 0.36) |
| d[7,1] Vemurafenib + Cobimetinib | -1.33 (-2.31, -0.39) |
| d[7,2] Vemurafenib + Cobimetinib | 0.29 (-0.07, 0.67) |
| d[8,1] Ipilimumab | -0.30 (-1.05, 0.53) |
| d[8,2] Ipilimumab | -0.06 (-0.35, 0.21) |
| d[9,1] Nivolumab | -0.51 (-1.10, 0.11) |
| d[9,2] Nivolumab | -0.12 (-0.35, 0.10) |
| d[10,1] Nivolumab + Ipilimumab | -0.30 (-1.05, 0.53) |
| d[10,2] Nivolumab + Ipilimumab | -0.27 (-0.56, 0) |
| d[11,1] Pembrolizumab | -0.88 (-1.75, 0.06) |
| d[11,2] Pembrolizumab | 0.05 (-0.28, 0.37) |
| d[12,1] Ipilimumab + Sargramostin | -0.73 (-2.03, 0.60) |
| d[12,2] Ipilimumab + Sargramostin | -0.06 (-0.66, 0.54) |
| d[13,1] Selumetinib + Dacarbazine | -0.40 (-2.10, 1.10) |
| d[13,2] Selumetinib + Dacarbazine | 0.08 (-0.54, 0.76) |

Table F6: Parameter estimates from the fixed effect Royston-Parmar model including treatment-ln(time) interactions

| **Parameter** | **Median (95% Credible Interval)** |
| --- | --- |
| Alpha: Tremelimumab | 0.05 (-0.43, 0.52) |
| Alpha: Ipilimumab + Dacarbazine | -0.20 (-0.63, 0.23) |
| Alpha: Dabrafenib | -0.22 (-1.10, 0.80) |
| Alpha: Dabrafenib + Trametinib | 0.57 (0, 1.14) |
| Alpha: Vemurafenib | -0.74 (-1.16, -0.34) |
| Alpha: Vemurafenib + Cobimetinib | -0.91 (-1.87, 0) |
| Alpha: Ipilimumab | -0.04 (-0.57, 0.50) |
| Alpha: Nivolumab | -0.61 (-1.24, 0.01) |
| Alpha: Nivolumab + Ipilimumab | 0.24 (-0.25, 0.72) |
| Alpha: Pembrolizumab | -0.85 (-1.30, -0.42) |
| Alpha: Ipilimumab + Sargramostin | -0.50 (-1.56, 0.49) |
| Alpha: Selumetinib + Dacarbazine | -0.49 (-2.40, 1.32) |
| Alpha: Tremelimumab*ln(t) | -0.06 (-0.20, 0.09) |
| Alpha: Ipilimumab + Dacarbazine*ln(t) | -0.04 (-0.16, 0.08) |
| Alpha: Dabrafenib*ln(t) | 0.01 (-0.27, 0.25) |
| Alpha: Dabrafenib + Trametinib*ln(t) | -0.16 (-0.33, 0.02) |
| Alpha: Vemurafenib*ln(t) | 0.21 (0.09, 0.34) |
| Alpha: Vemurafenib + Cobimetinib*ln(t) | 0.18 (-0.12, 0.48) |
| Alpha: Ipilimumab*ln(t) | -0.11 (-0.25, 0.03) |
| Alpha: Nivolumab*ln(t) | -0.05 (-0.22, 0.13) |
| Alpha: Nivolumab + Ipilimumab*ln(t) | -0.23 (-0.36, -0.11) |
| Alpha: Pembrolizumab*ln(t) | 0.15 (0.04, 0.27) |
| Alpha: Ipilimumab + Sargramostin*ln(t) | 0.04 (-0.33, 0.44) |
| Alpha: Selumetinib + Dacarbazine*ln(t) | 0.11 (-0.51, 0.76) |

# Appendix G: Probability of each treatment obtaining each rank from 1 to 13


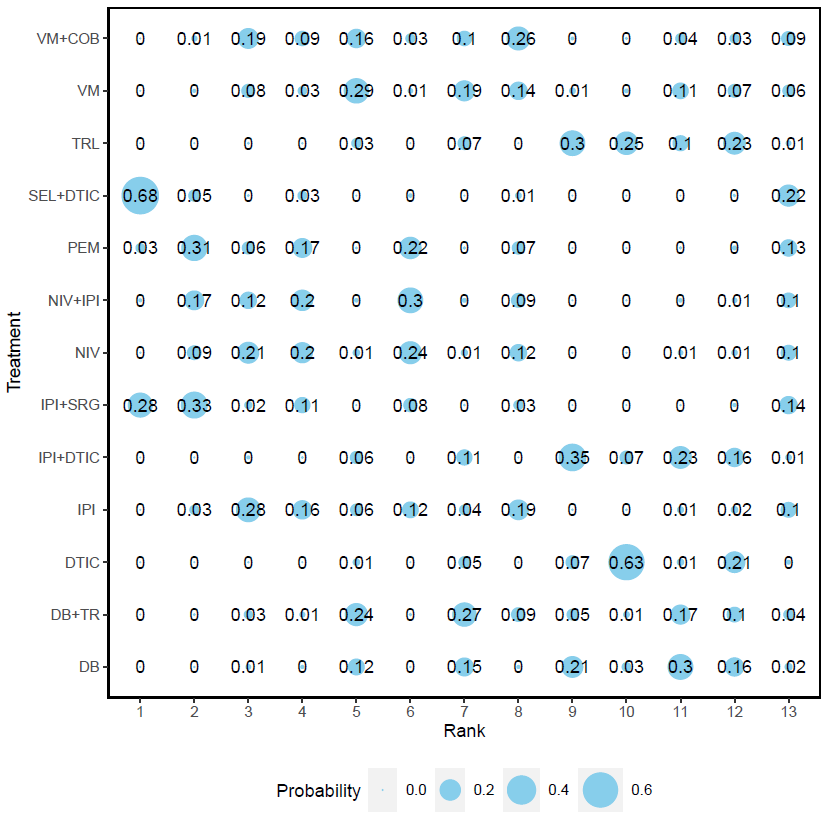


Figure G1: Probability of each treatment obtaining each rank from 1 to 13 for the fixed effect Cox PH model. COB = Cobimetinib, DB = Dabrafenib, DTIC = Dacarbazine, IPI = Ipilimumab, NIV = Nivolumab, PEM = Pembrolizumab, SEL = Selumetinib, SRG = Sargramostin, TR = Trametinib, TRL = Tremelimumab, VM = Vemurafenib.


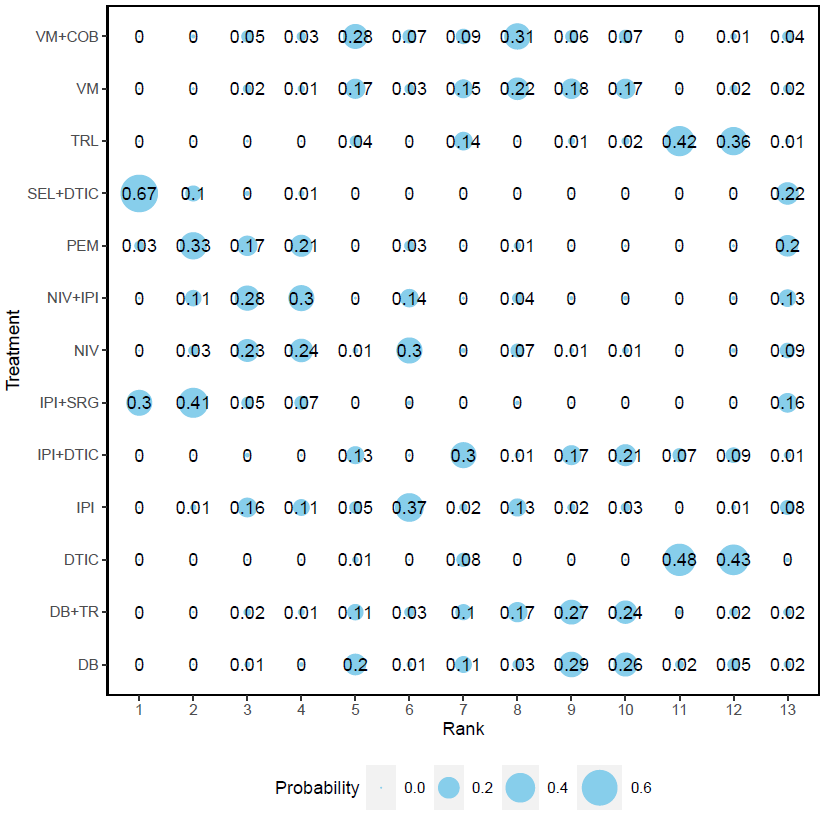


Figure G2: Probability of each treatment obtaining each rank from 1 to 13 for the fixed effect restricted mean survival time model. COB = Cobimetinib, DB = Dabrafenib, DTIC = Dacarbazine, IPI = Ipilimumab, NIV = Nivolumab, PEM = Pembrolizumab, SEL = Selumetinib, SRG = Sargramostin, TR = Trametinib, TRL = Tremelimumab, VM = Vemurafenib.


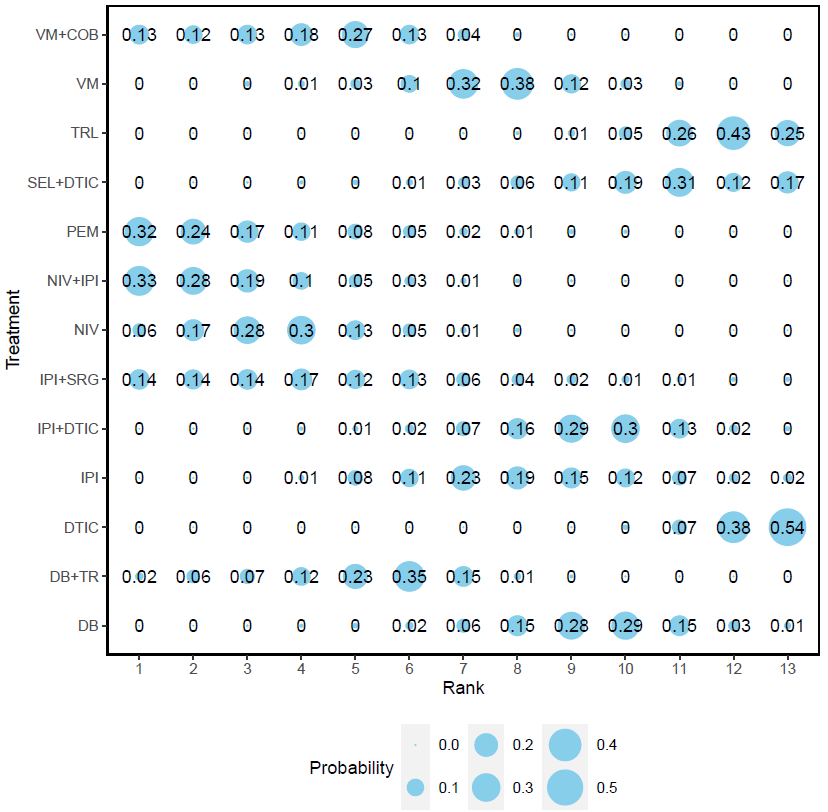


Figure G3: Probability of each treatment obtaining each rank from 1 to 13 for the fixed effect generalised gamma model including treatment as a location parameter. COB = Cobimetinib, DB = Dabrafenib, DTIC = Dacarbazine, IPI = Ipilimumab, NIV = Nivolumab, PEM = Pembrolizumab, SEL = Selumetinib, SRG = Sargramostin, TR = Trametinib, TRL = Tremelimumab, VM = Vemurafenib.


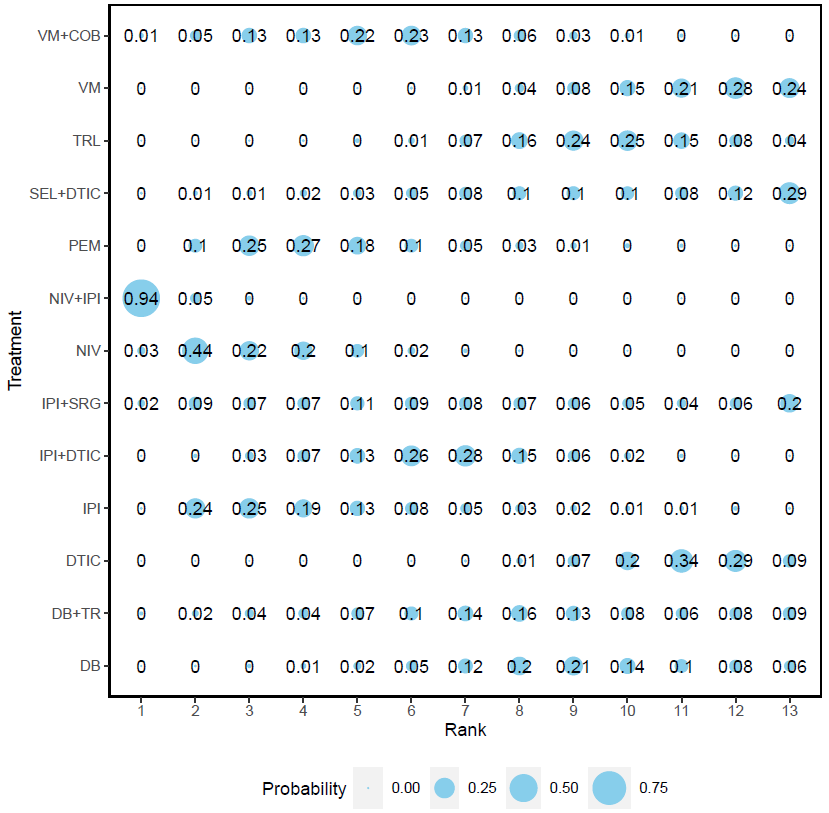


Figure G4: Probability of each treatment obtaining each rank from 1 to 13 for the fixed effect piecewise exponential model with a cut point at twelve months. COB = Cobimetinib, DB = Dabrafenib, DTIC = Dacarbazine, IPI = Ipilimumab, NIV = Nivolumab, PEM = Pembrolizumab, SEL = Selumetinib, SRG = Sargramostin, TR = Trametinib, TRL = Tremelimumab, VM = Vemurafenib.


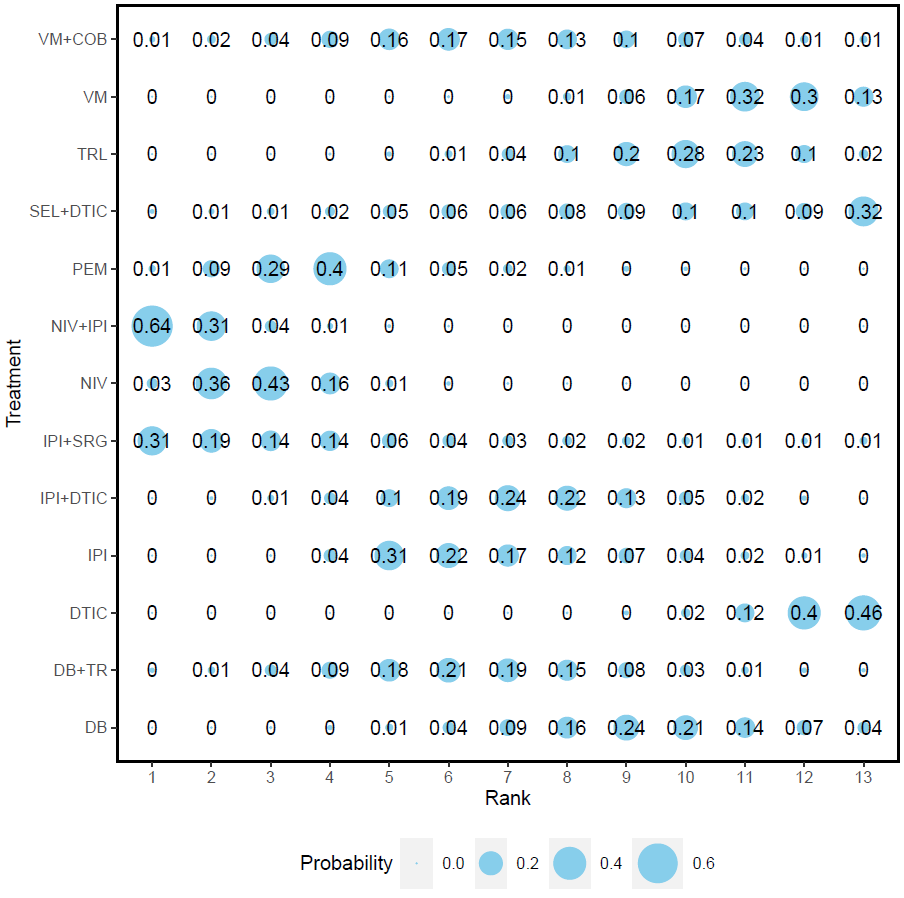


Figure G5: Probability of each treatment obtaining each rank from 1 to 13 for the fixed effect fractional polynomial model with p=0. COB = Cobimetinib, DB = Dabrafenib, DTIC = Dacarbazine, IPI = Ipilimumab, NIV = Nivolumab, PEM = Pembrolizumab, SEL = Selumetinib, SRG = Sargramostin, TR = Trametinib, TRL = Tremelimumab, VM = Vemurafenib.


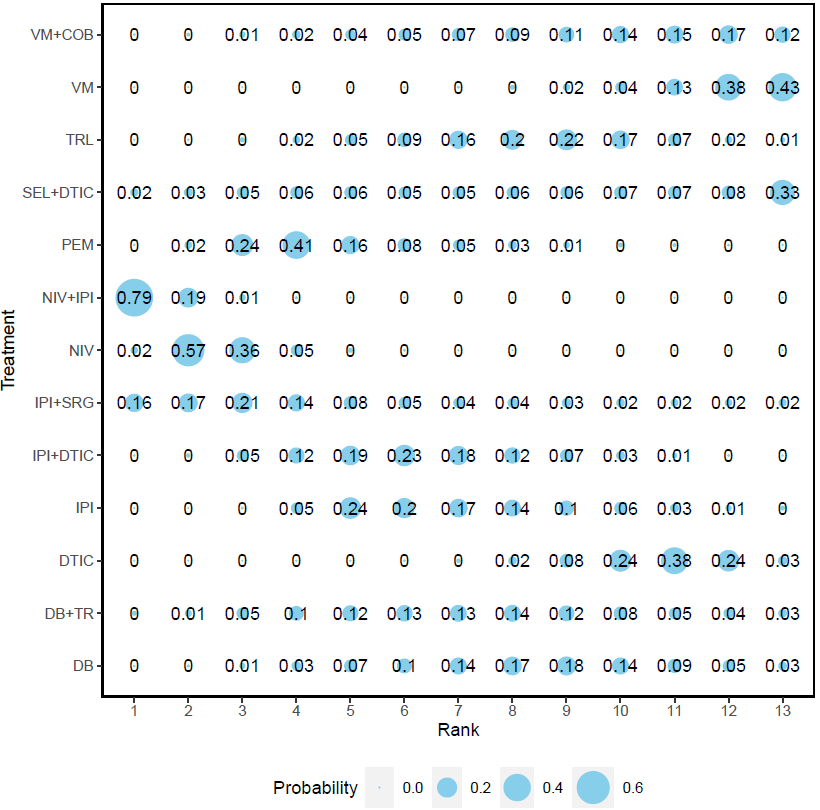


Figure G6: Probability of each treatment obtaining each rank from 1 to 13 for the fixed effect Royston-Parmar non-PH model. COB = Cobimetinib, DB = Dabrafenib, DTIC = Dacarbazine, IPI = Ipilimumab, NIV = Nivolumab, PEM = Pembrolizumab, SEL = Selumetinib, SRG = Sargramostin, TR = Trametinib, TRL = Tremelimumab, VM = Vemurafenib.

# Appendix H: Survival curves from piecewise exponential models


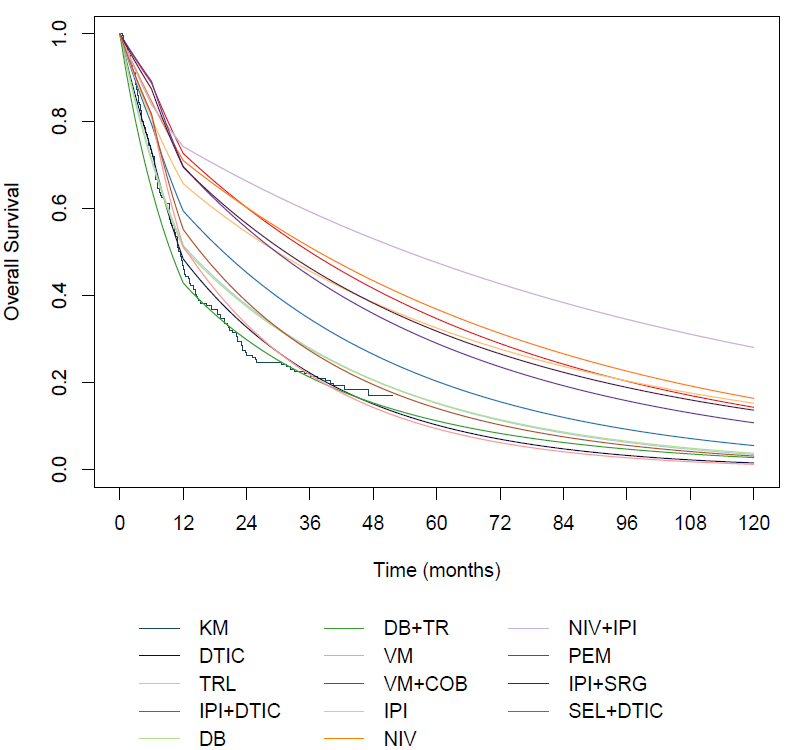


Figure H1: Survival curves from the fixed effect piecewise Poisson model with cut point at six months. COB = Cobimetinib, DB = Dabrafenib, DTIC = Dacarbazine, IPI = Ipilimumab, NIV = Nivolumab, PEM = Pembrolizumab, SEL = Selumetinib, SRG = Sargramostin, TR = Trametinib, TRL = Tremelimumab, VM = Vemurafenib.


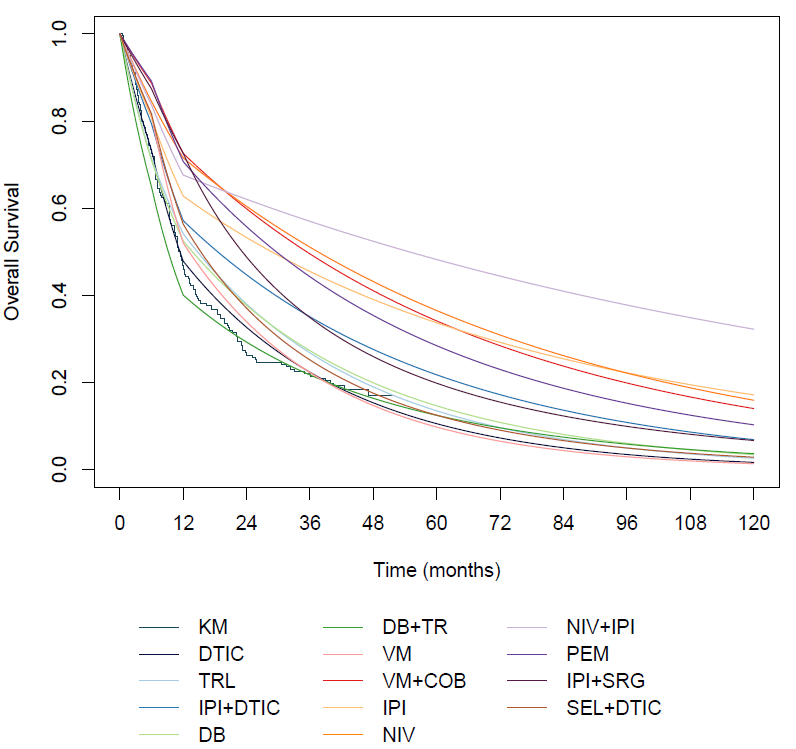


Figure H2: Survival curves from the fixed effect piecewise Poisson model with cut points at six and twelve months. COB = Cobimetinib, DB = Dabrafenib, DTIC = Dacarbazine, IPI = Ipilimumab, NIV = Nivolumab, PEM = Pembrolizumab, SEL = Selumetinib, SRG = Sargramostin, TR = Trametinib, TRL = Tremelimumab, VM = Vemurafenib.

# Appendix I: Fractional polynomial models


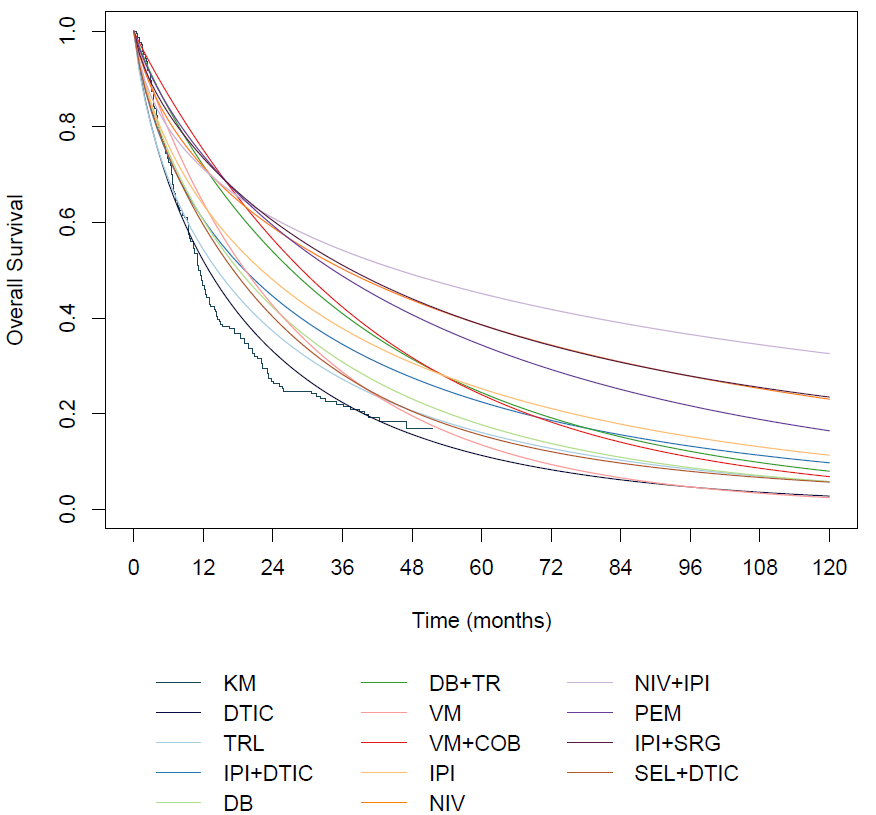


Figure I1: Survival curves from first order fixed effect fractional polynomial model with p=0.5. KM = Kaplan-Meier, COB = Cobimetinib, DB = Dabrafenib, DTIC = Dacarbazine, IPI = Ipilimumab, NIV = Nivolumab, PEM = Pembrolizumab, SEL = Selumetinib, SRG = Sargramostin, TR = Trametinib, TRL = Tremelimumab, VM = Vemurafenib.


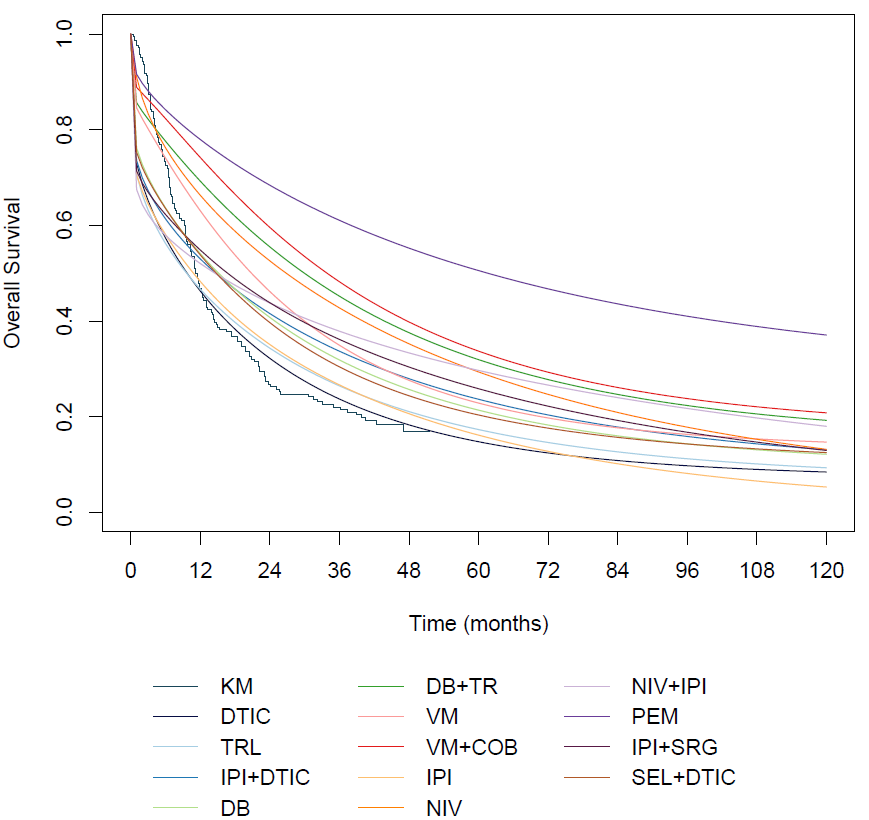


Figure I2: Survival curves from first order fixed effect fractional polynomial model with p=-0.5. KM = Kaplan-Meier, COB = Cobimetinib, DB = Dabrafenib, DTIC = Dacarbazine, IPI = Ipilimumab, NIV = Nivolumab, PEM = Pembrolizumab, SEL = Selumetinib, SRG = Sargramostin, TR = Trametinib, TRL = Tremelimumab, VM = Vemurafenib.


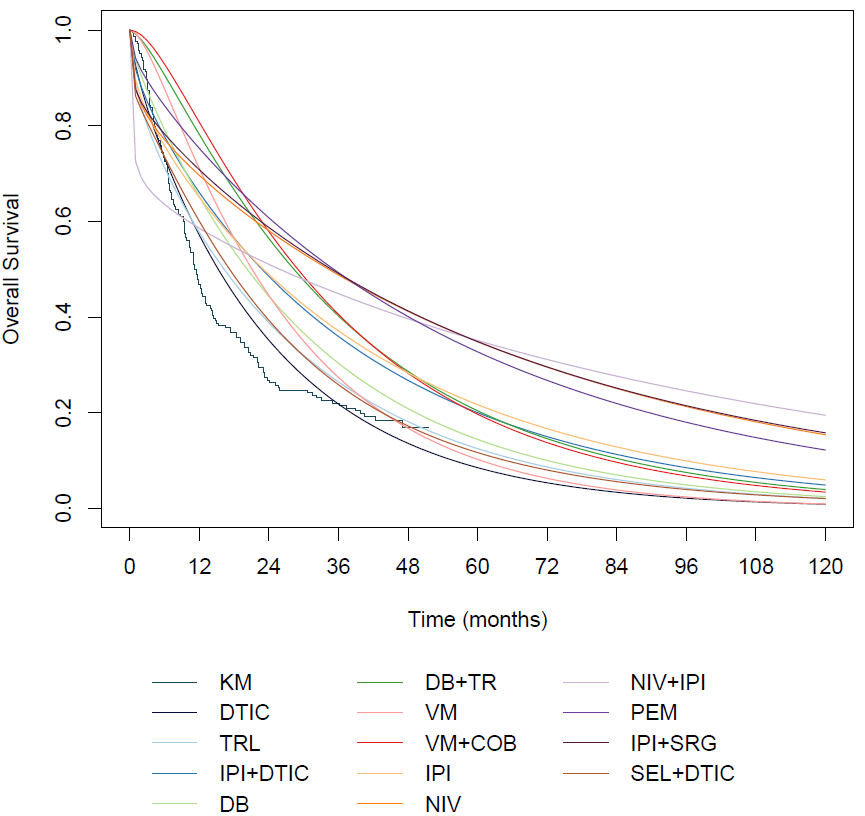


Figure I3: Survival curves from first order fixed effect fractional polynomial model with p=-1. KM = Kaplan-Meier, COB = Cobimetinib, DB = Dabrafenib, DTIC = Dacarbazine, IPI = Ipilimumab, NIV = Nivolumab, PEM = Pembrolizumab, SEL = Selumetinib, SRG = Sargramostin, TR = Trametinib, TRL = Tremelimumab, VM = Vemurafenib.


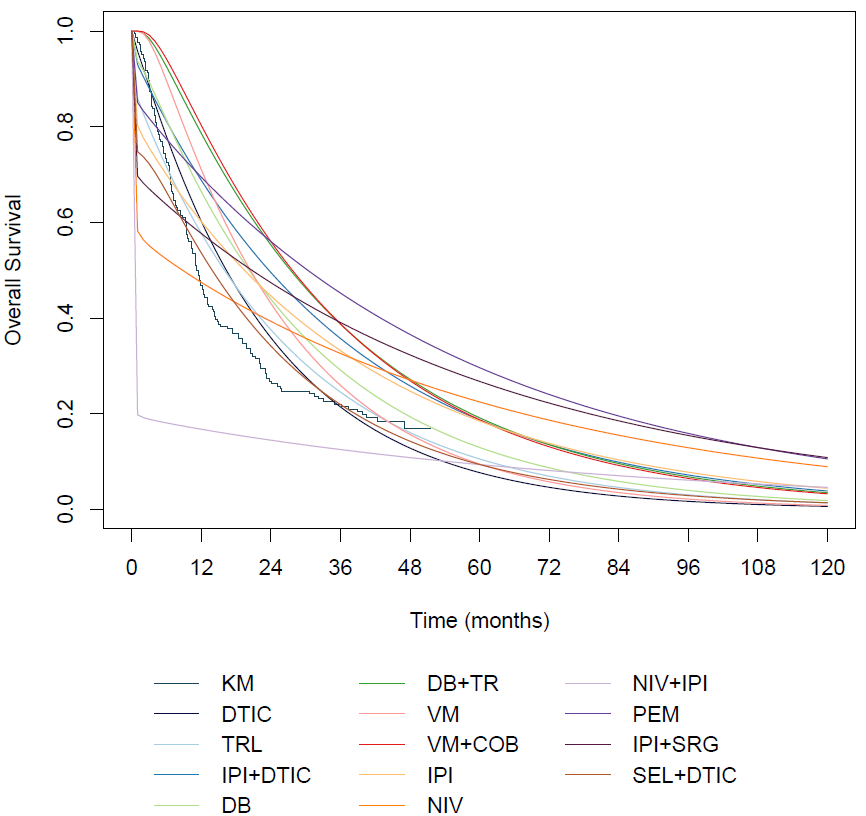


Figure I4: Survival curves from first order fixed effect fractional polynomial model with p=-2. KM = Kaplan-Meier, COB = Cobimetinib, DB = Dabrafenib, DTIC = Dacarbazine, IPI = Ipilimumab, NIV = Nivolumab, PEM = Pembrolizumab, SEL = Selumetinib, SRG = Sargramostin, TR = Trametinib, TRL = Tremelimumab, VM = Vemurafenib.

Table I1. Deviance information criteria (DIC) from first order fractional polynomial models

| **Model** | **DIC** |
| --- | --- |
| p=-2 | 2338.19 |
| p=-1 | 2320.61 |
| p=-0.5 | 2219.34 |
| p=0 | 2030.84 |
| p=0.5 | 2031.26 |

# Appendix J: Improvement in restricted mean survival time at 60 months


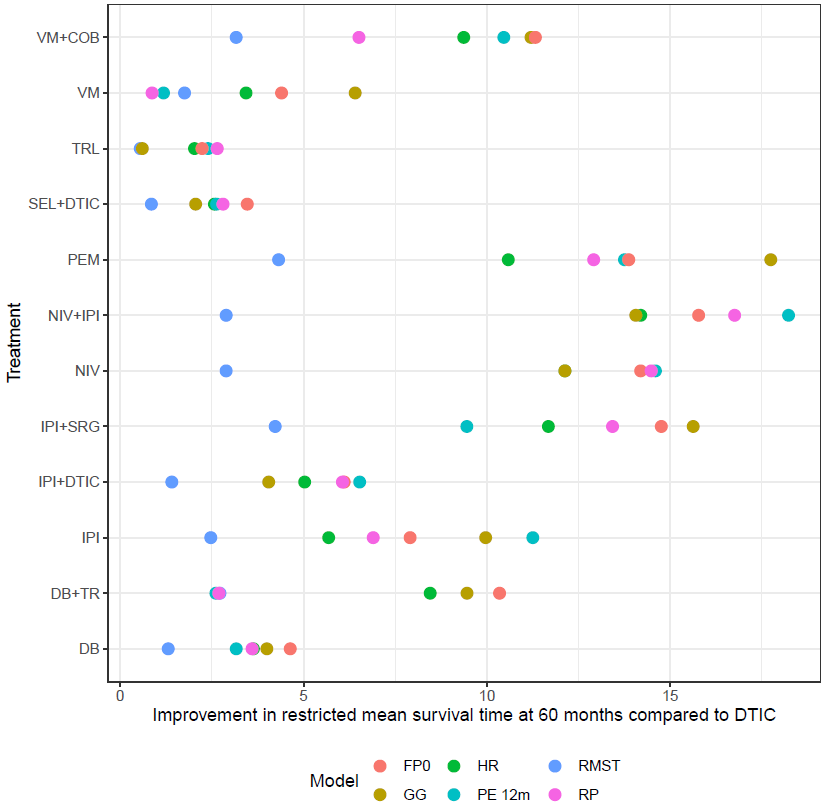


Figure J1: Improvement in restricted mean survival time at 60 months compared to dacarbazine from the generalised gamma, piecewise exponential, fractional polynomial and Royston-Parmar models. Improvement in restricted mean survival time at 18 months from the RMST model and at 51.5 months from the hazard ratio model. FP0 = fractional polynomial with p=0, GG = generalised gamma model with treatment modelled as a location parameter, HR = Cox proportional hazards model, PE 12m = piecewise exponential model with cut point at 12 months, RMST = restricted mean survival time, RP = Royston-Parmar non-proportional hazards model. COB = Cobimetinib, DB = Dabrafenib, DTIC = Dacarbazine, IPI = Ipilimumab, NIV = Nivolumab, PEM = Pembrolizumab, SEL = Selumetinib, SRG = Sargramostin, TR = Trametinib, TRL = Tremelimumab, VM = Vemurafenib.

# Appendix K: Model fit versus observed data plots


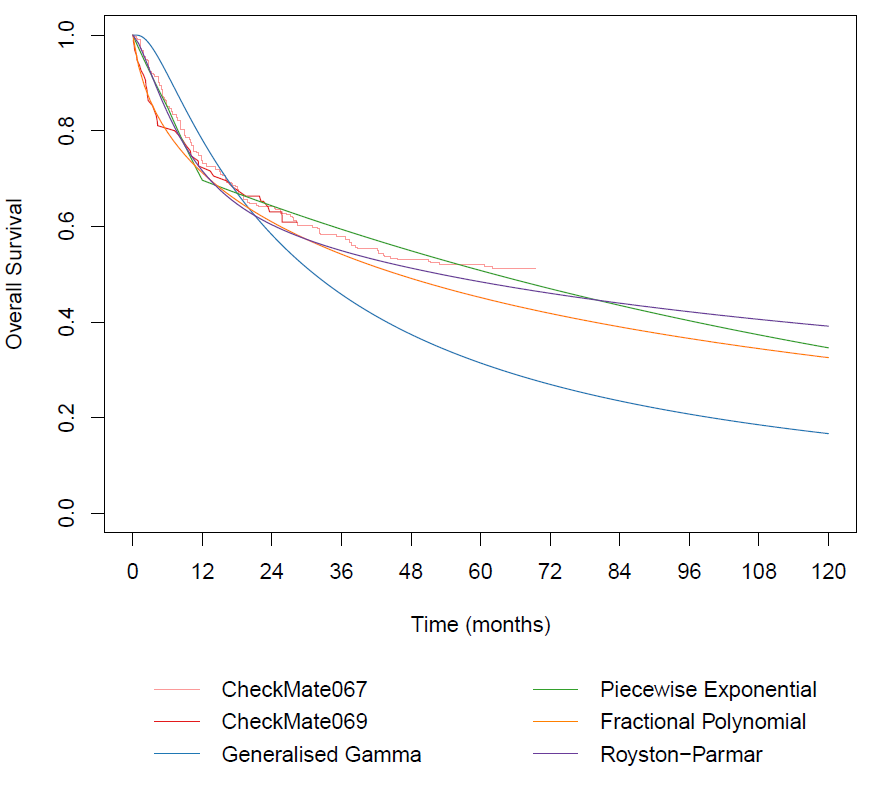


Figure K1: Kaplan-Meier survival curves for CheckMate 067 and CheckMate 069 trials alongside survival estimates from the generalised gamma model with treatment as a location parameter, the piecewise exponential model with a cut point at 12 months, the fractional polynomial model with p=0 and the Royston-Parmar model with treatment-ln(time) interactions for nivolumab plus ipilimumab.


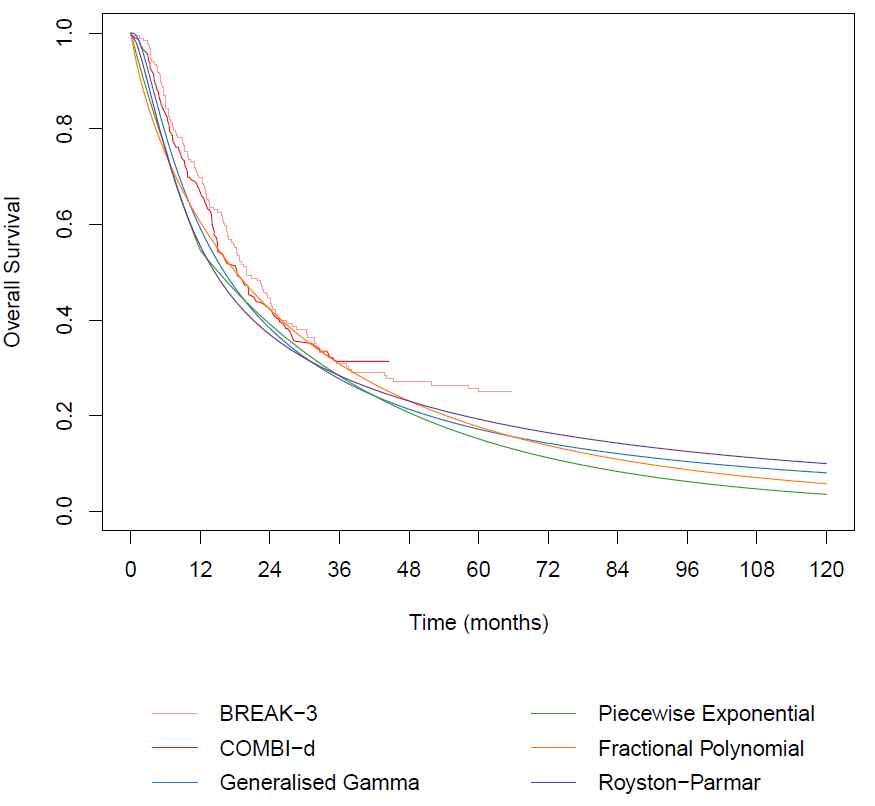
Figure K2: Kaplan-Meier survival curves for BREAK-3 and COMBI-d trials alongside survival estimates from the generalised gamma model with treatment as a location parameter, the piecewise exponential model with a cut point at 12 months, the fractional polynomial model with p=0 and the Royston-Parmar model with treatment-ln(time) interactions for dabrafenib.


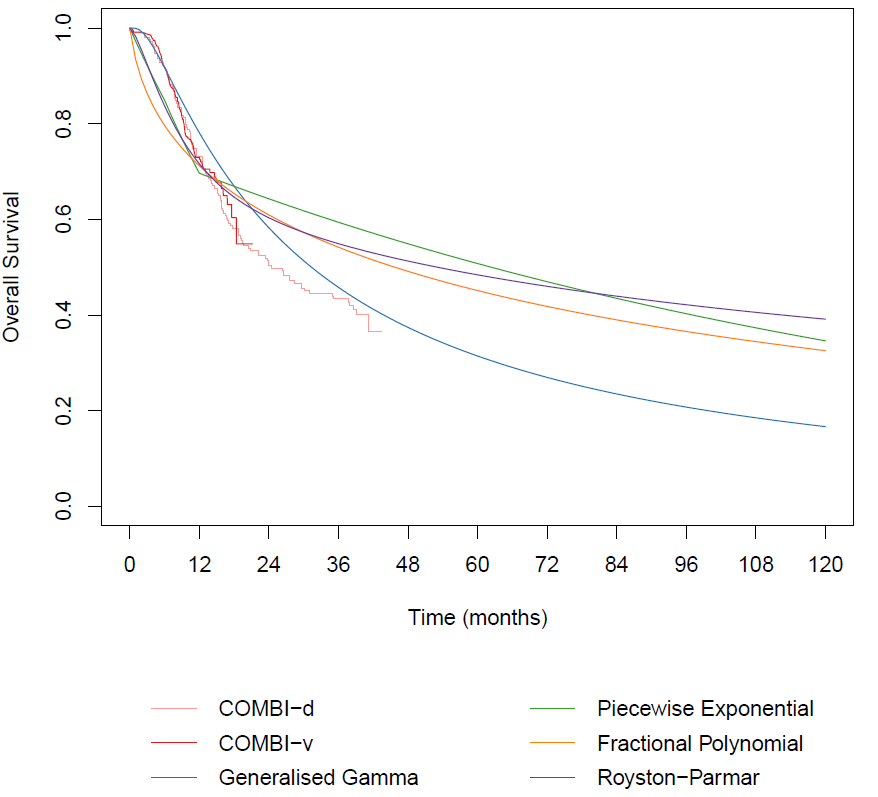


Figure K3: Kaplan-Meier survival curves for COMBI-d and COMBI-v trials alongside survival estimates from the generalised gamma model with treatment as a location parameter, the piecewise exponential model with a cut point at 12 months, the fractional polynomial model with p=0 and the Royston-Parmar model with treatment-ln(time) interactions for dabrafenib plus trametinib.


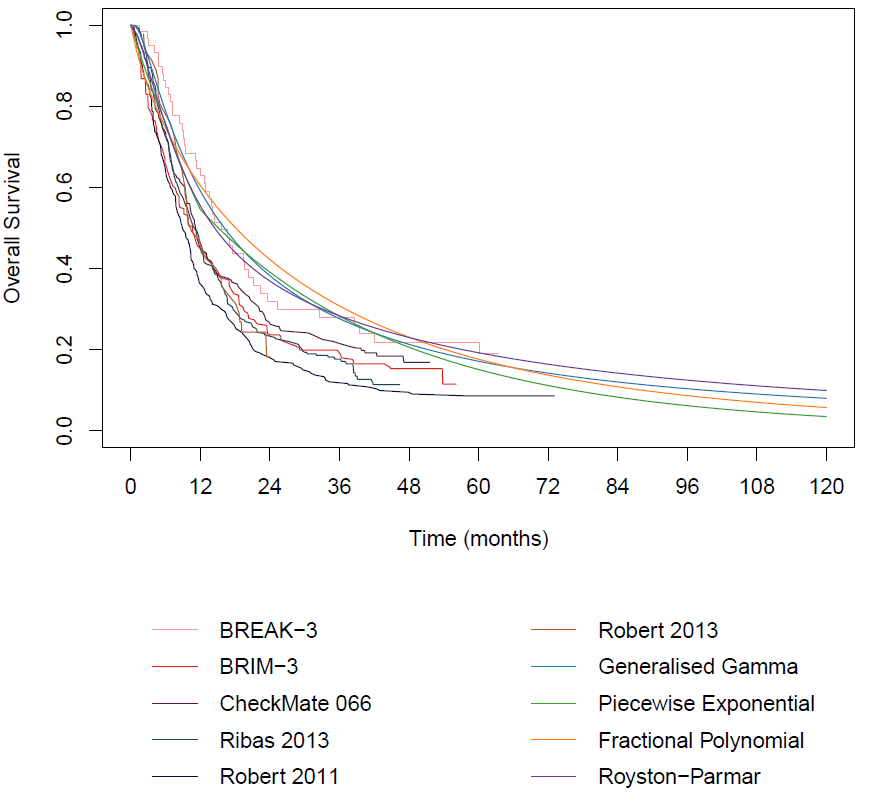


Figure K4: Kaplan-Meier survival curves for BREAK-3, BRIM-3, CheckMate 066, Ribas 2013, Robert 2011 and Robert 2013 trials alongside survival estimates from the generalised gamma model with treatment as a location parameter, the piecewise exponential model with a cut point at 12 months, the fractional polynomial model with p=0 and the Royston-Parmar model with treatment-ln(time) interactions for dacarbazine.


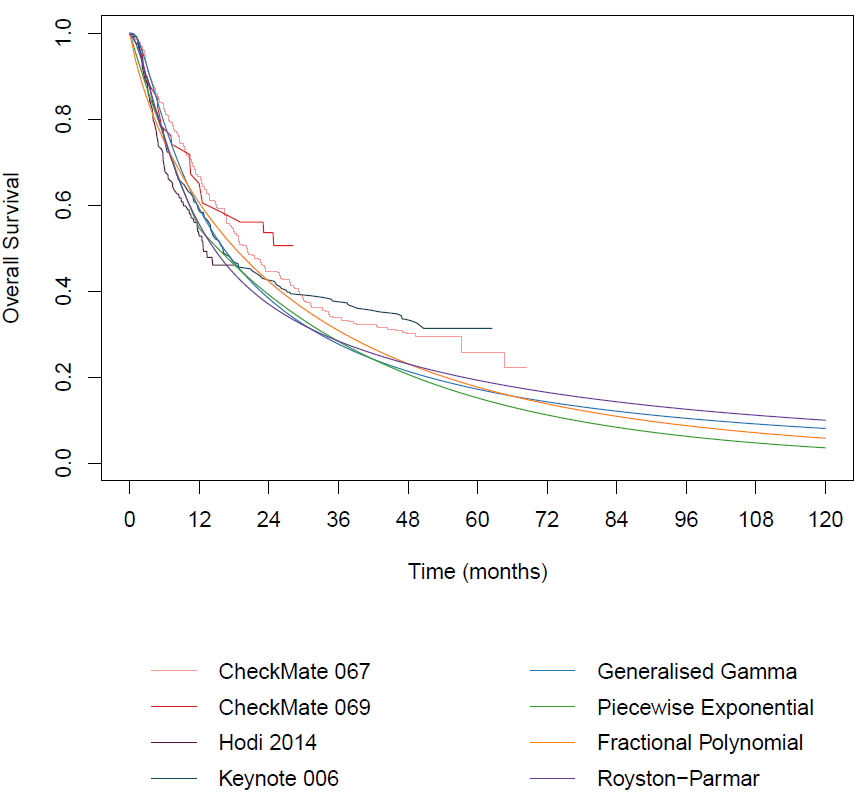


Figure K5: Kaplan-Meier survival curves for CheckMate 067, ChecKmate 069, Hodi 2014 and Keynote 006 trials alongside survival estimates from the generalised gamma model with treatment as a location parameter, the piecewise exponential model with a cut point at 12 months, the fractional polynomial model with p=0 and the Royston-Parmar model with treatment-ln(time) interactions for ipilimumab.


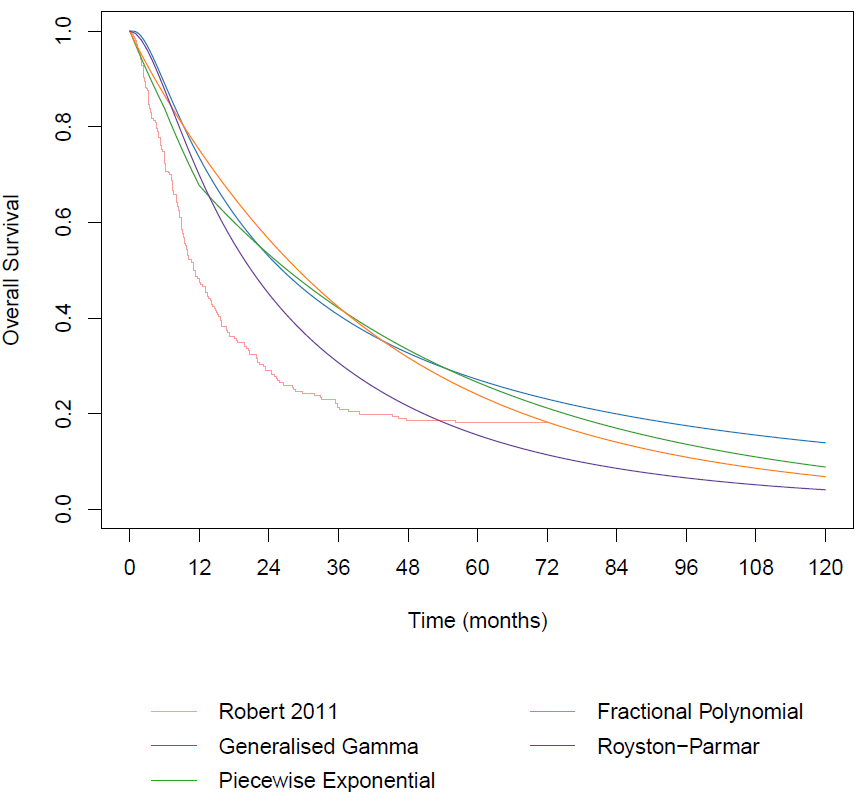


Figure K6: Kaplan-Meier survival curve for the Robert 2011 trial alongside survival estimates from the generalised gamma model with treatment as a location parameter, the piecewise exponential model with a cut point at 12 months, the fractional polynomial model with p=0 and the Royston-Parmar model with treatment-ln(time) interactions for ipilimumab plus dacarbazine.


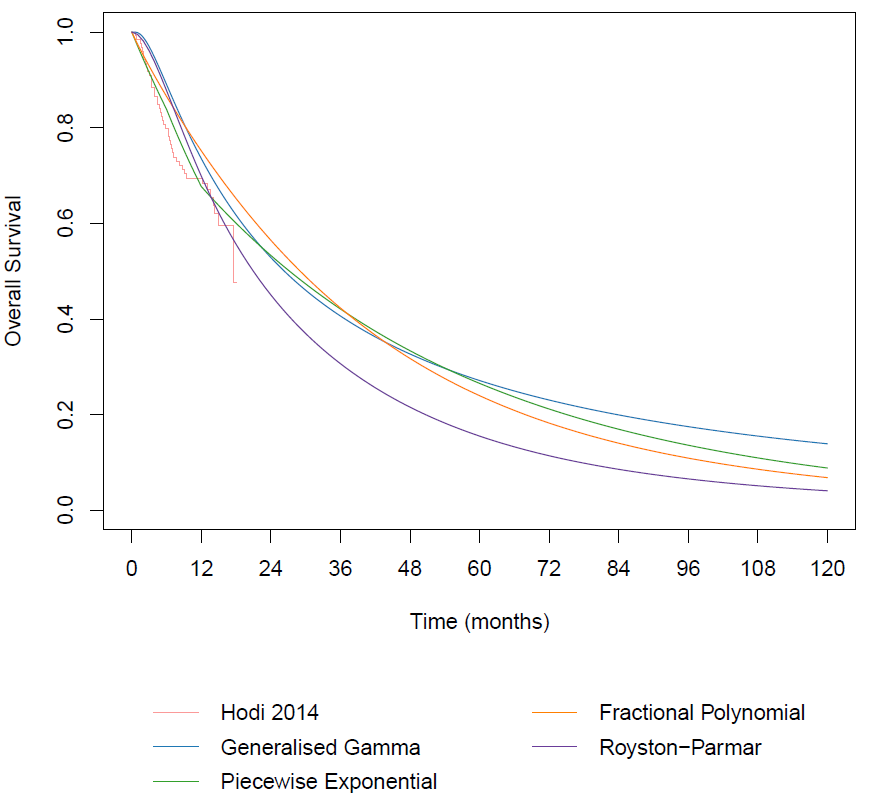


Figure K7: Kaplan-Meier survival curve for the Hodi 2014 trial alongside survival estimates from the generalised gamma model with treatment as a location parameter, the piecewise exponential model with a cut point at 12 months, the fractional polynomial model with p=0 and the Royston-Parmar model with treatment-ln(time) interactions for ipilimumab plus sargramostin.


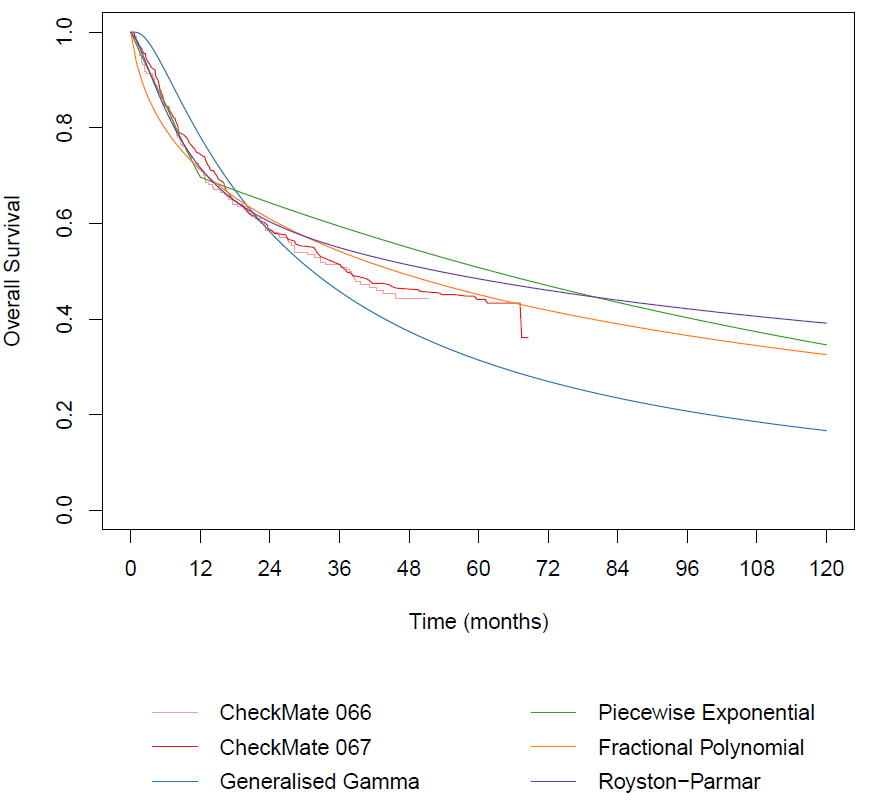


Figure K8: Kaplan-Meier survival curves for CheckMate 066 and CheckMate 067 trials alongside survival estimates from the generalised gamma model with treatment as a location parameter, the piecewise exponential model with a cut point at 12 months, the fractional polynomial model with p=0 and the Royston-Parmar model with treatment-ln(time) interactions for nivolumab.


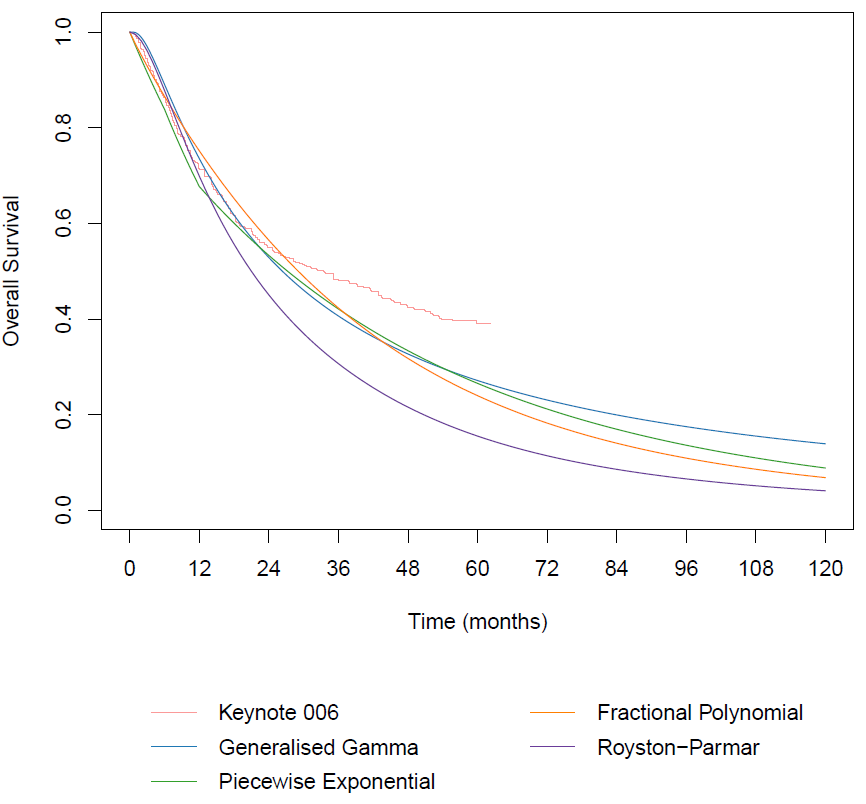


Figure K9: Kaplan-Meier survival curve for the Keynote 006 trial alongside survival estimates from the generalised gamma model with treatment as a location parameter, the piecewise exponential model with a cut point at 12 months, the fractional polynomial model with p=0 and the Royston-Parmar model with treatment-ln(time) interactions for pembrolizumab.


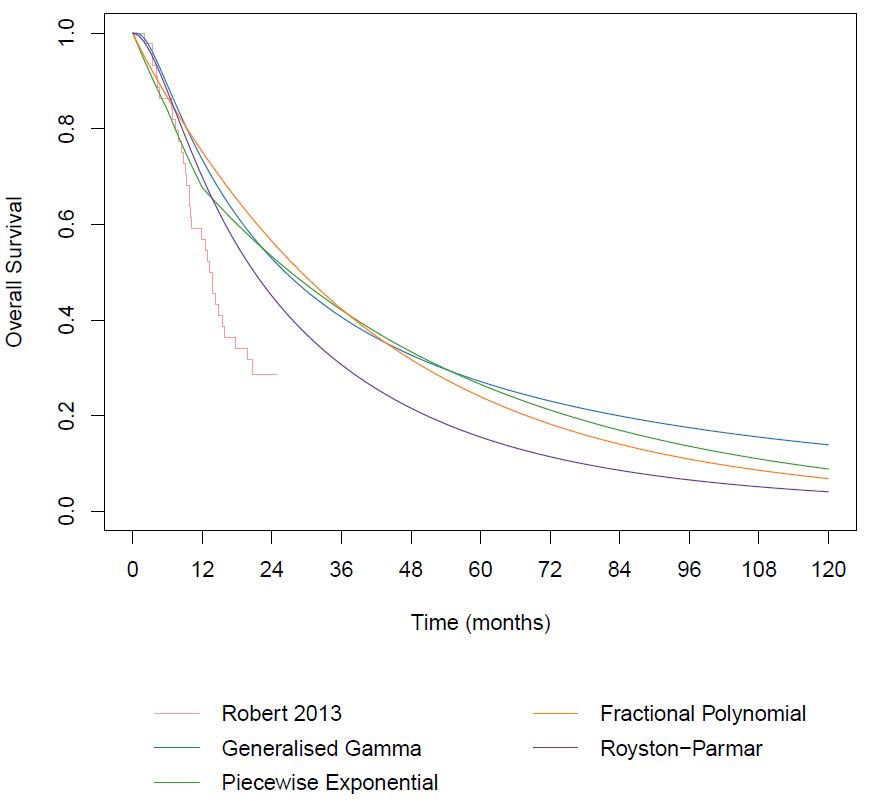


Figure K10: Kaplan-Meier survival curve for the Robert 2013 trial alongside survival estimates from the generalised gamma model with treatment as a location parameter, the piecewise exponential model with a cut point at 12 months, the fractional polynomial model with p=0 and the Royston-Parmar model with treatment-ln(time) interactions for selumetinib plus dacarbazine.


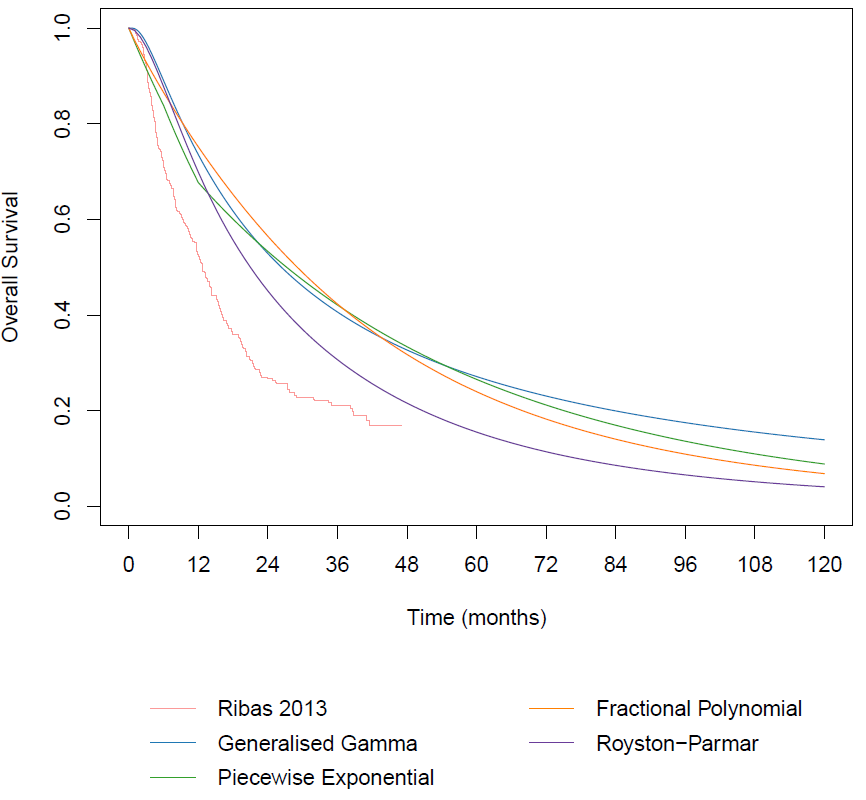


Figure K11: Kaplan-Meier survival curve for the Ribas 2013 trial alongside survival estimates from the generalised gamma model with treatment as a location parameter, the piecewise exponential model with a cut point at 12 months, the fractional polynomial model with p=0 and the Royston-Parmar model with treatment-ln(time) interactions for tremelimumab.


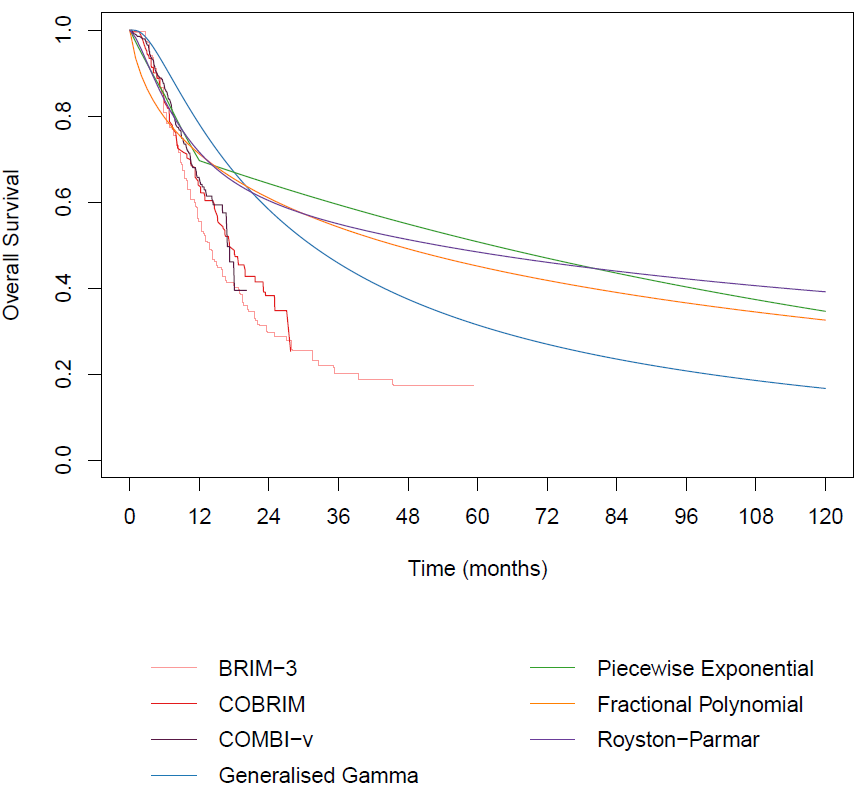


Figure K12: Kaplan-Meier survival curves for BRIM-3, COBRIM and COMBI-v trials alongside survival estimates from the generalised gamma model with treatment as a location parameter, the piecewise exponential model with a cut point at 12 months, the fractional polynomial model with p=0 and the Royston-Parmar model with treatment-ln(time) interactions for vemurafenib.


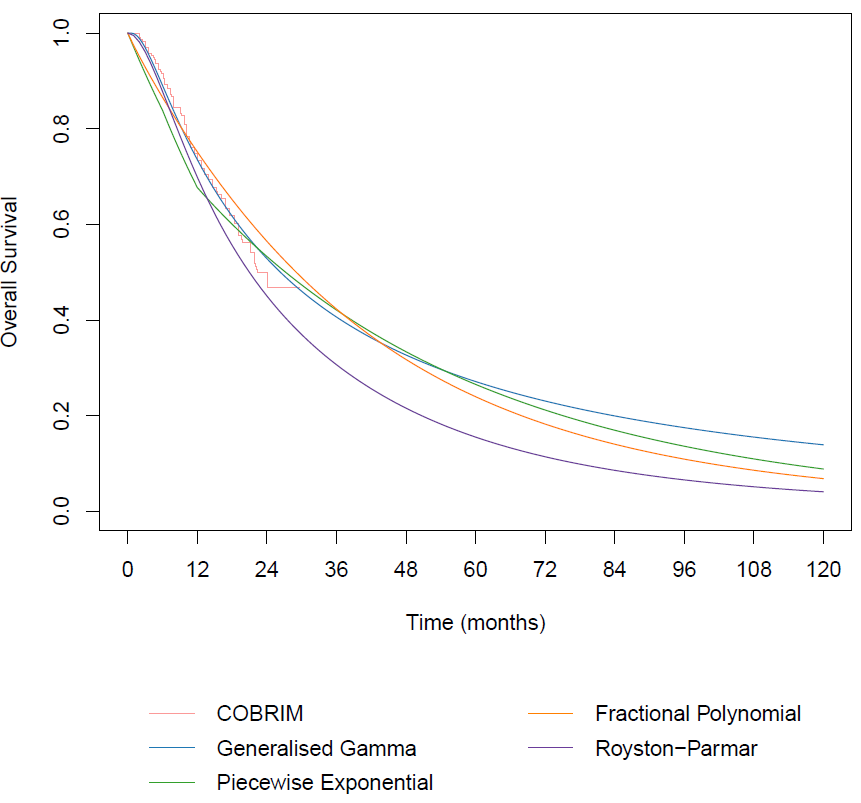


Figure K13: Kaplan-Meier survival curve for the COBRIM trial alongside survival estimates from the generalised gamma model with treatment as a location parameter, the piecewise exponential model with a cut point at 12 months, the fractional polynomial model with p=0 and the Royston-Parmar model with treatment-ln(time) interactions for vemurafenib plus cobmetinib.
